# Emerging roles of prominin-1 (CD133) in the dynamics of plasma membrane architecture and cell signaling pathways in health and disease

**DOI:** 10.1186/s11658-024-00554-0

**Published:** 2024-03-26

**Authors:** Petr Pleskač, Christine A. Fargeas, Renata Veselska, Denis Corbeil, Jan Skoda

**Affiliations:** 1 Laboratory of Tumor Biology, Department of Experimental Biology, Faculty of Science, Masaryk University, Kamenice 5, 625 00 Brno, Czech Republic; 2 International Clinical Research Center, St. Anne’s University Hospital, Brno, Czech Republic; 3 Biotechnology Center (BIOTEC) and Center for Molecular and Cellular Bioengineering (CMCB), Technische Universität Dresden, Tatzberg 47/49, 01307 Dresden, Germany; 4 Tissue Engineering Laboratories, Medizinische Fakultät der Technischen Universität Dresden, Dresden, Germany

**Keywords:** Cancer, Cancer stem cell, Cell signaling, CD133, Cilium, Exosome, Lipid raft, Microvillus, Prominin-1, Stem cell

## Abstract

Prominin-1 (CD133) is a cholesterol-binding membrane glycoprotein selectively associated with highly curved and prominent membrane structures. It is widely recognized as an antigenic marker of stem cells and cancer stem cells and is frequently used to isolate them from biological and clinical samples. Recent progress in understanding various aspects of CD133 biology in different cell types has revealed the involvement of CD133 in the architecture and dynamics of plasma membrane protrusions, such as microvilli and cilia, including the release of extracellular vesicles, as well as in various signaling pathways, which may be regulated in part by posttranslational modifications of CD133 and its interactions with a variety of proteins and lipids. Hence, CD133 appears to be a master regulator of cell signaling as its engagement in PI3K/Akt, Src-FAK, Wnt/β-catenin, TGF-β/Smad and MAPK/ERK pathways may explain its broad action in many cellular processes, including cell proliferation, differentiation, and migration or intercellular communication. Here, we summarize early studies on CD133, as they are essential to grasp its novel features, and describe recent evidence demonstrating that this unique molecule is involved in membrane dynamics and molecular signaling that affects various facets of tissue homeostasis and cancer development. We hope this review will provide an informative resource for future efforts to elucidate the details of CD133’s molecular function in health and disease.

## Introduction

Prominin-1 (Prom1, a.k.a. cluster of differentiation (CD)133; hereafter, CD133 refers to the mammalian molecule) has attracted global interest in the fields of regenerative medicine and oncology, as its expression on the cell surface allows the identification and isolation of stem cells and cancer stem cells (CSCs). In 1997, the discovery of CD133 was reported by two independent research teams studying the cell biology of murine neuroepithelial progenitor cells [1] and the surface markers of human hematopoietic stem and progenitor cells (HSPCs) [2, 3]. Soon after, CD133 protein was detected in fully differentiated cells and cancer cells in both rodents and humans, demonstrating that its expression is not restricted to stem and progenitor cell populations [1, 4–10].

Murine CD133 was originally cloned using a cDNA library prepared from adult kidney [1], while the human ortholog was obtained from retinoblastoma cell lines [3]. Indeed, CD133 is highly expressed in the proximal tubules of the kidney and other epithelial cells in embryonic and adult tissues, where it is expressed solely on the apical domain of polarized cells [1, 7, 11, 12]. CD133 expression has also been identified in the epithelial cells of the epididymal tract, where sperm maturation occurs [10, 13, 14], and in various glands [15–17], such as mammary glands [11, 17, 18], liver [17, 19, 20], pancreas [17, 21] and salivary glands [22–24]. Of note, differentiated nonepithelial cells, particularly photoreceptor cells [8, 23, 25] and glial cells [26], also express CD133, indicating that CD133 plays a general role that is not necessarily linked to a particular condition (e.g., cell stemness or differentiation status) or specific cellular type (epithelial versus nonepithelial) [27].

General interest in CD133 grew exponentially after 2003 when Dirks and colleagues, and others, reported its expression in tumor brain tissues and its use as a marker to isolate human CSCs [28–30]. These reports aroused enormous enthusiasm not only in the field of oncology, where the CD133 expression has been correlated with cancer progression, metastasis, recurrence and poor survival [31, 32] (reviewed in Ref. [33]), but also in that of stem cells. Since then, CD133 has been regularly used as a molecular marker/target to isolate cells with stem cell properties in a wide range of human and murine tissues and tumors (reviewed in Refs [27, 34]). In addition to their detection in neural and hematopoietic systems [1, 35–37], CD133^+^ cells with stem cell properties have been found in healthy and cancerous prostate [38–42], kidney [43–46], liver [47–49], pancreas [50, 51], intestine, colon [52–55], lung [56], and other organ tissues [57, 58]. CD133 was also associated with leukemic cells [3, 5, 59–62]. Importantly, in the context of cancer and regenerative medicine, CD133 is a marker of endothelial progenitor cells that could contribute to tumor vasculature in cancer and tissue regeneration upon injury [63–69]. It cannot be excluded that CD133 plays a role in facultative stem cells, i.e., fully differentiated cells that exhibit stem and progenitor activities through their ability to re-enter the cell cycle in particular tissues and/or under specific conditions [70–72]. In fact, certain differentiated CD133^+^ cells (e.g., in kidney and liver) may have such ability upon injury or in disease states [70] (reviewed in Ref. [73]). Regardless of the mechanism of regeneration, CD133 can mark cells with stem cell properties and thus has clinical value.

The utility of CD133 as an organ-specific stem cell marker in humans and its importance for determining cancer prognosis and progression have been nonetheless called into question [74]. This controversy stemmed in part from the apparent contradiction between the limited expression of CD133 protein in human adult tissues, based on immunodetection using an antibody named AC133 directed against a specific epitope of CD133 (CD133/1) [3], and the wide expression of its transcript as detected via Northern blot and polymerase chain reaction analyses [2, 3, 7, 11, 17, 21, 75]. However, the murine CD133 (both protein and transcript) has been known to be widely expressed well beyond stem cells [1], particularly in epithelial cells (see above) and photoreceptors [8]. The mapping of CD133 to differentiated epithelial cells using LacZ reporter-based mice further contributed to the debate [76]. The use of alternative antibodies against human CD133 has confirmed its wide protein expression [11, 17, 21, 24], similar to that of its ortholog in rodents [9, 10, 22]. Therefore, it is important to note that the AC133 immunoreactivity is not necessarily equivalent to the human CD133 protein, and a link between CD133/1 detection and cellular status (i.e., stem cell versus differentiation) has been proposed [11, 77]. The use of the AC133 antibody and the accessibility of the CD133/1 epitope have been extensively discussed in the literature, and we invite readers to consult the relevant publications [11, 15–17, 78–83].

It should be noted that stem cells are functional without CD133, as illustrated by the various *Prominin-1* (*Prom1*)-knockdown mouse models (reviewed in Ref. [84]) in which no major defects are detected, except for retinal degeneration [23]; this retinal phenotype is consistent with the expression of CD133 in photoreceptor cells [8] (see below). Likewise, no major phenotype, other than blindness, has been detected in patients carrying dominant or recessive mutations in the *PROM1* gene [85], suggesting that CD133 is dispensable for general stem cell properties under physiological conditions [8, 86–88]. For example, total loss of CD133 did not affect the regenerative capacity of mammary epithelium in *Prom-1*^–/–^ mice, although it did impact ductal branching and increased the ratio of luminal to basal cells [18]. This study is in agreement with the earlier report that transplantation of CD133-enriched murine cells from the mammary luminal cell population demonstrated a low regenerative capacity compared with CD133-negative fraction or basal cells, suggesting that in normal mammary tissue, CD133 is not a stem cell marker and that its function goes beyond stem cell activity [89]. Similar conclusions were drawn for mouse oviduct epithelial progenitors [90]. Finally, CD133 is not essential for normal hematopoiesis, as observed in *Prom-1*^–/–^ mice, but nonetheless it modifies the frequencies of growth-factor responsive hematopoietic progenitor cells during steady state and under myelotoxic stress conditions in vivo [91]. These findings suggest that CD133 plays a redundant role in the differentiation of the mature myeloid cell population during hematopoiesis, yet CD133 is important for the recovery of red blood cells after hematopoietic stress [91]. It cannot be ruled out that, in these models, a compensatory mechanism involving the CD133 paralog prominin-2 occurs in CD133-depleted cells in tissues that typically express both proteins [92], which is not the case of retina [93].

Clearly, it is time to re-examine the function of CD133, including its role in cancers, and translate this knowledge into new and biologically relevant CD133-based approaches for tissue engineering, regenerative medicine and cancer therapy. Here, we summarize the current knowledge of the molecular and cellular biology of CD133, including its preferential association with highly curved membrane protrusions such as microvilli and cilia as well as tunneling nanotubes (TNTs) that mediate exchange of CD133 between interconnected stem or cancer cells. We will also discuss the role of CD133^+^ extracellular membrane vesicles (EVs), which have received considerable attention in recent years, and the lessons learned from studies using CD133-deficient animals and CD133-silenced human CD34^+^ HSPCs as well as studies focused on cancers in which CD133 is upregulated. Particular attention will be given to the impact of CD133 on various cell signaling pathways and its potential involvement in cell proliferation, differentiation, autophagy and migration. We hope that this review will promote the development of future functional studies on CD133 as a molecule essential for multiple cellular processes.

## Molecular biology of CD133

### Structure and splice variants

**Fig. 1:**
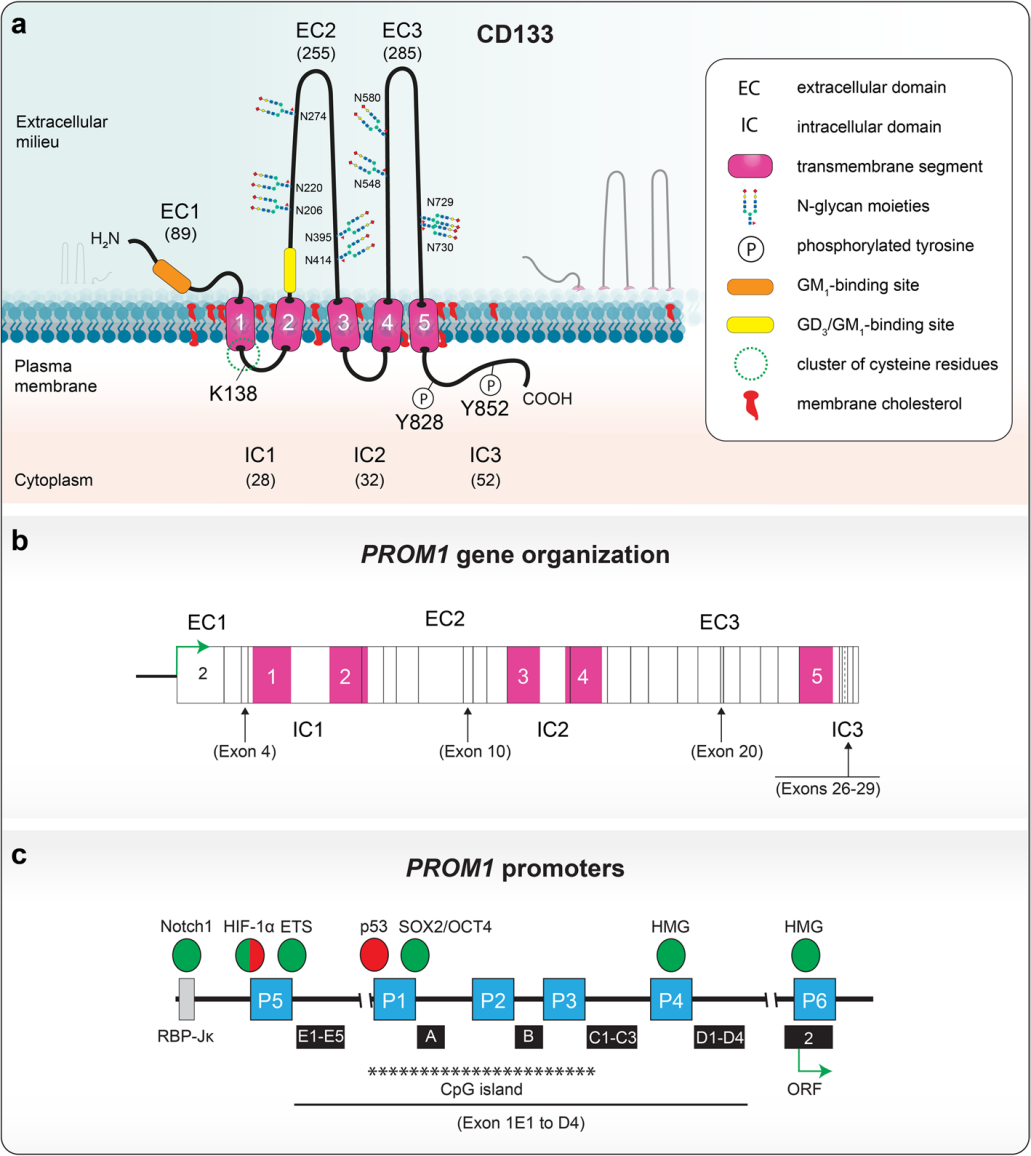
Structural features of CD133. **a** Membrane topology. The human CD133 protein comprises three extracellular domains (EC1–3), an N-terminal domain (EC1) and two larger loops (EC2 and EC3) bearing nine glycosylation sites. N-glycan structures vary with the subcellular localization of CD133 and the state of cell differentiation, which may influence its interaction with protein partners. The intracellular domains (IC1–3) consist of two small cytoplasmic loops (IC1–2) and the C-terminal domain (IC3). ECs and ICs are separated by five transmembrane domains (1–5, pink cylinders). CD133 carries a cluster of cysteine residues located at the boundary of the first transmembrane segment and the IC1 domain (dotted green line). These residues may be subject to palmitoylation. Two potential ganglioside-binding sites are located in the EC1 and EC2 domains (orange and yellow cylinders). Two major tyrosine (Y) residues, 828 and 852, in IC3 can be phosphorylated and regulate the activity of several signaling pathways. Lysine (K) 138 interacts with HDAC6 and Arl13b. The outer and inner leaflets of the plasma membrane are shown with membrane cholesterol (red), highlighting the association of CD133 with cholesterol-dependent membrane microdomains. Amino acid numbering is based on the human splice variant CD133.s2; the approximate number of amino acid residues in a given domain, which may vary from one splice variant to another, is indicated in parentheses. **b** Genomic organization of mammalian CD133. Vertical lines indicate exon boundaries, the dashed line the presence of an alternative splice acceptor site, while transmembrane domains are highlighted in pink. Major facultative exons within ORF are indicated in brackets. The exons are numbered as the initial start codon is located in exon 2. **c** *PROM1* promoters. Six distinct promoters were identified (blue boxes) in human *PROM1* gene with various facultative exons (A-E5, black boxes) that are part of exon 1. The P1-P3 promoters show high proportion of CpG islands. The major transcription factors impacting positively or negatively on *PROM1* gene expression are indicated in green and red, respectively. Figures are not to scale. Illustration in **a** is adapted from Ref [3] and incorporates data from Refs [94, 161, 209, 219], while those in panels b and c are adapted from Refs [95] and [111], respectively

CD133 is a membrane glycoprotein with an apparent molecular mass of ≈ 120 kDa, of which N-glycans represent ≈ 20 kDa, consistent with its predicted molecular weight of 97,202 Da (referring here to the human splice variant s2, see below) [1, 3, 11]. It contains five transmembrane segments; delimiting an extracellular N-terminal domain (referred to as EC1), two large extracellular loops (EC2 and EC3) alternating with two small intracellular loops (IC1 and IC2) and an intracytoplasmic C-terminal domain (IC3). The approximate size of each structural domain is indicated (Fig. 1a, see the corresponding legend for more details) [22]. Eight asparagine residues in consensus N-glycosylation sites (Asn-X-Ser/Thr-X sequons, where X is any amino acid except proline) are found in human and murine CD133 and distributed between EC2 and EC3 [1, 3]. An additional site, Asn_206_-Glu-Thr-Pro, was shown to be glycosylated in human CD133 [94].

CD133 belongs to the prominin family of pentaspan membrane proteins [93]. Two distinct mammalian *Prominin* genes have been described [93, 95], while three prominin molecules have been identified in nonmammalian species [25, 96, 97]. Two *Prominin*-related genes, prominin and prominin-like, were identified in *Drosophila melanogaster* [93, 98–100] and other holometabolous insects [101]. However, in contrast to prominin-like, fly prominin has a predicted sixth transmembrane domain [101, 102]. Interestingly, the *Prom1* gene is duplicated in zebrafish, and the two gene products are referred to as *prominin-1a* and *-1b* [25, 103]. Structurally related prominin-like proteins have been identified in an amoeba, i.e., *Naegleria gruberi* (GenBank accession numbers JN679227.1 and JN679228.1), suggesting the expression of prominin in unicellular organisms. Interestingly, amino acid sequences are poorly conserved between paralogs as well as within one orthologous group, notably CD133/prominin-1. For example, only 60% identity has been observed between primates and rodents, while their sequence identities with other species (fish, amphibians, birds) are below 50%; and less than 25% with invertebrates (flies, worms) [25, 93, 96]. Yet, the analysis of the primary sequence of all prominin molecules, regardless of the species, revealed no potential enzymatic/catalytic motif or domain that could explain their molecular function [95].

*PROM1*/*Prom1* genes are located on chromosome 4 and 5 in humans and mice, respectively (see also Online Mendelian Inheritance in Man (OMIM) entry number 604365). The organization of these genes and of the *PROM2* genes (OMIN 617150) is highly conserved in terms of exon–intron boundaries, across most of species (Fig. 1b) [25, 93, 104, 105]. At least six alternative promoters (named P1-P6; discussed in detail below) have been identified in the human *PROM1* gene. The primary transcripts may undergo extensive alternative splicing [8, 10, 106]. Considering mammalian and nonmammalian vertebrates, more than 20 splice variants from at least 28 exons affecting the open reading frame (ORF) have been described [10, 25, 105]. In rodents and primates, the resulting proteins would be from 804 to 865 amino acids in length [95]. A nomenclature of CD133 splice variants proposing to add a suffix “s” and number the variants according to the chronology of publication regardless of species was presented [10, 25, 77, 104]. The majority of these splice variants differ in the N- and C-terminal domain sequences. Splice variants s1 and s2 differ from each other by the inclusion or exclusion of a small exon [numbered 4 as we refer to exon 2 as carrying the initial codon] in EC1, respectively, while IC3 shows a greater propensity for alternative splicing of small exons (exons 26–29), suggesting interactions with distinct extracellular and cytoplasmic partners (Fig. 1b, see legend for more details) [10, 104]. At least 10 distinct cytoplasmic C-termini were described [25] (reviewed in Ref. [95]). The final four C-terminal amino acid residues in some variants exhibit the characteristics of PSD-95/Dig-1/ZO-1 (PDZ)-binding domains (classes I-III), which is consistent with the ability of the CD133 variants to bind various proteins [104].

The expression of CD133 splice variants is often cell- and tissue-type dependent and may reflect its particular role in the given organ [10, 26, 34, 107]. For example, CD133.s1 is predominantly expressed in the brain tissue of mouse embryos, and its expression decreases during brain development to barely detectable levels in the brains of adult mice [26]. An opposite trend was observed for CD133.s3 expression in the early postnatal period, which correlated with the onset of neuronal myelination. This splice variant is a component of the myelin sheath [22, 26], and hypomyelination has been observed in *Prom1*-null mice [108]. Exons splicing in the EC2 appears to impact cell surface expression and overall folding of the protein, as evidenced by the absence of the 25 residues encoded by murine exon 10 leading to the CD133 degradation in the endoplasmic reticulum (ER) (Fig. 1b) [10]. A 16-kDa truncated variant of the CD133 protein has also been reported in glioblastoma cell lines [109]. Yet, the precise nature of this potential short form would require further investigation. Importantly, the coexpression of distinct CD133 splice variants has been reported [10], confirming the need to include systematic analyses of CD133 variants in future studies to unravel their potentially complex involvement in various cellular processes.

### Regulation of PROM1 gene expression

In relation to its multiple roles and differential expression in normal tissues, stem cells and cancer cells, the transcriptional regulation of CD133 expression is complex with human *PROM1* gene being driven by six promoters [106, 110–112]. Consequently, at least 14 distinct transcripts are generated depending on the tissue with different or optional exons 1 (namely 1A-E) constituting the 5’ untranslated region (UTR) (Fig. 1c) [106, 111, 113]. The P1, P2 and P3 promoters of *PROM1* gene show high proportion of CpG islands and are differentially methylated in normal and cancer tissues such as glioma or colon cancer [110, 114–117]. These promoters may also be polycomb-repressed in acute leukemia [118] and divers cell lines [111]. The proximal P6 promoter identified in melanoma cells was found to be enriched in binding sites for high mobility group (HMG) proteins—nonhistone chromatin-associated proteins that are aberrantly expressed in a variety of cancers (Fig. 1c) [111].

*PROM1* gene expression may also be regulated through histone modification, which may depend on DNA hypermethylation [114, 117]. Histone H3 lysine-79 (H3K79) methylation was first identified to be a regulator of *PROM1* gene expression in an investigation aimed at identifying targets of mixed lineage leukemia (MLL) fusion proteins responsible for aberrant gene expression in patients with leukemia [119]. *PROM1* gene was found to be a target of the MLL fusion-associated gene AF4 (MLL-AF4) in human colon cancer Caco-2 cells [120] and in some MLL cells [118], where its transcription is upregulated through H3K79 methylation and the presence of an intragenic H3K79me2/3 enhancer element. Notably, polycomb-repression inactivates such enhancer. Transforming growth factor (TGF)-β1 has been shown to induce the demethylation of *PROM1* promoter P1 by inhibiting the expression of DNA methyltransferase-1 and 3β (DNMT1 and DNMT3ß) in hepatoma cells, leading to a significant upregulation of CD133 [121]. Collectively, TGF-β signaling in different solid malignancies leads to the induction of stem-like characteristics [122], the epithelial-mesenchymal transition (EMT), or increased tumorigenicity [123] and initiates the expression of CD133. The mechanisms that regulate the interplay between TGF-β1 and CD133, thereby contributing to the stem cell phenotype among various normal and cancer cell types, remains to be explored in greater detail.

Interestingly, a thorough analysis of the relationship between promoter hypomethylation and increased CD133 expression in glioma revealed novel transcriptional coregulators of CD133 expression, specificity protein 1 and c-MYC, which can bind only to a hypomethylated *PROM1* promoter [110]. However, although such epigenetic regulation has been shown to exist in prostate cell lines, this is not the case in primary prostate epithelial cultures, suggesting evidence for dysregulated CD133 expression during long-term culture in vitro [124]. In basal-like breast carcinoma cells with p53 deficiency, which leads to an autocrine interleukin (IL)-6 loop driving cell reprogramming, IL-6 was found to regulate *PROM1* expression by inducing *PROM1* P1 promoter demethylation that resulted in enhanced transcription and, in parallel, an increased methylation of the *PROM1* P2 promoter that carries putative repressor sites [125].

Cells exposed to stresses, such as DNA damage, hypoxia, oncogene activation, or ribosomal stress, react by stabilizing p53, which in turn orchestrates the transcription of genes involved in major stress response processes, i.e., cell cycle arrest, DNA repair, and cell death [126]. The expression pattern of CD133 was reported to be inversely related to the expression of p53 in different cancer cell lines and tumor tissue samples [127]. Noncanonical p53 binding sites were identified in the P1 promoter enabling p53 interaction (Fig. 1c), which led to the recruitment of histone deacetylase (HDAC) 1 and thus inhibition of CD133 expression due to reduced histone H3 acetylation. Interestingly, the downregulation of CD133 was also accompanied by suppression of stemness-associated transcription factors, such as NANOG, octamer-binding transcription factor 4 (OCT4, also called POU5F1), sex-determining region Y-box 2 (SOX2), and c-MYC, and reduced cell growth and tumor formation capacity [127].

**Fig. 2:**
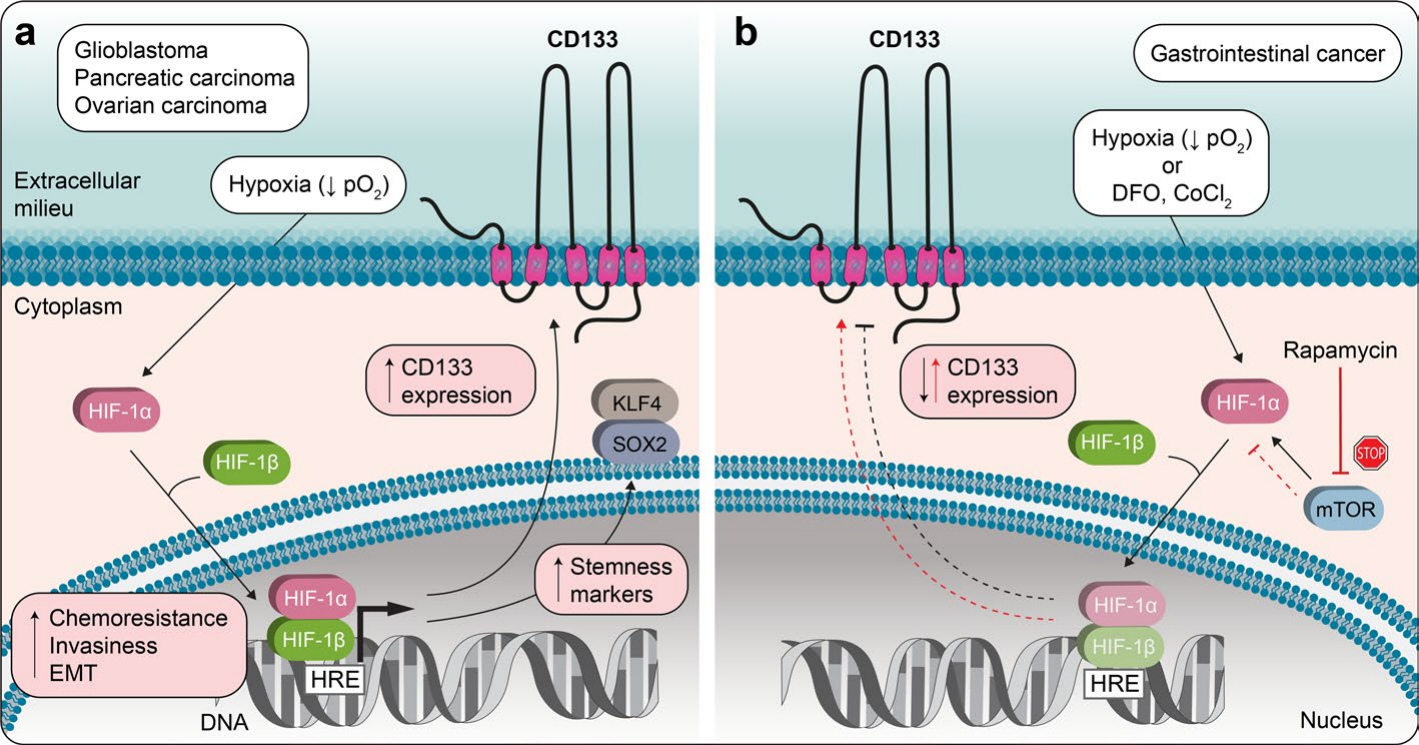
Cell type-dependent impact of hypoxic conditions on CD133 expression. **a** Under hypoxic conditions and in certain types of cancer as indicated, HIF-1α and HIF-1β are translocated into the nucleus, where they form a heterodimeric transcription factor that binds to hypoxia-responsive elements (HRE), resulting in increased expression of CD133 and other stemness markers (e.g., KLF4 and SOX2). Thus, hypoxia can promote chemoresistance, invasiveness and EMT. **b** In gastrointestinal carcinoma cells, the induction of hypoxia either by lowering the partial pressure of oxygen or by applying hypoxia-mimicking agents such as DFO and CoCl_2_ downregulates CD133 expression (black dashed arrow). Reciprocally, rapamycin-mediated inhibition of mammalian TOR (red dashed arrow), an upstream regulator of HIF-1α signaling, results in the downregulation of HIF-1α and elevated levels of CD133 (red arrow). Illustrations in panels a and b are based on data presented in Refs [130] and [139], respectively, among others

Hypoxia is a key factor in the tumor microenvironment and was shown to increase the CD133^+^ population in medulloblastoma and glioma cells [128, 129]. Prolonged hypoxia exposure (i.e., 1% O_2_ for a period of 72 h) stimulated glioblastoma cells to express CD133 and the stemness markers Kruppel-like factor 4 (KLF4) and SOX2 via a hypoxia inducible factor (HIF)-1α-dependent mechanism (Fig. 2a) [130]. Interestingly, these stemness traits as well as the significantly higher clonogenicity and capacity to form spheres during serial passaging were maintained after normoxic conditions were restored [130]. A similar hypoxia-induced coexpression of CD133 and HIF-1α in glioma cells has been reported by other groups [131, 132], and it was shown to be associated with enhanced chemoresistance, invasiveness, and EMT, which is in line with results obtained with cell lines derived from pancreatic [133] and ovarian cancer samples [134]. HIF-1 is a heterodimeric protein that regulates tissue responses to changes in the oxygen level [135]. It is composed of two subunits, α and β. Although HIF-1β subunit expression is constitutive, HIF-1α subunit expression is regulated by the partial pressure of oxygen level. Under normoxic conditions, the levels of HIF-1α are reduced because of proteasomal degradation. Hypoxia induces the dimerization of HIF-1α and HIF-1β, which form a transcription complex that recognizes E-box-like hypoxia response elements and initiates the transcription of genes involved not only in cellular oxygen homeostasis but also in pathways that coordinate cell proliferation, metabolism reprogramming, apoptosis, or resistance to tumor therapy [135].

Elevated levels of HIF-1α and HIF-2α were shown to simulated the transcription of the *PROM1* gene at the P5 promoter in colon cancer cells; specifically, it was initiated by the binding of the HIF-1-Elk1 complex to the E-twenty-six (ETS)-binding motif present in P5 [113, 136]. In contrast, in lung cancer cell lines, hypoxia-induced CD133 transcription was mainly initiated via HIF inducible-OCT4 and SOX2 binding at the P1 promoter that is devoid of HIF-binding site [137]. Interestingly, in hepatocellular carcinoma, a functional cooperation between IL-6/signal transducer and activator of transcription 3 (STAT3) signaling and nuclear factor kappa-light-chain-enhancer of activated B cells and HIF-1α was reported to upregulate CD133 expression that was associated with poor prognosis [138].

However, in addition to the positive regulation of CD133 under hypoxic conditions, contradictory results have also been reported for different tumor types. Induction of hypoxia, either by lowering the partial pressure of oxygen or by applying hypoxia-mimicking agents [e.g., desferrioxamine (DFO) and cobalt chloride (CoCl_2_)] in three gastrointestinal carcinoma cell lines, suppressed the expression of CD133 (Fig. 2b) [139]. In line with these findings, rapamycin-mediated inhibition of mammalian target of rapamycin (TOR), an upstream positive regulator of HIF-1α signaling, resulted in downregulation of HIF-1α and upregulation of CD133 transcription [139]. This negative correlation between CD133 and HIF-1α expression may be tissue specific. A focus on potential interaction partners of the HIF transcription complex in future studies may help to decipher the mechanisms responsible for the different impacts of hypoxia on CD133 expression.

Notch1 has also been shown to control the proportion of CD133^+^ cells in lung adenocarcinoma [140] and to regulate CD133 expression in glioblastoma [141]. Konishi and colleagues reported that in diffuse gastric cancer, CD133 expression was induced by Notch1 through the binding of the activated Notch1 intracellular domain to a recombination signal binding protein for immunoglobulin kappa J region (RBP-Jκ)-binding motif identified in the *PROM1* gene promoter (Fig. 1c) [142]. In melanoma cell lines from both mice and humans, Notch1 also induced CD133 expression by the binding of activated Notch1 to the *Prom1/PROM1* promoter [143].

The *PROM1* gene promoter sequence carries a putative tandem β-catenin-T-cell factor (TCF)/lymphoid enhancer factor (LEF) complex binding sites within intron 2 that are conserved among mammalian *PROM1* genes [144]. Moreover, specific inhibition of CREB-binding protein (CBP), a coactivator of β-catenin/TCF-mediated transcription [145], also suppressed CD133 expression at both the mRNA and protein levels in hepatocellular carcinoma cells, reducing the anchorage-independent growth and colony formation capacity of these cells [146]. These observations suggest that *PROM1* is a Wnt target gene, which may allow a feedback loop between CD133 and β-catenin-Wnt signaling, as is discussed below.

Posttranscriptional regulation of CD133 expression by microRNAs (miRNAs) has also been documented, and these findings are in line with CD133 expression in CSCs, as these short noncoding RNAs play important roles in cancer initiation and progression and in the control of signaling pathways activity [147, 148]. For instance, miR-29b downregulated *CD133* mRNA by targeting its 3’-UTR in transfected human hepatocellular carcinoma cells [149]. Similar observations were made in esophageal cancer cells, where miR-377 expression was inversely correlated with CD133 expression [150]. High levels of miR-181a inhibited CD133 in glioblastoma cells; however, whether the miRNA acted directly or indirectly remains to be determined [151].

Interestingly, the 3’-UTR of human *CD133* transcripts bears a noncanonical iron-responsive element that may mediate their stabilization after the binding of cytosolic iron-regulatory protein 1, a key controller of iron metabolism that posttranscriptionally regulates the expression of iron metabolism genes. This possibility is in line with the reduced CD133 protein levels observed in Caco-2 cells after treatment with iron chelators or iron supplementation [152]. This regulatory mechanism appears to be related to the cholesterol-dependent negative impact of CD133 on transferrin uptake through endocytosis observed in undifferentiated Caco-2 cells. As transferrin plays the major role in the delivery of iron to cells via endocytosis, it may interfere with hypoxia-induced regulation of CD133 expression. These data justify interest in further studying the CD133/transferrin-iron network and its potential role in endocytosis [152].

Overall, it appears that, in relation with the implication of CD133 in different biological and pathological processes, such as cancer and degenerative diseases, numerous gene expression regulatory mechanisms confer the diverse expression patterns of CD133.

### Posttranslational modifications of CD133

In addition to alternative splicing, CD133 undergoes various posttranslational modifications that regulate its intracellular trafficking, stability and interactions with cytoplasmic enzymes and/or other classes of proteins. One of these CD133 modifications is N-glycosylation, in which differential processing of the glycan moiety can lead to distinct glycosylation profiles between tissues and organs [10]. Different glycoforms may coexist in a given tissue, but little is known about these complex structures. The terminal N-glycans of human CD133 contain sialyl residues, which seem to regulate CD133 stability in neural stem cells and glioma-initiating cells [153]. In the mouse embryonic brain, N-glycans of CD133 bind to *Phaseolus vulgaris* erythroagglutinating lectin, which allows cells with stem cell properties to be isolated [154]. Notably, the CD133 glycosylation is altered during early pregnancy in uterine epithelial cells under the influence of maternal ovarian hormones [155].

Similarly, hypoxic conditions can influence the glycosylation status of CD133, as demonstrated with pediatric glioblastoma cell lines [156]. Interestingly, the glycosyltransferase 8 domain containing 1 (GLT8D1) was recently shown to contribute to the stabilization of CD133 by interacting with it and influencing its glycosylation in glioma cells [157]. Similarly, the interaction of the high-mannose N-glycan form of CD133 with cytoplasmic DNA methyltransferase 1 (DNMT1) maintains the slow-cycling state of glioma stem cells, and favors chemotherapy resistance and tumorigenesis [158]. These observations are in line with the differential glycosylation status of CD133 in relation to cell differentiation [7, 11]. Although DNMT1 interaction is mediated by CD133 cytoplasmic C-terminal domain, the mechanism underlying the contribution of high-mannose N-glycans of CD133 remains unclear. The mutation of individual N-linked glycosylation sites in CD133 had no effect on its stability [159], although the loss of N-linked glycosylation at Ans548 decreased the ability of CD133 to associate with β-catenin and activate the β-catenin signaling pathway, and thus reduced CD133-driven cell proliferation [94].

Similar to differential splicing, alternative glycosylation patterns should be considered when selecting specific antibodies against CD133, especially those used for its immunodetection in a tissue of interest. For example, N-glycosylation of human CD133 seems to contribute to the recognition of the CD133/1 epitope on the cell surface by the AC133 antibody [159] (see above). Therefore, the use of two distinct antibodies to analyze CD133 expression in a given tissue and/or under specific physiological and pathological conditions is recommended [11, 17, 21–24, 83, 160].

Importantly, CD133 is also subject to other posttranslational modifications, such as phosphorylation, ubiquitination, and acetylation [24, 161, 162]. Phosphorylation, as one of the reversible posttranslational modifications, is essential for the regulation of protein functions in cell signaling. Human CD133 can be phosphorylated at two distinct tyrosine residues (namely, Y828 and Y852 in the human CD133.s2 sequence) in its cytoplasmic C-terminus (Fig. 1a) [161]. The phosphorylation of these residues is mediated by the Src and Fyn tyrosine kinases, members of the Src nonreceptor tyrosine kinase family [161]. The amino acid sequence flanking Y828 is highly conserved in vertebrates and conforms to the tyrosine kinase phosphorylation motif [R/K]xxx[D/E]xxY. Y828 is found in the YDDV Src SH2-binding motif, and its phosphorylation may regulate the CD133 interaction with SH2 domain-containing proteins involved in intracellular signaling events [161]. A highly significant activity mediated by Y828 phosphorylation of CD133 is its interaction with the p85 regulatory subunit of phosphoinositide 3-kinase (PI3K), which thereby regulates PI3K activity at plasma membrane [163]. After overexpression, both Src and Fyn enzymes induced tyrosine phosphorylation of the complex N-glycosylated form of CD133 associated with the plasma membrane, but only Src modified the high-mannose N-glycan form associated with the ER [161]. The significance of this selectivity as well as whether Src-dependent phosphorylation of the high-mannose form of CD133 contributes to its interaction with DNMT1 (see above) remains to be determined.

Y852 is encoded by a facultative exon, the sequence of which is relatively less conserved and does not conform to the phosphorylation motif or the SH2-binding domain. It is present in some mammals, including rats, mice, chimpanzees, and humans [9, 104], but not in others, such as dogs [105]. Nevertheless, the phosphorylation of Y852 has been shown to play a critical role in the activation of Src-focal adhesion kinase (FAK) signaling [164].

Ubiquitination is another posttranslational modification of CD133 [24] that might regulate its internalization from the cell surface and sorting into small intralumenal vesicles within late endosome/multivesicular bodies (LE/MVB) *en route* to exosomes [165]. Lysine 848 (K848) is one of the sites of CD133 ubiquitination. Ubiquitinated CD133 interacts with the tumor susceptibility gene 101 (TSG101) protein [165], a component of the endosomal sorting complex required for transport (ESCRT) machinery involved in LE/MVB formation [166], and possibly with syntenin-1 [24], a PDZ domain-containing scaffold protein that regulates the biogenesis of exosomes in conjunction with ALIX and syndecan (see below) [167]. Together, these findings suggest a role for ubiquitination in the intracellular trafficking of CD133 and its release in association with exosomes into physiological bodily fluids (see below). CD133 is also subjected to acetylation by acetyltransferase 1 and 2, two acetyl-CoA:lysine acetyltransferases associated with the ER/ER–Golgi intermediate compartment. Three lysine residues (K216, K248 and K255) in the EC2 are acetylated during the anterograde transport of CD133 to the plasma membrane, and perturbation of this posttranslational modification affects the stability of CD133 and impedes its appearance at the cell membrane [162, 168].

## Cellular biology of CD133

### CD133: an organizer of plasma membrane protrusions

Prominin-1 owes its name to its specific subcellular distribution on prominent cellular protrusions [1]. It localizes to highly curved membrane subdomains, such as microvilli, cilia, filopodia, and other membrane structures that protrude from flat regions of the plasma membrane regardless of cell type, i.e., stem cells versus differentiated cells, or epithelial versus nonepithelial cells, as described in a review article published two decades ago [22]. Its expression in the flagellum of immature spermatozoa present in murine testis and in the myelin sheath produced by oligodendrocytes and Schwann cells is consistent with its specific localization to membrane protrusions [10, 26].

The importance of this subcellular localization was initially demonstrated in photoreceptor cells in which CD133 is enriched at the base of the outer segment of rod cells [8]; more specifically, in precursor membranes of photoreceptor disks emerging from the connecting cilium (reviewed in Refs [169, 170]). The outer segment is a specialized ciliary organelle that allows sensory neurons to detect light and convert it into cellular signals relayed to downstream neurons [171]. The interaction of CD133 with the membrane protein protocadherin 21 regulates the proper biogenesis and maintenance of these large nascent membrane evaginations [87]. CD133 knockdown in murine models impaired these processes, leading to the disorganization of the photoreceptor outer segment and progressive degeneration of the photoreceptor [23]. Variations in the genetic background could influence the progression of photoreceptor cell degeneration [172]. Han and colleagues demonstrated that frog prominin-1 localized to highly curved open rims of outer segment lamellae in the rod and cone cells of *Xenopus laevis* retinas [173]. Retinal phenotypes were observed in frogs and zebrafish when prominin-1 or prominin-1b paralog was silenced, respectively [174, 175]. Although zebrafish prominin-1a is also highly expressed in photoreceptors, its role has not been established [25, 175, 176]. As observed in frog eyes, CD133 was expressed throughout all the disk membranes of human cone cells [170], and clinically, all patients with recessive or dominant *PROM1* mutations show cone–rod dystrophy. Interestingly, although the recessive diseases were associated with early-onset severe panretinal degeneration with early central loss of vision, the dominant diseases were linked with late-onset dystrophy predominantly involving the macula [85].

**Fig. 3:**
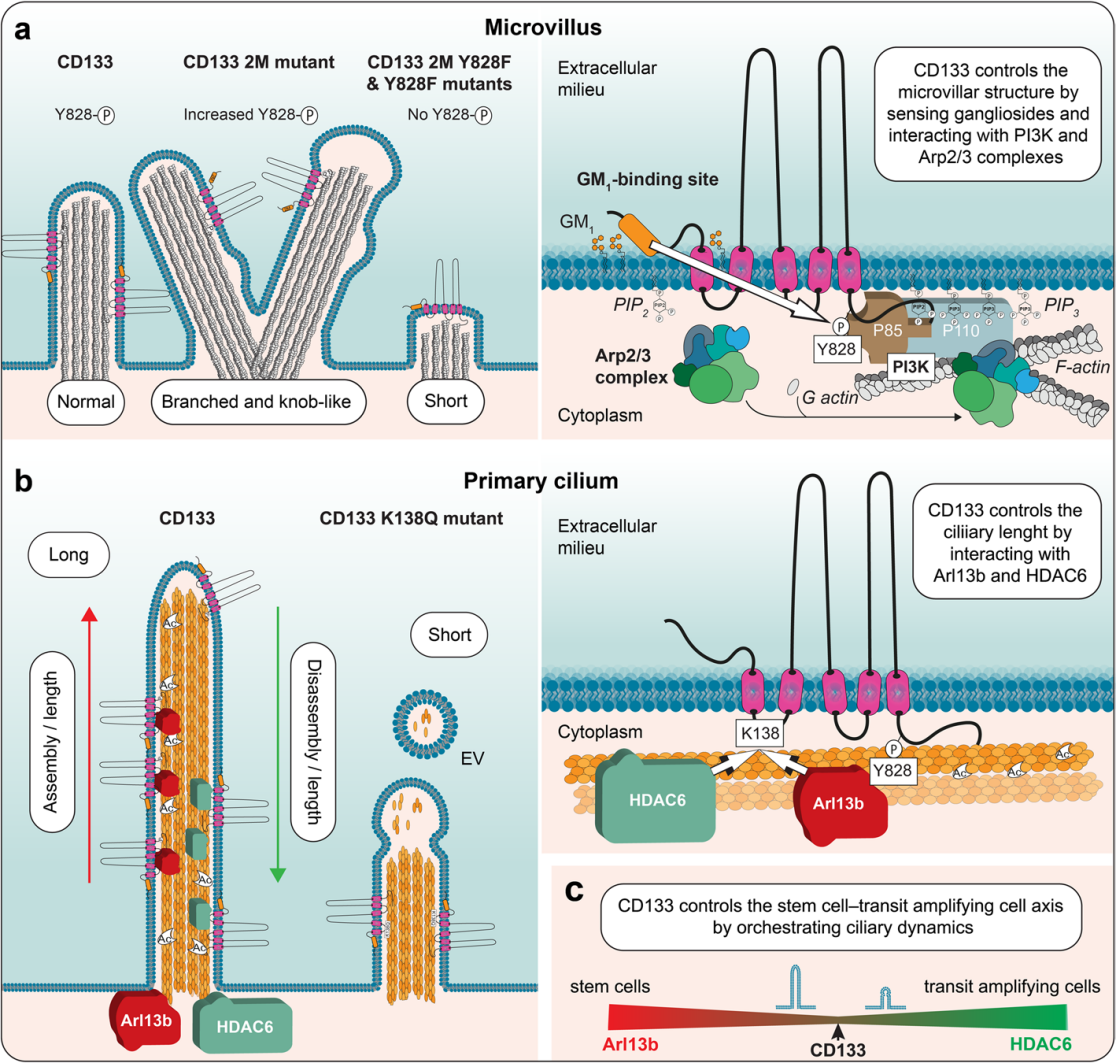
CD133 impacts the architecture of actin-based microvilli and microtubule-based primary cilia. **a, b** CD133 is involved in the architecture of microvilli (**a**) and primary cilia (**b**), two distinct types of membrane protrusions based on actin filaments and microtubules, respectively. In microvilli, mutation (2 M) in the GM_1_-binding domain of CD133 creates branched microvilli and/or microvilli with a knob-like structure, while the mutation of tyrosine 828 by phenylalanine (2 M Y828F or Y828F mutants; numbered according to CD133.s2) abolishes these phenotypes and creates short microvilli (**a**, left panel). Branching of the microvilli results from the interaction of phosphorylated CD133 with the Arp2/3 complex, whereas the interaction with PI3K, which stimulates the conversion of PIP_2_ to PIP_3_ in the inner leaflet of the plasma membrane, uncouples the plasma membrane and the underlying cytoskeleton, resulting in irregularly shaped microvilli (**a**, right panel). Both subunits (p85 and p110) of PI3K and all seven subunits of the Arp2/3 complex are represented. In primary cilium, overexpression of CD133 increased cilium length, while K138Q mutation led to the formation of short cilia and the appearance of EVs derived therefrom (**b**, left panel). The dual interaction of CD133, mediated by K138, with Arl13b and HDAC6 regulate the assembly and disassembly, respectively, of the ciliary structure. Arl13b binding to CD133 also depends on tyrosine 828 phosphorylation (**b**, right panel). Ac, acetylated tubulin. **c** CD133 plays an essential role in the recruitment of molecular regulators (Arl13b and HDAC6) controlling the dynamics of the ciliary compartment during the activation of quiescent stem cells into transit amplifying cells, as demonstrated in incisor tooth epithelial cells—a process impaired in CD133-null mice. Illustrations in **a**–**c** are based on data presented in Refs [177, 182, 183]

The structural impact of CD133 expression on membrane protrusions concerns more than disk morphogenesis in photoreceptor cells, as recently demonstrated in one of our laboratories. For example, overexpression of human CD133 in polarized Madin-Darby canine kidney (MDCK) cells resulted in an increase in the number of microvilli, branched microvilli and microvilli clusters within the apical surface (Fig. 3a), whereas its silencing in human CD34^+^ HSPCs abolished uropod-associated microvilli-like structures at the rear pole [177]. Similarly, CD133 overexpression impacted the structure of filopodia in fibroblasts or other membrane extensions in retinal pigmented epithelium cells [177, 178] (see below). The interaction of CD133 with the actin-related protein 2/3 (Arp2/3) complex, which mediates the branching of actin networks, may favor such microvilli-related and filopodial alterations in epithelial and nonepithelial cells, respectively (Fig. 3a, see below) [177]. The latter case might explain, at least partially, the involvement of CD133 in cancer metastasis, as both CD133 and actin filament branching are found at the leading edge of motile cell lamellipodia, which are the driving force of cell migration [1, 179]. The interaction between CD133 and the Arp2/3 complex is mediated by the phosphorylation of CD133 Y828, and the mutation of this tyrosine (Tyr → Phe, Y828F) resulted in short microvilli (Fig. 3a) [177]. The CD133-Arp2/3 complex interaction is also of interest in the context of photoreceptor biogenesis since it could stimulate the growth of membrane evaginations from the connecting cilium at the base of the outer segment to generate a new photoreceptor disk [180].

The implication of CD133 in ciliary structures has been reported in different cell types. The spatiotemporal activation of stem cells is based on coordinated cell signaling. The primary cilium, acting as a sensory organelle, participates in the transmission of extracellular signals into a cell, triggering downstream cascades responsible for the cell renewal and differentiation [181]. The proper function of the primary cilium depends on the balanced assembly and disassembly of the microtubular apparatus through the cell cycle. Accumulating evidence shows that CD133 plays a critical role in controlling the length of the primary cilium in both mammalian and nonmammalian vertebrates [182, 183], via either its interaction with regulators of ciliary morphology or CD133^+^ EV budding from the ciliary membrane (Fig. 3b). The distribution of CD133 within the ciliary compartment is complex and often asymmetrical along the axoneme [182, 184, 185]. CD133 is found either at the base or the tip of a cilium or within the ciliary shaft [182, 183]. A good example of the physiological involvement of CD133 in ciliogenesis was demonstrated by Singer and colleagues using dental epithelium stem cells as a model, showing that CD133 controls the stem cell–transit amplifying cell axis by orchestrating ciliary dynamics, which was disrupted in *Prom-1*^–/–^ mice (Fig. 3c) [182]. The mechanism by which CD133 regulates primary cilium length has been further dissected using kidney MDCK cells [182, 183]. Two regulators of ciliary morphology have been shown to interact with CD133 [182]: ADP-ribosylation factor-like GTPase 13B (Arl13b) and HDAC6. Both of these CD133-interacting proteins compete for cytoplasmic K138 (numbered as in splice variant s2) in the CD133 IC1 [182]. Arl13b is a member of the Ras superfamily of small GTPases that regulates ciliary length [186, 187], while HDAC6 catalyzes the deacetylation of alpha-tubulin and is involved in the disassembly of the primary cilium, a process required for cell cycle progression (Fig. 3b) [188, 189]. The dual interaction of CD133 with Arl13b and HDAC6 may orchestrate cilium functionality and ciliary length dynamics in a positive and negative manner, respectively, and consequently regulate the activation of dental stem cells (Fig. 3c). Of note, the phosphorylation of Y828 has also been implicated in the CD133-Arl13b interaction, and its mutation resulted in a reduction in ciliary length and the number of cells with a primary cilium [183]. In such context, it remains to be determined whether CD133 phosphorylation impacts its interaction with HDCA6, as suggested in the process of autophagy [190] (see below), or whether other posttranslational modifications that would promote or hinder CD133-Arl13b/HDAC6 interactions control the ciliary architecture, and functionally influence cellular proliferation versus differentiation.

Beyond dental stem cells, ciliary CD133 may have an impact on the activity of other cells with stem cell properties, including those associated with the nervous system [191–196] (reviewed in Ref [197]). In addition to primary cilia, CD133 affects motile cilia, such as those found in ependymal cells or multiciliated cells of the airway epithelium, as the absence of CD133 impaired ciliary beating [198, 199]. CD133 is also associated with multiciliated cells found in oviduct epithelium [90]. In zebrafish, *prominin-3* silencing alters the number and length of monocilia in Kupffer’s vesicles, resulting in molecular and anatomical defects in left–right asymmetry [183]. Thus, the involvement of CD133 (or its paralogs) in ciliogenesis and/or ciliary functions may have consequences in many ciliopathies [200].

The association of CD133 with cellular protrusions and its involvement in their proper organization is not unique to vertebrate cells, as *Drosophila melanogaster* Prominin has been found in the microvilli-based rhabdomere of the photoceptor cells. Therein, Prominin concentrated at the apical tips of microvilli, and by interacting with the secreted protein Eyes Shut/Spacemaker, it prevented unwarranted contacts between adjacent membrane protrusions and conferred structural integrity onto rhabdomeres [98]. The knockdown of fly Prominin led to the altered arrangement of the photoreceptor compartment. Yet, this phenotype was rescued by the expression of mammalian CD133, indicating the cross-species conservation of CD133 activity in invertebrate and vertebrate photoreceptor cells [100]. Similarly, *Drosophila melanogaster* Prominin-like showed a preferential affinity for apical protrusions of wing imaginal disc cells [99].

Altogether, CD133 shows a profound preference for plasma membrane protrusions, the morphology and organization of which are regulated through multiple interactions of CD133 with various protein and lipid interactors (see below) and/or its posttranslational modification. Of note, almost all CD133-knockout mouse models described so far are viable and fertile [23, 54, 84, 90, 91, 177, 201–204] even though CD133 is normally expressed in the male reproductive tract and spermatozoa and may play a role in sperm maturation [10, 13, 202, 205–207]. This indicates potential functional redundancy of CD133 in specific tissues [84, 92], despite a recent study reporting male infertility after deletion of the *Prom1* gene in a particular mouse background [208]. Compromised spermatogenesis has also been reported for some individuals of *Prom-1*-deficient males showing no interference with development or fertility in general [203]. It remains to be determined whether the genetic background and/or other factors, e.g., expression levels of CD133 interactors, can influence the impact of CD133 on the biogenesis and/or maintenance of functional membrane protrusions.

Thus, advancing our basic knowledge of CD133 may help in understanding not only its impact on protruding membrane structures, but also its involvement in various cell signaling pathways and processes, including proliferation/differentiation, autophagy and cell migration.

### CD133 and lipid rafts

The subcellular localization of CD133 in plasma membrane protrusions (e.g., microvilli) relies, at least in part, on its association with a specific membrane microdomain [209, 210]. These submembrane domains, called “lipid rafts”, are rich in specific membrane lipids such as cholesterol and sphingolipids [211]. The integrity of these lipid rafts particularly depends on membrane cholesterol. Lipid rafts play an essential role in cellular processes, including membrane trafficking, epithelial polarity, membrane budding and fission, and signal transduction [212–214]. The implication of lipid rafts in CSC self-renewal, quiescence and EMT, which are mediated by various signaling pathways, has made them putative targets for cancer eradication (reviewed in Ref [215]).

The classical biochemical method used to determine the association of a given protein with lipid rafts is based on protein resistance to extraction with certain nonionic detergents under cold conditions; Triton X-100 is the most commonly used detergent for these assays [216, 217]. Although CD133 was completely soluble after incubation with Triton X-100, cholesterol-dependent detergent resistance of CD133 was observed with other detergents, such as Lubrol WX, Triton X-102, or Brij 58, making these CD133-containing lipid rafts different from others [209, 210]. These biochemical observations were corroborated by morphological data where the specific retention of CD133 in microvillar structures was disrupted after the depletion of membrane cholesterol [209] (reviewed in Ref [218]). A relationship between CD133 and lipid rafts is supported by its direct interaction with membrane cholesterol, as demonstrated using a photoactivatable cholesterol analog [209].

Other lipid species may also interact with CD133, including gangliosides such monosialoganglioside 1 (GM_1_) and disialoganglioside 3 (GD_3_). Taïeb and colleagues have proposed two ganglioside-binding motifs in the EC1 and EC2 of CD133 [219], corroborating the colocalization of CD133 with GM_1_ at the membrane protrusions of epithelial and nonepithelial cells [185, 220, 221]. The importance of the GM_1_-binding motif in CD133 was further dissected by creating point mutations, as one CD133 mutant (named 2 M) produced microvilli or filopodia with altered morphology in MDCK cells and fibroblasts, respectively [177]. Notably, branched microvilli and/or with a "pearling" state were observed (Fig. 3a, see legend). These effects were related to an increased phospho-Y828-dependent interaction of CD133 with either Arp2/3 complex (see above) or PI3K leading to their activation [177]. How does the extracellular domain of CD133 influence the activity of cytosolic proteins, and subsequently the organization of membrane protrusions? The interaction of CD133 with gangliosides in the plasma membrane outer leaflet may determine the phospholipid composition of the inner leaflet (e.g., the phosphatidylinositol 4,5-bisphosphate (PIP_2_)/phosphatidylinositol (3,4,5)-trisphosphate (PIP_3_) ratio), by regulating CD133-driven PI3K activation, which may be responsible for the uncoupling of the microvillar membrane from the underlying cytoskeleton, resulting in irregularly shaped microvilli [177]. Linker proteins that interact with actin filaments, such as myosin and ezrin, are involved in the membrane binding which is regulated by clusters of PIP_2_ [222, 223]. An increase in the PIP_3_ level might also induce the activation of the Arp2/3 complex [224, 225]. By promoting membrane lipid clustering, CD133 may thus mediate direct crosstalk between lipid bilayer leaflets (Fig. 3a). Together with its lipid interactors, CD133 might control the shape and organization of highly curved membranes, such as those found in microvilli, cilia, filopodia and TNTs (see below) [213, 226–228] (reviewed in Ref [218]).

Importantly, cholesterol and gangliosides are not merely structural components of the membrane microdomains affecting membrane structure but, either alone or as a part of lipid rafts, can also modulate signal transduction [214, 229, 230]. Thus, in addition to acting as structural units involved in the architecture of membrane protrusions, CD133 and associated lipid rafts can constitute membrane signaling platforms. The involvement of CD133 as a signaling transduction component in different aspects of cell physiology and tissue regeneration is discussed in subsequent sections.

### CD133: intra- and extra-cellular trafficking

As an organizer of the plasma membrane, especially protrusions, CD133 may be involved in membrane turnover and/or recycling [177]. The proper composition of biological membranes is essential for their physical properties and functionality, as in the case of the plasma membrane, which mediates the activation of signaling pathways [231]. The dynamic movement of CD133 from the plasma membrane to intracellular compartments via endocytosis and/or its release into the extracellular medium in association with membrane particles (see next section) may affect the homeostasis of fully differentiated cells and perhaps the proliferation of cells with stem cell properties. The expression of a ganglioside-binding mutant of CD133, stimulating its ubiquitination and interaction with syntenin-1, coincided with an increase in intracellular multivesicular structures, which highlights the importance of proper interaction between CD133 and certain plasma membrane lipids [177]. CD133 internalization may also indirectly impact various signaling pathways associated with the cell surface, such as those linked to ciliary structures and/or those in other subcellular compartments.

The presence of CD133 in the cytoplasmic compartment, probably in association with the endosomal system, was found to be a high-risk factor for cancer patients survival [232–235]. In blood-derived stem and progenitor cells [81], the intracellular pool of CD133 may contribute to the vasculogenic potential of the cell [68]. The underlying mechanism remains to be established, but the interaction of CD133 with vascular endothelial growth factor, which potentiates its action on angiogenesis, could be part of the answer [236].

**Fig. 4:**
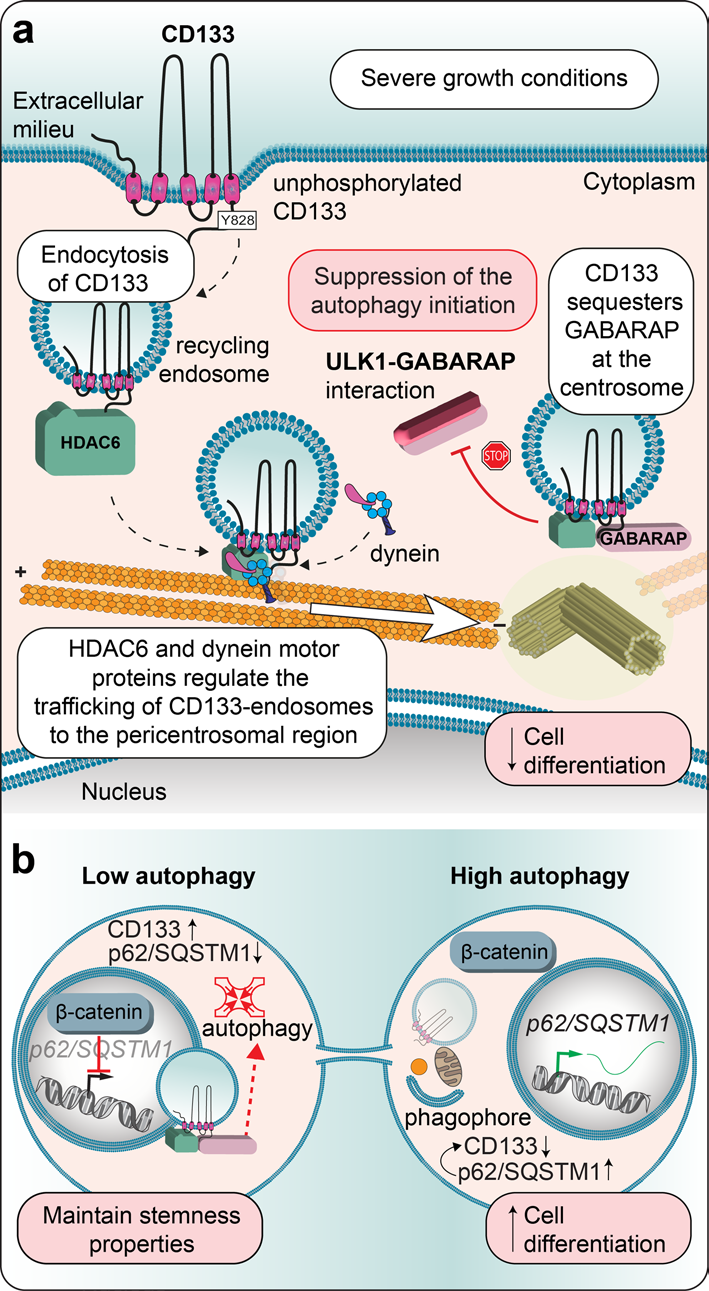
The impact of CD133 on autophagy relies on its phosphorylation status. **a** Under severe growth conditions, the absence of Src-dependent phosphorylation of CD133 Y828 favors its internalization into recycling endosomes. Through its interaction with HDAC6, CD133 is redistributed via motor protein dynein and microtubules to the pericentrosomal region, where its binding to GABARAP prevents autophagy by hindering the GABARAP interaction with ULK1. Suppression of perinuclear CD133-mediated autophagy in cells with stem cell properties favors the maintenance of an undifferentiated state.** b** The asymmetric distribution of CD133 in dividing neuroblastoma cells impacts autophagic activity in nascent cells. During cytokinesis, the presence of CD133 together with GABARAP and HDAC6 in recycling endosomes located asymmetrically in the pericentrosomal region and the nuclear translocation of β-catenin cooperatively suppress the autophagic activity in a nascent daughter cell by inhibiting GABARAP-mediated initiation of autophagy and repressing the expression of SQSTM1, which may also protect CD133 from degradation. Such interplay may promote the maintenance of stem characteristics via a reduction in autophagy, while the absence of CD133 and increased autophagy may favor cell differentiation. Illustrations in panels a and b are adapted from Refs [190] and [247], respectively

Several reports have suggested a positive role for cytoplasmic CD133 in autophagy, which regulates the survival of cancer cells and normal retinal epithelial cells under stress signals, such as those from a nutrient-deprived environment [237–239]. Autophagy is a conserved multistep intracellular process involving autophagosome initiation, elongation and maturation and subsequent fusion with a lysosome, which acts as an indispensable mechanism for removing damaged, denatured, or senescent aggregated proteins and/or organelles. CD133 might participate in autophagosome maturation through its interaction with the autophagy receptor p62/sequestosome 1 (SQSTM1) and HDAC6 [239]. p62/SQSTM1 acts as an adapter molecule that links autophagic cargoes to autophagosomes [240]. These observations may show clinical promise, as targeting CD133-related signaling and autophagy may enhance cancer therapy. Recently, Izumi and colleagues reported an intriguing mechanism affecting the subcellular localization of CD133 and regulating autophagy in colorectal carcinoma and neuroblastoma cell lines [190]. The authors proposed that after endocytosis under severe growth conditions, recycling endosome-associated CD133 is redistributed towards the pericentrosomal region via its interaction with HDAC6 and dynein motor-dependent trafficking along microtubules. Therein, binding of CD133 to the $\upgamma$-aminobutyric type A receptor-associated protein (GABARAP) prevented autophagy by impeding the interaction of GABARAP with Unc-51-like autophagy activating kinase 1 (ULK1), which contributes to the initiation of the autophagy process (Fig. 4a) [241, 242]. Of note, a microtubule-associated protein 1 light chain 3 (LC3)-interacting region (LIR) [243] spanning positions 828–831 in CD133 might mediate the interaction of CD133 with GABARAP [190]. However, this interaction remains to be formally demonstrated. Importantly, the aforementioned process specifically involved unphosphorylated CD133, as the phosphorylated form did not interact with HDAC6 and remained at the plasma membrane [190]. By inhibiting autophagy, pericentrosomal CD133 suppresses cell differentiation and primary cilium formation and allows maintenance of the undifferentiated state [190]. How phosphorylation impedes HDAC6 binding still needs to be elucidated.

The internalization of CD133 and its transport to intralumenal vesicles (precursors of exosomes) found in the MVBs of CD34^+^ HSPCs might determine the fate of these cells, as the partial or complete loss of CD133, either by degradation in the lysosomal system and/or discharge in association with exosomes, is somehow linked to cell differentiation [81] (see below). Remarkably, in the endosomal compartment of CD34^+^ HSPCs, CD133 has been shown to be distributed symmetrically or asymmetrically during cytokinesis, which may support proliferation or differentiation, respectively [27, 244]. Similar features have been observed in cancer cells [245, 246]. As demonstrated in neuroblastoma cells, the asymmetric distribution of pericentrosomal CD133 and nuclear β-catenin cooperatively suppressed autophagic activity in a nascent daughter cell during cytokinesis by inhibiting *p62/SQSTM1* expression (Fig. 4b) [247]. Further investigation is needed to decipher the positive or negative implication of CD133 in the autophagy processes, which might depend on the basal autophagic activity of the cell of interest and/or external cues [247].

In glioblastoma stem cells, the asymmetric redistribution of the CD133 pool and perhaps of the associated lipid rafts during cell division could produce a progeny with coenriched growth factor receptors, which may contribute to the generation of a more drug-resistant CSC population [248]. In neuroepithelial progenitor cells, the asymmetric inheritance of CD133 that occurs during neurogenic cell division relies on the apical localization of CD133 [249]. Together with other constituents in the apical domain, including those associated with membrane protrusions such as microvilli and the primary cilium, CD133 may contribute to the cell fate determination [249]. The spatiotemporal relationship between CD133 and the autophagy machinery in neural progenitors remains to be further studied. Nevertheless, as autophagy has been implicated in differentiation and primary ciliogenesis [250–254], it is tempting to speculate that CD133 mediates the crosstalk between these processes in a phosphorylation-dependent manner. These cellular processes could be involved in fate decision that upon dysregulation could lead to cancer [247].

Surprisingly, CD133 has also been reported to localize to the nucleus of tumor cells derived from rhabdomyosarcoma [255] and other childhood sarcoma subtypes, such as osteosarcoma and Ewing’s sarcoma [256]. Several independent studies confirmed that CD133 may localize to the nucleus of various normal and cancer cell types, including mouse incisor tooth epithelia [182], breast carcinoma [257], non-small cell lung carcinoma [258], melanoma [259] and colorectal carcinoma [260]. Contradictory results have been reported regarding its prognostic significance when it is located in the nuclear compartment of different cancers. Although high nuclear CD133 expression has been correlated with poor outcome in non-small cell lung carcinoma [258], it has been associated with a favorable prognosis in patients with colorectal adenocarcinoma [260]. The role of nuclear CD133 and its transport through the nuclear membrane are still poorly understood, but the aforementioned study by Singer and colleagues provided the first insights into potential mechanisms. In mouse incisor epithelial stem cells, CD133 was found to orchestrate the transition of stem cells towards more differentiated cells via a primary cilia-dependent process in which it associates with Glis2 [182], a transcription factor involved in Sonic Hedgehog signaling, one of the major regulators of stem cell differentiation [261]. The CD133-Glis2 complex is translocated from the primary cilium to the nucleus via an importin β1-mediated cytoplasmic-nuclear transport to induce the expression of Glis2 downstream targets, such as STAT3, a transcription factor implicated in stem cell maintenance and activation [262]. Knockdown of CD133 in mice lowered the expression of Glis2 and vice versa, implying their functional relationship, and moreover, knockdown of either gene resulted in the suppression of STAT3 expression [182]. Of note, molecular crosstalk between CD133 and STAT3 signaling reportedly controlled autophagy [263], further linking ciliogenesis and autophagy, which in turn may regulate stem cell proliferation and differentiation.

Here again, the various subcellular localization and the intracellular transport dynamics of CD133 are not unique to mammals, as they have been observed in fruit flies [102, 264, 265]. For instance, in addition to membrane protrusions, Prominin-like has been shown to be located in mitochondria, where it directly interacts with ND20, a complex I subunit in the respiratory chain [102]. The inhibition of Prominin-like expression increased the levels of reactive oxygen species, reduced cytoplasmic and mitochondrial ATP, and led to total mitochondrial dysfunction. Similarly, Prominin-like has been proposed to be involved in the control of body size in adult flies, as a mutant lacking this protein was larger with excess weight accompanied by higher fat deposits [264]. The accumulation of lipid droplets in fat body cells and decreased mitochondrial β-oxidation rates in whole flies were observed. The impact of CD133 on energy-consuming metabolic processes has been linked to the *Drosophila* homolog of the TOR and insulin-like peptide 6 signaling pathways. A link between CD133 and mTOR signaling has also been reported in mammals, where cytoplasm-located CD133 influenced autophagosome maturation and trafficking (see above) [239]. Another loss-of-function study demonstrated that a Prominin-like mutant exhibited an extended life span and metabolic defects such as an increase in circulating carbohydrate levels, lipid storage, and starvation resistance [265]. These phenotypes were related to glucose metabolism by the control of insulin signaling. In agreement with this physiological impact, Prominin-like expression has been mapped in the adult brain to the pars intercerebralis region containing insulin-producing cells [265]. Prominin-like protein was found to affect the morphological features of primary neural cells [266]. The observations regarding *Drosophila* Prominin-like protein are in agreement with earlier studies proposing a link between CD133 and glucose metabolism in myotubes, as elevated glucose levels increased CD133 expression, while CD133 overexpression promoted glucose uptake [267]. Similarly, CD133 has been shown to be involved in hepatoma cell survival through its regulation of autophagy and glucose uptake [237]. A relationship between CD133 expression and bioenergetic stress affecting mitochondrial functions has also been proposed in the context of glioma [268] (reviewed in Ref [269]).

Altogether, the dynamics of the subcellular localization of CD133 and its relatives, which influence various cellular processes and metabolism, must be considered when studying its function under particular conditions. From a technical point of view, the presence of intracellular (cytoplasmic and/or nuclear) pools of CD133 must be taken into account when analyzing its expression by immunocytochemistry and flow cytometry, particularly in the absence of cell permeabilization, or when CD133 is chosen as a prognostic biomarker or as the cell surface target of a therapeutic strategy [68, 81].

### CD133 and extracellular vesicles

Besides its association with various types of plasma membrane protrusions or specific organelles, CD133 is released into the extracellular milieu, including various bodily fluids, in association with EVs [81, 191]. Although the initially proposed function of EVs was to remove “cell dust” from cells and thus maintain their homeostasis, EVs are now recognized as mediators of intercellular communication in a variety of biological processes, including embryogenesis and immune responses, as well as in cancer progression and metastasis [270–272]. EVs carry specific sets of biological materials (e.g., proteins, lipids and nucleic acids) that often reflect the physiological state of the cells from which they originate [273]. Once released, EVs interact with and/or are potentially internalized by recipient cells, whose characteristics may thus be altered. The release of specific cellular components through EVs may also alter the fate of donor cells.

These nanosized particles are classified into two main categories based on their biogenesis: exosomes (typically approximately 40–100 nm in diameter) and ectosomes/microvesicles (hereafter MVs; 50–1000 nm in diameter) [271]. Exosomes are of endosomal origin, as they are formed by the inward budding of endosome-limiting membranes, leading to the formation of MVBs that subsequently fuse with the plasma membrane and discharge their small intralumenal vesicles into the extracellular medium [271, 274]. In contrast, MVs are derived directly from the plasma membrane as they often bud or shed from protruding membranes (reviewed in Refs [275–277]). Depending on the cell type, CD133 has been associated with exosomes or MVs [81, 191]. However, CD133 may be associated with both entities in a given biological fluid, reflecting the different cellular sources in contact with these fluids and/or the release by both mechanisms from a particular cell under specific conditions or in diseases such as cancer.

**Fig. 5:**
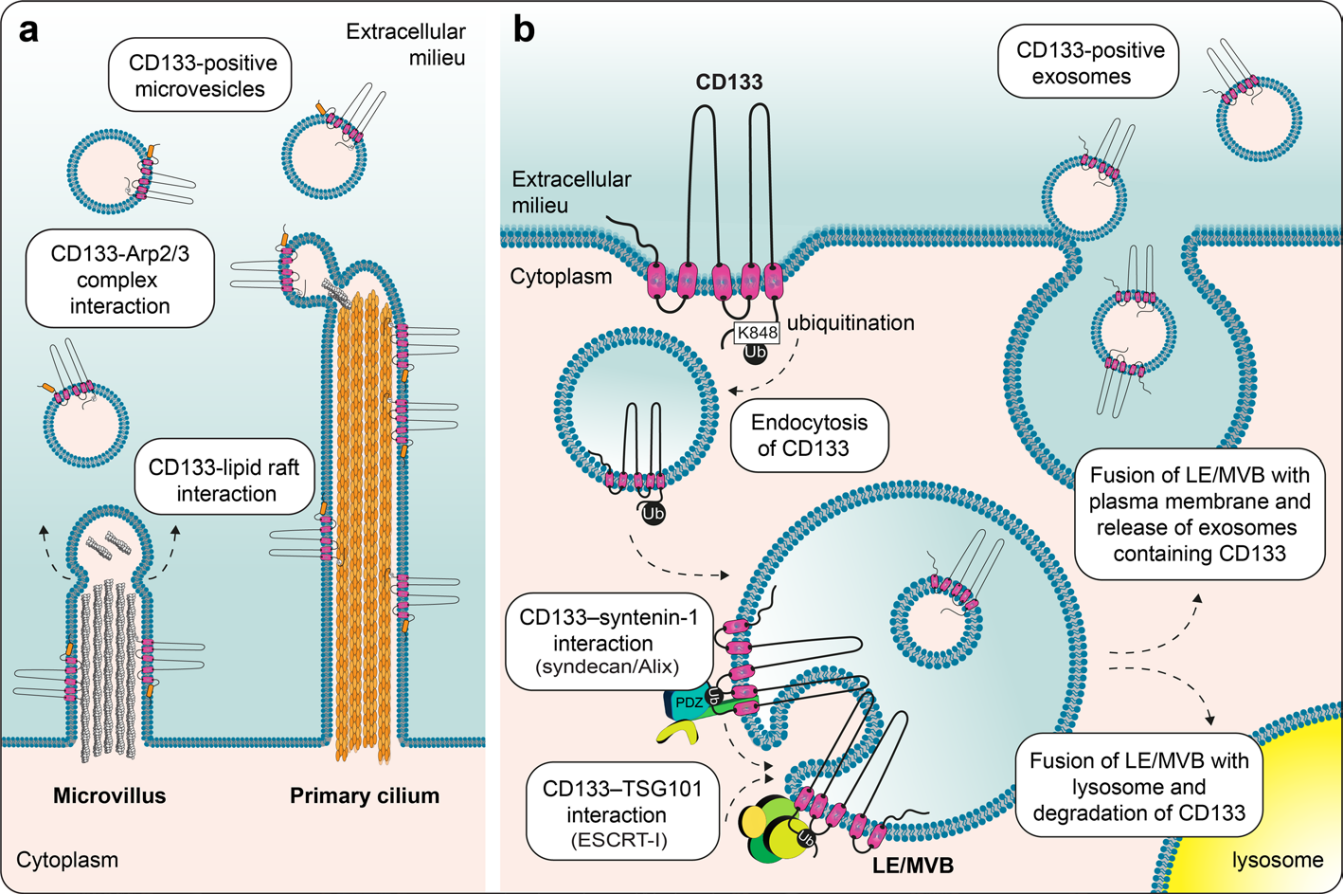
Release of CD133 into the extracellular environment in association with microvesicles and/or exosomes. **a** CD133 is extracellularly released in association with MVs after budding from microvilli or the primary cilium. The association of CD133 with cholesterol-rich lipid rafts and/or its interaction with Arp2/3 complexes, among other interactors, might favor the formation and budding of MVs. **b** CD133 is released in association with exosomes after the fusion of the late endosome/multivesicular body (LE/MVB) with the plasma membrane. Alternatively, LE/MVB can fuse with lysosomes, resulting in the CD133 degradation. CD133 endocytosis and sorting into intralumenal vesicles within the LE/MVB may be promoted via its ubiquitination at lysine (K) 848 and its interactions with TSG101 and/or syntenin-1 that are involved with Alix, syndecan, and the ESCRT machinery in the exosome biogenesis. Illustrations in panels a and b are based on data presented in Refs [177, 182, 278] and [24, 81, 165], respectively

In 2005, Marzesco and colleagues were the first to report the release of CD133 into the external environment; they described its release both in physiological fluids, notably cerebrospinal fluid and urine, and in conditioned medium of a cancer cell line in culture [191]. They showed that CD133^+^ MVs were released from neuroepithelial progenitor cells and that their appearance in the extracellular milieu coincided with a reduction in CD133^+^ microvilli, which were most likely the origins of the MVs (Fig. 5a). Consistent with this hypothesis, the CD133^+^ MVs did not contain the bona fide exosome marker CD63 [191]. The budding of CD133^+^ MVs from microvillar structures depends on specific cholesterol-rich lipid rafts, suggesting that the interaction of CD133 with membrane cholesterol is the main driver of MV release [278, 279]. Indeed, a reduction in membrane cholesterol increases the release of CD133^+^ MVs from microvilli [278], while mutations in the GM_1_-binding domain of CD133 reduce their release [177]. The GM_1_-binding-dependent interaction of CD133 with the Arp2/3 complex may also be relevant, as the Arp2/3 complex has recently been shown to be involved in MV release [280, 281]. The primary cilium and midbody, i.e., a transient structure that connects two nascent daughter cells at the end of cytokinesis, are other sources of CD133^+^ MVs (Fig. 5a) [192]. The midbody itself can be released and thus constitutes a large and particular CD133^+^ EV [191, 192, 282]. Overall, the interest in CD133 as a microvillar lipid-binding membrane protein, in parallel with research on cytoskeletal regulators [283, 284], has led to more studies into MV shedding from plasma membrane protrusions [276]. For example, it has recently been shown that the fruit fly Prominin-like is important for both the integrity of microvilli and the release of MVs, contributing to the proper morphogenesis of wing imaginal discs through long-distance signaling of the Hedgehog morphogen [285].

In addition to MVs, as mentioned above, CD133 is associated with exosomes, as demonstrated in primary human CD34^+^ HSPCs (Fig. 5b) [81]. CD133 sorting into exosomes may be related to its ubiquitination and interactions with TSG101 and/or syntenin-1 (see above). Whether lipid rafts and/or certain gangliosides are involved in these processes remains to be determined. It is noteworthy that in addition to GM_1_, CD133 bears a potential GD_3_-binding site in the EC2 domain near its second transmembrane domain [219], possibly contributing to its incorporation into intralumenal vesicles of MVBs, as the GD_3_ ganglioside has been reported in exosomes [286].

Although the function(s) of CD133^+^ EVs is poorly documented, a correlation between their release, irrespective of the mechanism, and the onset of the cell differentiation have been reported in three distinct cellular systems: murine neural progenitors, human Caco-2 cells and primary human CD34^+^ HSPCs [81, 191]. For example, the release of CD133^+^ MVs from neuroepithelial progenitors occurs at the very beginning and early phase of neurogenesis, resulting in apical membrane remodeling with loss of microvilli, promoting cell differentiation [191] (reviewed in Refs [218, 287]). In epithelial Caco-2 cells, which in confluent culture show spontaneous differentiation leading to mature colonic epithelial cells, the release of CD133^+^ MVs coincides perfectly with the differentiation process [191]. Similarly, the differentiation of CD34^+^ HSPCs in culture has also been associated with the release of CD133^+^CD34^–^ exosomes, a phenomenon that can be stimulated by phorbol esters such as phorbol 12-myristate 13-acetate, further linking HSPC differentiation and CD133 release [81].

Interestingly, CD133^+^ EVs contain all the characteristics of lipid rafts (reviewed in Ref. [279]), including the binding of CD133 to membrane cholesterol [81, 278, 288, 289]. As initially proposed by Marzesco and colleagues, these nano(micro)membrane entities may contain the determinants and/or some components of certain signaling pathways necessary for the maintenance of stem (cancer stem) cell properties [191]. The loss of lipid rafts via the release of CD133^+^ EVs may promote cell differentiation. This concept of “*stem cell-specific lipid rafts*” is attractive in the context of stem cell-based tissue regeneration and CSCs. Interfering with CD133^+^ EV release that promotes differentiation may thus favor cell proliferation [191] (reviewed in Ref [27]). In support of this hypothesis, blocking MVB maturation with ammonium chloride impeded both sodium butyrate-induced differentiation and CD133 depletion in two colon cancer cell lines, suggesting that the release of CD133^+^ EVs is essential for cell differentiation [290]. With the asymmetric distribution of CD133 during cell division (see above), the release of CD133^+^ EVs may act as a complementary process for the expulsion of CD133-related signaling components and/or its associated lipid rafts [191].

The release of CD133^+^ EVs may not only constitute a clearance process that leads to cell differentiation but may also produce the vehicles necessary for intercellular communication, delivering information and signaling factors to surrounding tissues (reviewed in Ref [275]). The uptake of CD133^+^ EVs by stem cells or cancer cells has been demonstrated in several studies [81, 288, 290]. For instance, metastatic melanoma FEMX-I cells released CD133^+^ EVs carrying proteins and microRNAs, which promoted tumorigenic/prometastatic activity in recipient cells. Thus, the transfer of CD133^+^ EVs to bone marrow-derived mesenchymal stem cells significantly increased their invasive capacity in vitro [288, 289]. Similarly, CD133^+^ EVs released by HT29 colon cancer cells increased the proliferation and motility of both colorectal cancer cells and normal fibroblasts [290]. These effects were coupled with an increase in phosphorylation of Src proteins and extracellular signal-regulated kinases as well as in the expression of genes associated with EMT. CD133^+^ EVs, such as those released from Kirsten rat sarcoma virus oncogene homolog (*KRAS*) mutant colon cancer cells, can also be involved in oncoprotein trafficking [291]. The small GTPase KRAS is a well-characterized oncoprotein that increases the malignancy and metastatic potential of cancer cells by acting as an epidermal growth factor receptor (EGFR) signaling transducer [292, 293]. Specifically, Kang and colleagues reported that the transfer of KRAS mutants via CD133^+^ EVs (in this case, MVs) to surrounding nontumorigenic cells activated downstream KRAS signaling, leading to an increased cell motility, proliferation and resistance to anti-EGFR drugs [291]. Interestingly, the amount and sizes of budding MVs depended on the level of CD133 expression, which stimulated and inhibited the activities of the small GTPases RhoA and Rac1, respectively [291].

CD133^+^ EVs in various bodily fluids, notably cerebrospinal fluid, urine and seminal fluid, may have, in addition to their biological and physiological impact, clinical value as noninvasive biological tools to monitor disease progression or tissue regeneration after organ transplantation [191]. For example, Huttner and colleagues demonstrated in a series of publications that CD133^+^ EVs associated with cerebrospinal fluid can be used as a biomarker to monitor neural diseases such as cancer or brain injury [294–297]. The expression of CD133 in various renal cell types (e.g., cells in proximal tubules and parietal layer of Bowman’s capsule of juxtamedullary nephrons) [1, 11, 12, 43, 45] and derived urinary EVs that mirror in some way the tissue expression profile may also be useful for monitoring kidney disease and tissue recovery after kidney transplantation [298–300] (reviewed in Ref [301]). In all cases, the potential use of CD133^+^ EVs as bodily fluid-associated biomarkers requires further assessment, particularly with a large cohort of patients.

### CD133 and tunneling nanotubes

In the context of intercellular communication and signaling, the exchange of CD133 between cells can be mediated via TNTs, which connect adjacent cells over a short or long distance. Discovered by Gerdes and colleagues, TNTs are thin, straight and long protruding membrane structures that are not in direct contact with the extracellular matrix, in contrast to other protruding structures such as filopodia [302]. Most TNTs are composed of microfilaments (F-actin), although tubulin has been detected in some TNTs [302–304]. They are categorized based on the junctional connections between cells as closed-ended or open-ended, with the latter type of TNTs leading to cytoplasmic continuity between interconnected cells [305]. Open-ended TNTs have been implicated in the transport of diverse cellular components, including cytoplasmic molecules and organelles (e.g., mitochondria), whereas closed-ended TNTs have been reported to mediate the transport of electrical impulses between cells [303, 306, 307].

**Fig. 6:**
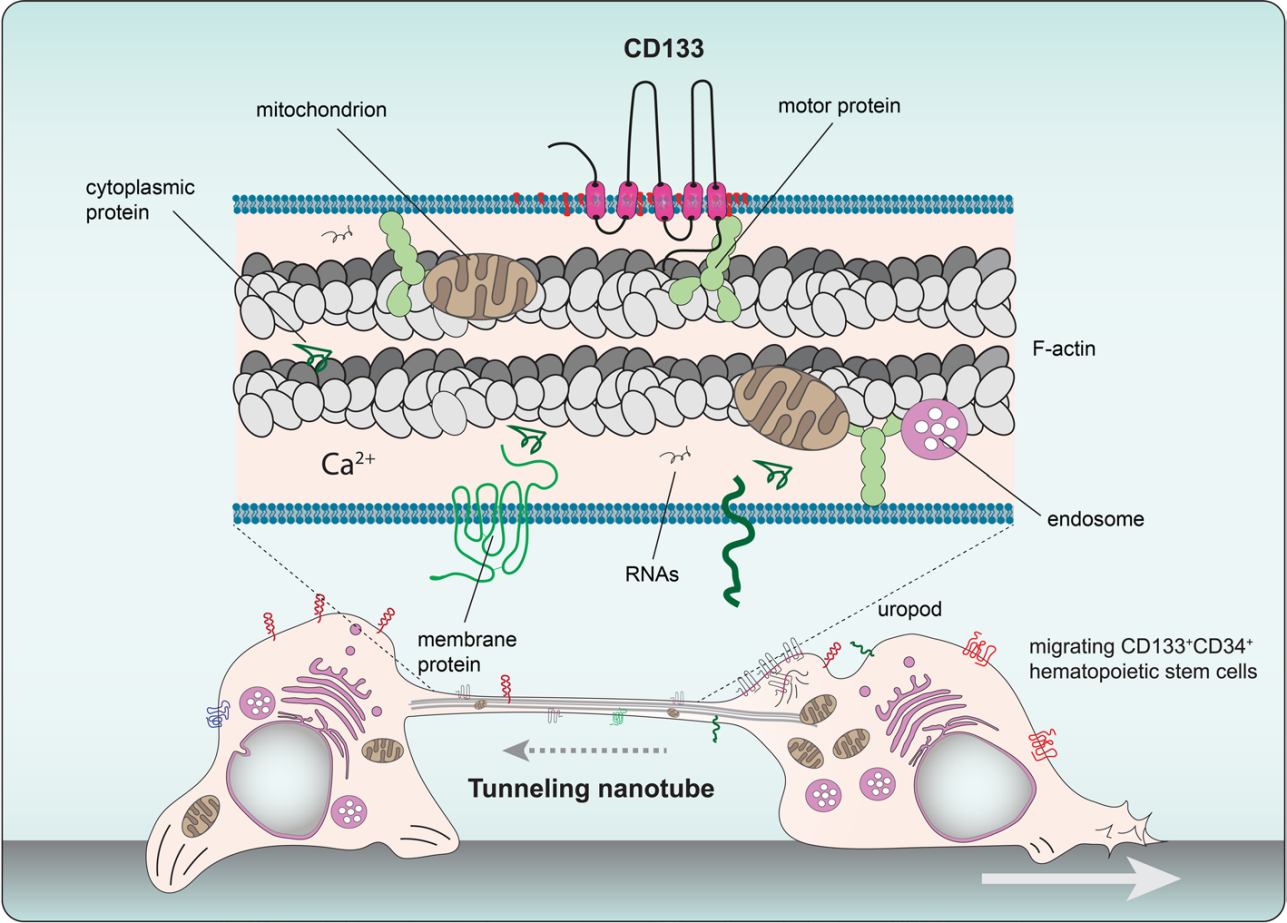
Tunneling nanotubes mediate the transfer of CD133 between hematopoietic stem and progenitor cells. CD133 is exchanged between CD133^+^CD34^+^HSPCs (or KG1a hematopoietic leukemic cells) via TNTs. In various cellular systems, these transient narrow actin-based tubular connections have been reported to mediate the transfer of organelles, soluble and membrane proteins and nucleic acids or to contribute to calcium signaling, thereby promoting intercellular communication between adjacent or distant cells. In cells of hematopoietic origin, TNT biogenesis depends on cell polarization and occurs during cell migration (solid arrow) with one of two cells in direct contact forming membrane extensions from the uropod membrane at its rear pole, where CD133 is concentrated. Mechanistically, the biological properties of CD133, including its direct interaction with membrane cholesterol (red lipid) and its incorporation into cholesterol-rich membrane microdomains, may modulate the lipid composition and the local organization of the plasma membrane in TNTs. The CD133 interaction with PI3K may lead to the conversion of the docking PIP_2_ into PIP_3_ at the inner leaflet of CD133-containing membrane microdomains, thereby regulating their interaction with the underlying actin cytoskeleton. The binding of the actin motor protein myosin to PIP_3_ clusters may promote the selective movement of such CD133-containing membrane microdomains along the actin filament and mediate their transfer between TNT-connected cells (dashed arrow). Illustration is adapted from Ref [308]

One of our laboratories reported that closed-ended TNTs were involved in the selective intercellular transport of certain membrane proteins, such as CD133, between primary human CD34^+^ HSPCs and KG1a hematopoietic leukemia cells [308]. The association of CD133 with lipid rafts may explain its selective and directional transport along the surface of TNTs in small clusters, similar to cytoplasmic phospho-myosin light chain 2, suggesting that this actin motor protein might be implicated in CD133 transport along TNTs (Fig. 6, see the corresponding legend for the potential mechanism of CD133 transfer) [308]. Accumulation of CD133 occurs at the junctional complex before its transfer from donor to acceptor cells, but the mechanism underlying this transfer of membrane remains to be determined [308]. Interestingly, CD34^+^CD133^+^ HSPCs were more likely to generate TNTs than their CD34^+^CD133^–^ counterparts, suggesting that more primitive stem cells deploy this means of communication to exchange or share materials among themselves [308]. Differentiation may be triggered in donor HSPCs by the reduction in CD133 level and/or that of its associated lipid rafts, while their increase in recipient cells may promote proliferation, thus contributing to the replenishment of the bone marrow stem cell niche and the formation of new mature blood cells [308]. Nonetheless, the impact of CD133 transfer on recipient cells, the composition of the associated lipid rafts that may harbor specific components of signaling pathways and whether CD133 directly contributes to TNT formation still need to be answered by further studies.

Collectively, these molecular and cell biological findings underscore the importance for future studies of examining the subcellular localization of CD133 (or its orthologs across species) and the posttranslational modifications that may influence it. Elucidation of the regulatory factors may be essential to determine the function and/or involvement of CD133 in membrane organization and dynamics, which, in addition to its structural and physical properties, may impact various signaling networks.

## CD133 and signaling pathways

In recent years, the involvement of CD133 in various signaling pathways has been postulated. As no study has reported any soluble ligand that could demonstrates a functional role for CD133 as a membrane receptor, it is important to determine its interactors, especially those directly involved in signaling pathways [33, 309, 310]. In this section, we highlight the pathways in which CD133 has been shown to play a role or exert an influence, particularly in cancer cells.

### CD133 and RhoA/ROCK signaling influence cell morphology

Rho GTPases orchestrate various biological processes including cell cycle progression, vesicular transport pathways, cell migration and cytoskeleton dynamics [311]. Among them, RhoA and its main downstream effectors, Rho-associated coiled-coil-containing protein kinase (ROCK) 1 and 2, are key players in the regulation of cytoskeletal remodeling and cell polarity, acting on actin, intermediate filaments and microtubules [312–314].

In addition to the link between RhoA and CD133 in the formation of MVs mentioned above, it has been reported that overexpression of CD133 in retinal pigmented epithelium cells or mouse embryonic fibroblasts leads to the formation of multiple RhoA-dependent long membrane extensions (named fibres by the authors) oriented in an opposite direction to that of cell movement [178]. These fibres, although somewhat similar to the retraction fibers left behind by migrating cells, were surprisingly formed independently of F-actin or α-tubulin polymerization and, consistent with the lipid-binding properties of CD133, were highly enriched in membrane cholesterol. Interestingly, five critical residues (KLAKY818) mapped to the end of the last transmembrane domain (TM5) of CD133 were found to be essential for the formation of these fibres [178]. In contrast, the phosphorylation of the tyrosine at site 818 (or 828 in the case of CD133.s2) was not required [178], suggesting that these membrane structures are generated independently of the PI3K activity, making them different from the other protruding structures involving PI3K and the Arp2/3 complex, described in the previous section [177]. Of note, KLAKY818 residues in CD133 are in a motif similar to the linear Cholesterol Recognition/interaction Amino acid Consensus sequence (CRAC) domain (L/V-X_1-5_-Y-X_1-5_-K/R) [315, 316]. However, whether they act as cholesterol-binding sites on the cytoplasmic leaflet of the plasma membrane is unknown, and further analysis is required. Interestingly, CD133 appeared to colocalize with active RhoA at sites of fibre formation initialization, and silencing of ROCK1/2 disrupted CD133-induced fibre formation, suggesting that the RhoA/ROCK pathway mediated the biogenesis of these cellular extensions [178]. It remains to be determined how CD133 and Rho activation act synergistically, what the target of ROCK1/2 is in the biogenesis of these CD133-dependent membrane extensions and whether these fibres contain other types of cytoskeletal elements, such as intermediate filaments [178]. Despite the questions, these observations are consistent with the impact of CD133 on the architecture of membrane protrusions and cell migration and are in line with the previous findings that in migrating CD34^+^ HSPCs, CD133 was selectively concentrated in the uropod at the posterior pole, the latter structure being regulated by the RhoA/ROCK1 signaling pathway [317, 318].

The same study by Hori and colleagues demonstrated that fibre formation is induced by the overexpression of Tweety homolog (TTYH) 1/2 proteins, similar to CD133, suggesting that both types of molecules may show functional similarity [178]. TTYH1/2 were reported to act as anion channels that were activated either by calcium ions or cell swelling [319]. Recent structural studies on TTYH based on cryo-electron microscopy combined with functional data refuted their potential functions as the pore-forming subunits of ion channels, although they may act as accessory molecules to these channels [320, 321]. Instead, the hypothesis that these proteins might play a role in the dynamics of membrane lipids was proposed [320]. More interestingly, TTYH1/2 proteins are structurally similar to CD133, including their membrane topology and dimer/tetramer formation [210, 322], suggesting that they may all regulate membrane organization leading to fibre formation. Whether this process is directly related to the chloride efflux activity mediated by TTYH1/2 (or CD133) or to their interaction with other molecules remains to be demonstrated [178].

### Signaling via phosphorylated CD133

The regulation of protein phosphorylation involves specific protein tyrosine phosphatases. Two studies indicated that protein tyrosine phosphatase κ (PTPRκ) was involved in the dephosphorylation of CD133 IC3 [323, 324]. PTPRκ is a member of the group of transmembrane receptors in the classical tyrosine phosphatases family [325]. Its two catalytic intracellular domains dephosphorylate target proteins, and thus regulate intercellular adhesion and cell proliferation [325, 326]. Both catalytic domains of PTPRκ interact with the CD133 C-terminal domain, independently of the phosphorylation status, resulting in the dephosphorylation of residues Y828 and Y852, thereby inhibiting the ability of CD133 to activate two major signaling pathways, namely, the PI3K-Rac-alpha serine/threonine-protein kinase (Akt) and Src-FAK pathways [323, 324]. The importance of the phosphorylation of CD133 in the regulation of cell signaling was strengthened by the discovery of a novel small compound (LDN193189, a derivative of Dorsomorphin) that binds to the IC3 and prevents its phosphorylation [327]. As a result, the interaction of CD133 with PI3K was effectively abolished, leading to the inhibition of Akt signaling and decreased self-renewal and tumorigenicity in liver tumor-initiating cells [327].

#### Tyrosine 828 phosphorylation of CD133 regulates PI3K-Akt signaling

Increasing evidence suggests a role for CD133 as an upstream activator of the PI3K/Akt pathway [163, 328]. PI3K, a heterodimeric protein composed of a catalytic (p110) and a regulatory (p85) subunit (reviewed in Ref [329]), affects several cellular processes, including cell proliferation, apoptosis, and growth and cytoskeleton remodeling [330, 331]. The main downstream molecule of the PI3K pathway is the serine/threonine kinase Akt (also known as protein kinase B), which stimulates the proliferation and survival of stem cells and CSCs [332, 333]. Indeed, upregulated PI3K-Akt signaling is common in a wide spectrum of tumors [334–339], and it is indispensable for the increased self-renewal and tumorigenicity of CD133^+^ cancer cells [163].

**Fig. 7:**
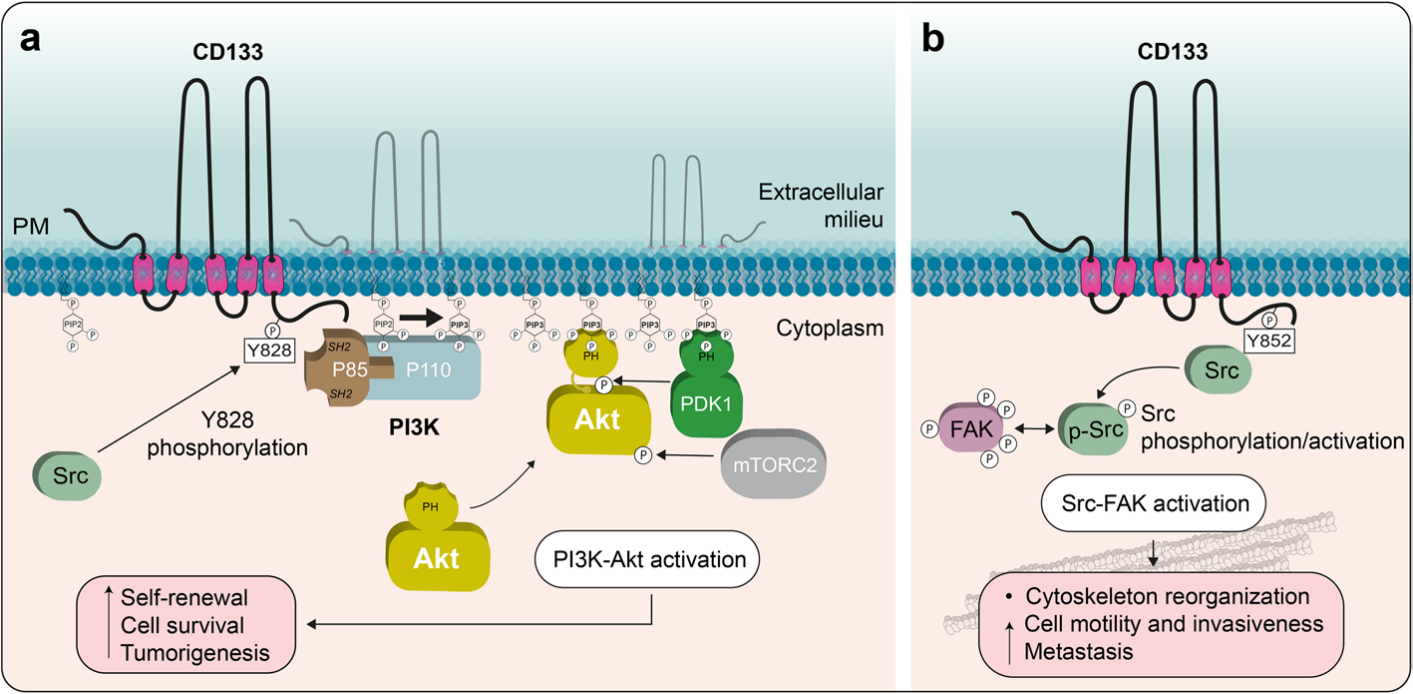
Phosphorylated CD133 regulates the PI3K-Akt and Src-FAK signaling pathways. **a** The Src-dependent phosphorylated tyrosine 828 in the CD133 IC3 binds to the PI3K regulatory subunit p85 via the SH2 domain in the latter, resulting in the translocation of the kinase to the plasma membrane and the phosphorylation of PIP_2_ to yield PIP_3_. Accumulation of the PIP_3_ enables Akt to interact via its pleckstrin homology (PH) domain with the plasma membrane (PM), resulting in a conformational change in the Akt kinase domain, which allows the phosphorylation of a critical residue required for Akt kinase activity by the 3-phosphoinositide-dependent protein kinase 1 (PDK1). The mammalian TOR complex 2 (mTORC2) also phosphorylates Akt, promoting its kinase activity. It should be noted that PDK1 binding to PIP_3_ is not essential for its activity, in contrast to the dependence of Akt on PIP_3_. Then, the resulting activation of the PI3K/Akt pathway promotes self-renewal, cell survival and tumor formation. **b** The phosphorylated tyrosine 852 residue of CD133 directly interacts with Src and mediates its activation. The phosphorylated (p)-Src protein phosphorylates, and then forms a complex with, the FAK protein, triggering EMT-related events and cytoskeletal reorganization. This leads to increased cell motility and invasiveness, among other processes. Inhibition of Src activity by PP2, a known Src activity inhibitor, blocks the activation of FAK phosphorylation and cell migration induced by CD133 (not shown). Illustration in panel a is adapted from Ref [163], while in panel b is based on data from Ref [352]

Mechanistically, as demonstrated in glioma cells, Src-dependent phosphorylation of CD133 at cytoplasmic residue Y828 promotes its interaction with the PI3K p85 subunit, leading to the translocation of PI3K to the plasma membrane and the initiation of Akt signaling (Fig. 7a), which may promote self-renewal, cell survival and tumorigenicity [163]. Manoranjan and colleagues reported an association between overexpressed CD133 and elevated levels of phosphorylated Akt and Wnt in glioblastoma cell lines [340]. Phosphorylated Akt, which inhibits glycogen synthase kinase-3 activity through its phosphorylation at serine 9 [341], may lead to stabilization of β-catenin and thus mediate CD133-Akt-Wnt signaling axis activity, resulting in increased proliferation and self-renewal potential of CD133^+^ cells [340]. Thus, as a putative cell surface receptor, CD133 may mediate Akt-dependent activation of Wnt signaling, which may drive glioblastoma tumor-initiating cells in the brain [340]. The CD133-dependent interplay between pathways might explain the correlation of CD133 with progression and recurrence of brain cancer and poor survival for patients [31, 309, 342].

In thyroid cancer, the activation of Src kinase is facilitated by the close proximity of CD133^+^ cancer cells to acetylcholine-secreting neurons [343]. The released acetylcholine binds to the M3R acetylcholine receptor of thyroid cancer cells, which induces the activation of Src through the phosphorylation at Y416. The latter promotes Y828 phosphorylation of CD133 and activation of the PI3K/Akt pathway, leading to increased resistance of thyroid cancer cells to cytotoxic CD8^+^ T cells [343]. In melanoma, the same phosphorylation of CD133 conferred chemoresistance to an alkylating agent, namely, fotemustine, via the activation of both the PI3K/Akt/mitogen-activated protein kinase-1 and PI3K/mouse double minute 2 pathways [344].

#### Tyrosine 852 phosphorylation of CD133 activates Src-FAK signaling

EMT is a reversible shift in the epithelial phenotype of a cell toward the mesenchymal phenotype, allowing migration of originally adherent cells [345]. Phosphorylated Src kinase (p-Src) controls the onset of EMT in many tumors [346, 347]. Active Src signaling leads to the disintegration of cell‒cell adhesion; promotes cell invasiveness, motility, and proliferation; induces the reorganization of the cytoskeleton; and affects the tumor microenvironment [348].

A link between Src signaling and CD133 has been suggested in the head and neck squamous cell carcinoma (HNSCC). CD133^+^ HNSCC cells exhibited higher levels of p-Src and concurrently displayed properties of mesenchymal cells, such as lower expression of E-cadherin and higher expression of vimentin, fibronectin, and transcription factors OCT4 and NANOG [164]. Moreover, the suppression of *PROM1* transcription downregulated p-Src and favored the acquisition the epithelial phenotype associated with E-cadherin re-expression and OCT4 and NANOG depletion in HNSCC cells [164]. Yet, this phenotype switching did not seem to involve the Src SH2-binding motif, as the expression of the CD133 Y828F mutant did not impair Src activation. The authors therefore proposed that other tyrosine residues in CD133, including Y852, may be the main sites of regulation of Src activity.

One of the p-Src downstream molecules is FAK, a cytoplasmic tyrosine kinase involved in integrin signaling [349, 350]. Once activated, FAK and Src form a Src-FAK complex, which facilitates actin remodeling and cell motility and hence promotes the invasiveness of cancer cells [346, 348, 351]. In this context, CD133^+^ cells of the SW620 colorectal carcinoma cell line exhibited high levels of phosphorylated FAK and Src that were decreased after the knockdown of CD133 expression [352]. This relationship was further supported by the demonstration that CD133 phosphorylated on tyrosine Y852 interacted with Src, leading to Src activation and subsequent formation of a Src-FAK complex which stimulates the invasive behavior of these cancer cells (Fig. 7b) [352]. Altogether, the presence of several tyrosine residues in the IC3 of CD133, with two of them being encoded by a facultative exon, suggest that signaling cascades could be differentially mediated depending on the cellular context.

### CD133, HDAC6, and Wnt/β-catenin signaling

Wnt proteins (the name of which was derived from Drosophila Wingless and mouse Int proteins [353]) are signaling molecules that orchestrate tissue development [354, 355] by regulating the expression of target genes as well as by modifying the cytoskeleton or the mitotic spindle [356, 357]. Activation of the Wnt pathway stabilizes β-catenin, a canonical Wnt downstream molecule, which is then translocated from the cytoplasm to the nucleus, where it forms a complex with members of the TCF family of transcription factors and initiates the transcription of β-catenin-TCF-dependent genes [355].

Wnt signaling has been implicated in the early phase of human hair follicle morphogenesis [358]. During this phase, CD133 expression in a subset of invaginating placode cells was associated with Wnt activation [359]. In early placodes, CD133 was detected in adherens junctions rich in E-cadherin and β-catenin, while in later phases, its expression was spatially and mechanistically correlated with a reduction in membrane β-catenin and E-cadherin levels, a crucial process for proper adherens junction disassembly, suggesting a functional role for CD133 in placode remodeling [359]. The link between CD133 and E-cadherin was supported by Brossa and colleagues, who provided evidence showing that CD133 directly bound E-cadherin and β-catenin to form a complex restraining the β-catenin degradation [360]. Stabilized β-catenin in turn activated a regeneration program by initiating the transcription of Wnt pathway-responsive genes in cisplatin-damaged kidney tubular cells [360]. In the same line, a Glis3/CD133/Wnt signaling axis implicated in the maintenance of the self-renewing capacity of these cells, was uncovered in mouse pancreatic colony-forming units [361]. In general, CD133 seems to be an important upstream regulator of the Wnt signaling pathway in various normal tissues, which in turn may promote CD133 expression via β-catenin-TCF/LEF complex-binding sites present in the *PROM1* gene.

Indeed, the activity of the Wnt pathway is most likely also modulated by CD133 in tumor cells [33, 309, 310]. While the silencing of CD133 expression leads to a suppression of the Wnt pathway, inhibition of Wnt signaling results in the downregulation of CD133 expression [289, 362]. In a metastatic melanoma cell line, the downregulation of CD133 expression mediated by short hairpin RNA was associated with an upregulation of Wnt pathway inhibitors (e.g., Dickkopf-related protein 1 and Dishevelled binding antagonist of β-catenin 1) [363]. Similarly, CD133-depleted metastatic melanoma and ovarian carcinoma cell lines displayed low basal Wnt signaling and a very limited nuclear localization of β-catenin, which was restored after supplementation with the exogenous ligand Wnt3a [289, 362].

**Fig. 8:**
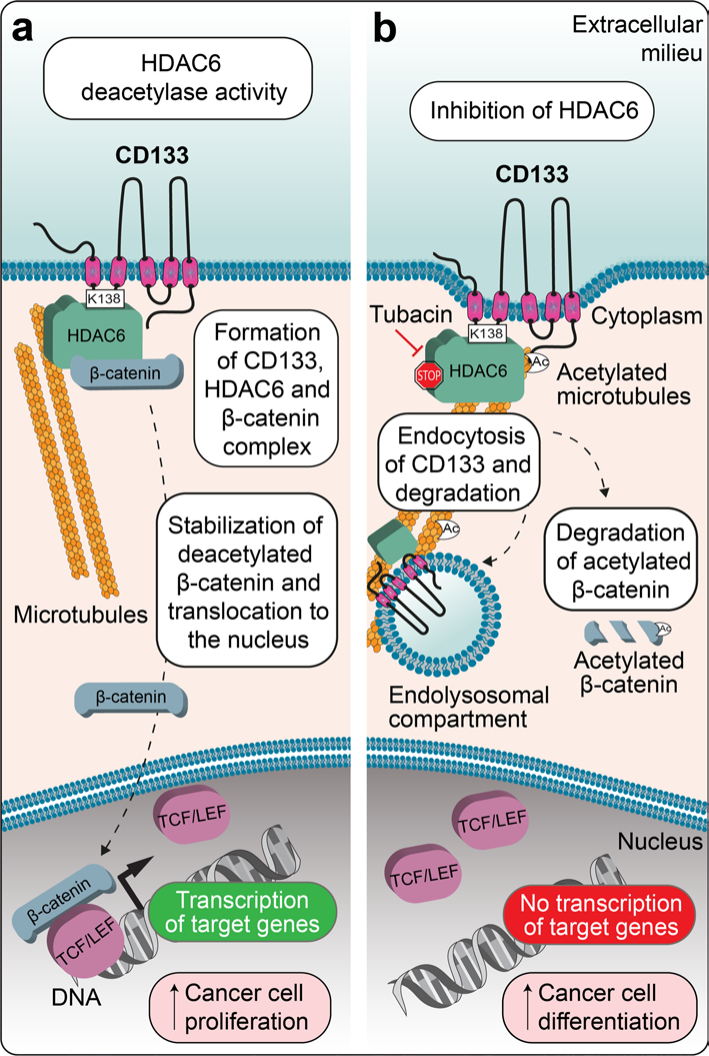
CD133 regulates β-catenin signaling via its interaction with HDAC6. **a** CD133 regulates the formation of a tripartite complex involving HDAC6 and β-catenin, leading to the stabilization of the latter, which may then translocate to the nuclear compartment, where it activates the expression of genes, notably those associated with the Wnt/β-catenin pathway via its interaction with the TCF/LEF transcription factor. The interaction between CD133 and HDAC6 is mediated by IC1 and potentially lysine (K) 138 (numbered according to the splice variant s2). **b** Treatment of cells with tubacin, a specific inhibitor of HDAC6 deacetylase activity, leads to the degradation of acetylated β-catenin (Ac) and thus the impairment of transcriptional activity, while CD133 is endocytosed and degraded upon its transport to the endolysosomal compartment. Therefore, CD133/HDAC6/β-catenin interactions will have an impact on cancer cell proliferation and differentiation. Illustrations in panels a and b are adapted from Ref [362]

As mentioned above, CD133 interacts with another modulator of Wnt signaling, HDAC6 [190, 359, 362], a cytoplasmic histone deacetylase involved in the regulation of β-catenin stability and microtubular remodeling [364, 365]. Besides its interactions with acetylated microtubules, polyubiquitinated misfolded proteins and dynein motors [364, 366, 367], HDAC6 binds via its second catalytic domain to the CD133 IC1, and thus stabilizes CD133 and prevents its degradation in the endosomal-lysosomal pathway [190, 362]. Indeed, CD133 creates a ternary complex with HDAC6 and β-catenin at the plasma membrane that protects the HDAC6 activity, thereby reducing β-catenin acetylation and degradation [362]. This action favors the translocation of β-catenin to the nuclear compartment, and subsequently influence the gene regulation (Fig. 8a) [362]. This process depends on HDAC6 deacetylase activity, as treatment with tubacin, a specific inhibitor of HDAC6 deacetylase activity [368], led to the degradation of acetylated β-catenin (Fig. 8b). Mechanistically, given that the phosphorylation of HDAC6 has been associated with a decrease of its deacetylase activity [369] and that phospho-mimicking mutants of HDAC6 failed to interact with CD133, it has been suggested that the interaction of HDAC6 with CD133 prevents the loss of HDAC6 activity by impeding HDAC6 phosphorylation, thereby increasing its β-catenin-stabilizing and nuclear transfer effects, essential steps to induce the expression of Wnt/β-catenin target genes [362].

### CD133 and TGF-β/Smad2 signaling

**Fig. 9:**
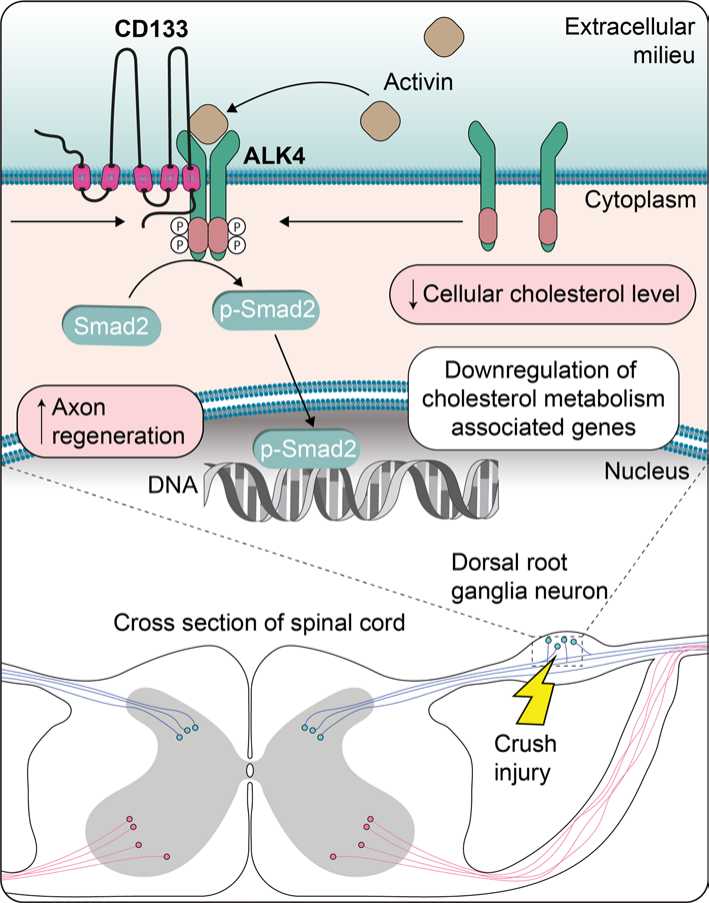
CD133 regulates cholesterol metabolism and peripheral axon regeneration through TGF-β/Smad2 signaling. Mouse dorsal root ganglion crush injury induces the formation of functional ALK4, a TGF-β type I receptor that binds to its ligand activin, a TGF-β superfamily member and a determinant of axon regenerative capacity. At the plasma membrane, the interaction of ALK4 with CD133 stimulates the phosphorylation of Smad2, which inhibits, after translocation into the nuclear compartment, the expression of genes involved in cholesterol metabolism, thereby promoting a positive effect on axon regeneration. Illustration is based on data presented in Ref [371]

The TGF-β family of cytokines comprises more than thirty secreted proteins that are highly conserved among a broad group of organisms [370]. By binding to specific receptors on the cell membrane, TGF-β cytokines regulate diverse cellular processes from proliferation, adhesion, differentiation, and metabolism to cell death. TGF-β receptors act as heterodimeric serine/threonine protein kinases, which phosphorylate the C-terminal domain of Smad proteins. Once phosphorylated, Smad proteins form a heterocomplex that is translocated to the nucleus and activates the transcription of target genes [370]. Aside from the implication of TGF-β in the regulation of CD133 expression in cancer (see above), an intriguing relationship between CD133 and TGF-β/Smad signaling has been observed in a study focused on peripheral axon regeneration after crush injury [371]. CD133 is expressed on dorsal root ganglia neurons and is developmentally downregulated. After injury, neuronal intrinsic signals trigger a regenerative program for axonal regrowth and CD133 was found to regulate this regenerative potential. Indeed, CD133 interacted with activin-like kinase (ALK) 4, a type I TGF-β receptor, to synergistically induce phosphorylation of Smad2, which regulates the expression of genes involved in lipid metabolic pathways. Notably, among a set of differentially expressed genes in response to neuronal injury, the downregulation of those associated with cholesterol biosynthesis was specifically observed after CD133 overexpression (Fig. 9) [371]. Thus, this CD133-dependent regulation of cholesterol metabolism associated with TGF-β/Smad signaling may explain the involvement of CD133 as a neuronal intrinsic factor responsible for the regulation of axonal regenerative potential. This exciting example of CD133-related regenerative processes highlights the fact that this lipid raft-associated protein not only directly organizes membrane topology by interacting with membrane cholesterol and gangliosides, but also regulates, in association with certain signaling pathways, sterol and lipid metabolisms.

In a murine model of liver fibrosis, Lee and colleagues reported that CD133 was upregulated in the plasma membrane of fibrotic liver hepatocytes and could interact via its IC1 domain with the N-terminal domain of Smad7 [372], a feedback inhibitor of TGF-β signaling that prevents the TGF-β-induced phosphorylation of Smad2/3 [373, 374]. This CD133–Smad7 interaction inhibited the SMURF2-induced ubiquitination of Smad7 and increased its half-life, resulting in a reduction in the TGF-β-induced liver fibrosis and apoptosis rates of hepatocytes [372]. It is of note that an opposite role for CD133 in fibrogenesis has been proposed on the basis of another murine model of liver injury, where the profibrogenic activity of TGF-β depended on the presence of CD133 [375]. Differences in the identification of CD133-expressing cells, i.e., whether CD133 expression was restricted to progenitor cells or extended to hepatocytes, may account for this discrepancy [372, 375].

During liver regeneration after injury, cytokine IL-6 secreted upon inflammation activation binds to the IL-6 receptor complexed with the signal transducer glycoprotein 130 (gp130) on hepatocytes and triggers various downstream signaling pathways [376]. Recent evidence suggests that CD133 in hepatocytes positively regulates IL-6 signaling by interacting via its EC1 with gp130. This interaction recruits gp130 to lipid rafts, consequently promoting IL-6-induced STAT3 phosphorylation, and thus cell proliferation and liver regeneration [377].

### CD133–radixin signaling regulates cAMP-mediated PKA activation

Spatiotemporal organization of signaling pathway components is a complex process involving intermolecular interactions. Scaffolding proteins play indispensable roles in maintaining the organization of all molecular components. Radixin, a member of the A kinase-anchored protein family, is a scaffolding protein that binds to cyclic adenosine monophosphate (cAMP)-dependent protein kinase A (PKA) to enable phosphorylation of downstream targets [378]. In the mouse liver, radixin mediated the interaction between PKA and proteins involved in glucagon-initiated gluconeogenesis [20, 379]. Interestingly, mouse CD133 directly interacted with radixin through its C-terminal domain and recruited the radixin-PKA complex to the proximity of the glucagon G protein-coupled receptor and adenylyl cyclase at the plasma membrane [20]. This resulted in the activation of adenylyl cyclase and production of cAMP that promoted the separation of the PKA regulatory subunit from the catalytic subunit, which in turn phosphorylated key initiators of gluconeogenesis [20].

### CD133–MAPK/ERK signaling

The crucial role of mitogen-activated protein kinase/extracellular signal-regulated kinase (MAPK/ERK) signaling in cell proliferation, growth, and differentiation is well documented [380]. In a pancreatic carcinoma cell line, CD133 interacts with the ERK1/2/Src complex to enable signal transduction mediated through endothelial growth factor-stimulated ERK1/2 to downstream mediators, including slug (also known as snail family transcriptional repressor 2) [381]. Activation of Slug leads to subsequent N-cadherin expression, which is accompanied by the acquisition of an invasive and pro-metastatic phenotype. Moreover, both ERK1/2 and Src are able to positively regulate the expression of CD133 and thus further promote the CD133-dependent activation of EMT [381, 382]. A similar feedback loop between the MAPK pathway and CD133 expression, which directly affects self-renewal and tumorigenesis, has been described in hepatocellular carcinoma cell lines treated with C-X-C motif chemokine ligand (CXCL)3 [383]. CXCL3 promoted ERK1/2 phosphorylation and the subsequent phosphorylation of the transcription factor ETS proto-oncogene 1, leading to CD133 upregulation. Upregulated CD133 then acted as an upstream activator of CXCL3 and stimulated the growth of hepatocellular carcinoma cells [383].

The importance of CD133-MAPK/ERK signaling in EMT has also been confirmed in vivo. When compared with CD133^−^ counterparts, mouse CD133^+^ melanoma cells were observed to preferentially interact with tumor endothelial cells and establish metastatic foci [143]. The positive regulation of CD133 expression by Notch1 was shown to lead, via CD133-dependent MAPK activation, to the upregulation of activator protein 1 transcription factor, which in turn initiated the expression of matrix metalloproteinases 2 and 9 as well as vascular endothelial growth factor, promoting metastatic potential and tumorigenesis in mouse models [143].

## Conclusion and perspectives

Since its discovery in 1997, the study of CD133 has been an active field covering a wide range of biological topics. In particular, this molecule has been studied in relation to stem cells and CSCs, as well as photoreceptors biogenesis. CD133 has rapidly garnered considerable interest as a prognostic marker and potential target in cancer therapy despite little information about its molecular function in physiological and pathological conditions. Naturally, some conflicting data have been reported as a matter of course. Although there is still no agreement regarding the function of this cholesterol-binding membrane protein, CD133 appears to be involved in a wide array of cellular processes that may be hijacked by cancer cells. These diverse areas nonetheless converge to suggest a fundamental role for CD133 in the dynamics of the cell membrane, including the activity of membrane protrusions, notably the primary cilium, and the release of EVs.

Emerging findings related to various subcellular locations of CD133 (i.e., in membrane protrusions, including microvilli, primary cilium and midbody, or in pericentrosomal and perinuclear regions as well as its inclusion in the nucleus) call for further study of its role(s) in these diverse compartments, and of the mechanisms regulating its intracellular trafficking. As the activation of molecular pathways orchestrating cancer cell self-renewal and metastasis, such as the PI3K-Akt and Src-FAK pathways, depends on the tyrosine phosphorylation status of CD133, particular attention to this specific posttranslational modification may be worthwhile. Although the kinases and tyrosine phosphatases involved have been characterized, the conditions under which these processes are triggered are not fully understood. To fill this gap, we face the challenge of studying the spatiotemporal characteristics of CD133 phosphorylation and associated signal transduction, which may provide important insights into the role of CD133 in different cellular compartments.

CD133^+^ EVs, derived from both normal and cancer cells, have garnered considerable attention in recent years, with ongoing research into the clinical potential of these particles for the diagnosis and monitoring of pathological conditions such as neurodegeneration and other neurological disorders. As reviewed herein, by the means of CD133^+^ EVs delivering various pro-tumorigenic cargoes, cancer cells may communicate to the healthy cells in their vicinity, or at distant sites, the instruction for malignant transformation. From a therapeutic perspective, selective targeting of CD133 with inhibitory anti-CD133 antibodies or small-molecule drugs may, not only eliminate cancer cells [384], but also prevent the release, spreading or uptake of CD133^+^ EVs and thus disrupt tumor tissue growth, and possibly the process of metastasis [272]. A better understanding of the contribution of CD133 to the biogenesis of CD133^+^ EVs is crucial for developing these therapeutic approaches.

As we learned in the past two decades, the mechanisms by which CD133 affects various signaling pathways and cellular processes are diverse and largely relate to the inherent involvement of CD133 at cell membranes as well as to its posttranslational modifications. Therefore, it is important that in future, researchers pay close attention to the subcellular localization of CD133 and its phosphorylation status when reporting correlational or even mechanistic findings describing the role of CD133. Only this detailed information may prevent the misuse of CD133 as a universal marker of cells with stem cell properties, as currently seen in many cancer and stem cell studies. Finally, this knowledge should accelerate ongoing efforts to exploit CD133 in cancer treatment and regenerative medicine.

## Data Availability

Not applicable.

## Abbreviations

Akt: Rac-alpha serine/threonine-protein kinase
ALK: Activin-like kinase
Arl13b: ADP-ribosylation factor-like GTPase 13B
Arp2/3: Actin-related protein 2/3
cAMP: Cyclic adenosine monophosphate
CD: Cluster of differentiation
CXCL: C-X-C motif chemokine ligand
CSC: Cancer stem cell
DFO: Desferrioxamine
DNMT: DNA methyltransferase
ECX: Extracellular domain X (where is 1 to 3)
EGFR: Epidermal growth factor receptor
EMT: Epithelial-mesenchymal transition
ER: Endoplasmic reticulum
ERK: Extracellular signal-regulated kinase
ESCRT: Endosomal sorting complex required for transport
ETS: E-twenty-six or erythroblast transformation specific
EV: Extracellular vesicle
FAK: Focal adhesion kinase
GABARAP: $\upgamma$-aminobutyric type A receptor-associated protein
GLT8D1: Glycosyltransferase 8 domain containing 1
GM_1_: Monosialoganglioside 1
GD_3_: Disialoganglioside 3
HDAC: Histone deacetylase
HIF: Hypoxia inducible factor
HMG: High-mobility group of nuclear proteins
HNSCC: Head and neck squamous cell carcinoma
HSPC: Hematopoietic stem and progenitor cell
ICX: Intracellular domain X (where is 1 to 3)
IL: Interleukin
KRAS: Kirsten rat sarcoma virus oncogene homolog
KLF4: Kruppel-like factor 4
LE/MVB: Late endosome/multivesicular body
LEF: Lymphoid enhancer factor
MAPK: Mitogen-activated protein kinase
MDCK: Madin-Darby canine kidney
MLL: Mixed lineage leukemia
MV: Microvesicle
OCT4: Octamer-binding transcription factor 4
OMIM: Online Mendelian inheritance in man
ORF: Open reading frame
PDK1: 3-Phosphoinositide-dependent protein kinase 1
PDZ: PSD-95/Dig-1/ZO-1
PI3K: Phosphoinositide 3-kinase
PIP_2_: Phosphatidylinositol 4,5-bisphosphate
PIP_3_: Phosphatidylinositol (3,4,5)-trisphosphate
PKA: Protein kinase A
PTPRκ: Protein tyrosine phosphatase κ
RBP-Jκ: Recombination signal binding protein for immunoglobulin kappa J region
ROCK: Rho-associated coiled-coil-containing protein kinase
SOX2: Sex-determining region Y-box 2
SQSTM1: Sequestosome 1
STAT3: Signal transducer and activator of transcription 3
TCF: T-cell factor
TGF: Transforming growth factor
TOR: Target of rapamycin
TNT: Tunneling nanotube
TSG101: Tumor susceptibility gene 101
TTYH: Tweety homolog
ULK1: Unc-51-like autophagy activating kinase 1
UTR: Untranslated region

## References

[1] Weigmann A, Corbeil D, Hellwig A, Huttner WB (1997). Prominin, a novel microvilli-specific polytopic membrane protein of the apical surface of epithelial cells, is targeted to plasmalemmal protrusions of non-epithelial cells. Proc Natl Acad Sci U S A.

[2] Yin AH, Miraglia S, Zanjani ED, Almeida-Porada G, Ogawa M, Leary AG (1997). AC133, a novel marker for human hematopoietic stem and progenitor cells. Blood.

[3] Miraglia S, Godfrey W, Yin AH, Atkins K, Warnke R, Holden JT (1997). A novel five-transmembrane hematopoietic stem cell antigen: isolation, characterization, and molecular cloning. Blood.

[4] Horn PA, Tesch H, Staib P, Schoch C, Kube D, Diehl V, Büchner T, Hiddemann W, Wörmann B, Schellong G, Ritter J, Creutzig U (2001). Significance of AC133 and CD34 expression on acute myeloid leukemia cells. Acute Leukemias VIII: Prognostic Factors and Treatment Strategies.

[5] Bühring HJ, Seiffert M, Marxer A, Weiss B, Faul C, Kanz L, Brugger W (1999). AC133 antigen expression is not restricted to acute myeloid leukemia blasts but is also found on acute lymphoid leukemia blasts and on a subset of CD34+ B-cell precursors. Blood..

[6] Bühring HJ, Seiffert M, Bock TA, Scheding S, Thiel A, Scheffold A (1999). Expression of novel surface antigens on early hematopoietic cells. Ann N Y Acad Sci.

[7] Corbeil D, Röper K, Hellwig A, Tavian M, Miraglia S, Watt SM (2000). The human AC133 hematopoietic stem cell antigen is also expressed in epithelial cells and targeted to plasma membrane protrusions. J Biol Chem.

[8] Maw MA, Corbeil D, Koch J, Hellwig A, Wilson-Wheeler JC, Bridges RJ (2000). A frameshift mutation in prominin (mouse)-like 1 causes human retinal degeneration. Hum Mol Genet.

[9] Corbeil D, Fargeas CA, Huttner WB (2001). Rat prominin, like its mouse and human orthologues, is a pentaspan membrane glycoprotein. Biochem Biophys Res Commun.

[10] Fargeas CA, Joester A, Missol-Kolka E, Hellwig A, Huttner WB, Corbeil D (2004). Identification of novel Prominin-1/CD133 splice variants with alternative C-termini and their expression in epididymis and testis. J Cell Sci.

[11] Florek M, Haase M, Marzesco AM, Freund D, Ehninger G, Huttner WB (2005). Prominin-1/CD133, a neural and hematopoietic stem cell marker, is expressed in adult human differentiated cells and certain types of kidney cancer. Cell Tissue Res.

[12] Jászai J, Farkas LM, Fargeas CA, Janich P, Haase M, Huttner WB (2010). Prominin-2 is a novel marker of distal tubules and collecting ducts of the human and murine kidney. Histochem Cell Biol.

[13] Gashaw I, Dushaj O, Behr R, Biermann K, Brehm R, Rubben H (2007). Novel germ cell markers characterize testicular seminoma and fetal testis. Mol Hum Reprod.

[14] Pereira MF, Fernandes SA, Nascimento AR, Siu ER, Hess RA, Oliveira CA (2014). Effects of the oestrogen receptor antagonist Fulvestrant on expression of genes that affect organization of the epididymal epithelium. Andrology.

[15] Jászai J, Janich P, Farkas LM, Fargeas CA, Huttner WB, Corbeil D (2007). Differential expression of Prominin-1 (CD133) and Prominin-2 in major cephalic exocrine glands of adult mice. Histochem Cell Biol.

[16] Immervoll H, Hoem D, Sakariassen PØ, Steffensen OJ, Molven A (2008). Expression of the “stem cell marker” CD133 in pancreas and pancreatic ductal adenocarcinomas. BMC Cancer.

[17] Karbanová J, Missol-Kolka E, Fonseca A-V, Lorra C, Janich P, Hollerová H (2008). The stem cell marker CD133 (Prominin-1) is expressed in various human glandular epithelia. J Histochem Cytochem.

[18] Anderson LH, Boulanger CA, Smith GH, Carmeliet P, Watson CJ (2011). Stem cell marker prominin-1 regulates branching morphogenesis, but not regenerative capacity, in the mammary gland. Dev Dyn.

[19] Suzuki A, Sekiya S, Onishi M, Oshima N, Kiyonari H, Nakauchi H (2008). Flow cytometric isolation and clonal identification of self-renewing bipotent hepatic progenitor cells in adult mouse liver. Hepatology.

[20] Lee H, Yu DM, Park JS, Lee H, Kim JS, Kim HL (2020). Prominin-1-Radixin axis controls hepatic gluconeogenesis by regulating PKA activity. EMBO Rep.

[21] Lardon J, Corbeil D, Huttner WB, Ling Z, Bouwens L (2008). Stem cell marker prominin-1/AC133 is expressed in duct cells of the adult human pancreas. Pancreas.

[22] Corbeil D, Röper K, Fargeas CA, Joester A, Huttner WB (2001). Prominin: a story of cholesterol, plasma membrane protrusions and human pathology. Traffic.

[23] Zacchigna S, Oh H, Wilsch-Bräuninger M, Missol-Kolka E, Jászai J, Jansen S (2009). Loss of the cholesterol-binding protein prominin-1/CD133 causes disk dysmorphogenesis and photoreceptor degeneration. J Neurosci.

[24] Karbanová J, Laco J, Marzesco A-M, Janich P, Voborníková M, Mokrý J (2014). Human prominin-1 (CD133) is detected in both neoplastic and non-neoplastic salivary gland diseases and released into saliva in a ubiquitinated form. PLoS ONE.

[25] Jászai J, Fargeas CA, Graupner S, Tanaka EM, Brand M, Huttner WB (2011). Distinct and conserved prominin-1/CD133–positive retinal cell populations identified across species. PLoS ONE.

[26] Corbeil D, Joester A, Fargeas CA, Jászai J, Garwood J, Hellwig A (2009). Expression of distinct splice variants of the stem cell marker prominin-1 (CD133) in glial cells. Glia.

[27] Fargeas CA, Fonseca A-V, Huttner WB, Corbeil D (2006). Prominin-1 (CD133): from progenitor cells to human diseases. Future Lipidol.

[28] Singh SK, Clarke ID, Terasaki M, Bonn VE, Hawkins C, Squire J (2003). Identification of a cancer stem cell in human brain tumors. Cancer Res.

[29] Singh SK, Hawkins C, Clarke ID, Squire JA, Bayani J, Hide T (2004). Identification of human brain tumour initiating cells. Nature.

[30] Hemmati HD, Nakano I, Lazareff JA, Masterman-Smith M, Geschwind DH, Bronner-Fraser M (2003). Cancerous stem cells can arise from pediatric brain tumors. Proc Natl Acad Sci U S A.

[31] Zeppernick F, Ahmadi R, Campos B, Dictus C, Helmke BM, Becker N (2008). Stem cell marker CD133 affects clinical outcome in glioma patients. Clin Cancer Res.

[32] Zhang M, Song T, Yang L, Chen R, Wu L, Yang Z (2008). Nestin and CD133: valuable stem cell-specific markers for determining clinical outcome of glioma patients. J Exp Clin Cancer Res.

[33] Liou GY (2019). CD133 as a regulator of cancer metastasis through the cancer stem cells. Int J Biochem Cell Biol.

[34] Grosse-Gehling P, Fargeas CA, Dittfeld C, Garbe Y, Alison MR, Corbeil D (2013). CD133 as a biomarker for putative cancer stem cells in solid tumours: limitations, problems and challenges. J Pathol.

[35] Uchida N, Buck DW, He D, Reitsma MJ, Masek M, Phan TV (2000). Direct isolation of human central nervous system stem cells. Proc Natl Acad Sci U S A.

[36] Lee A, Kessler JD, Read TA, Kaiser C, Corbeil D, Huttner WB (2005). Isolation of neural stem cells from the postnatal cerebellum. Nat Neurosci.

[37] Beier D, Hau P, Proescholdt M, Lohmeier A, Wischhusen J, Oefner PJ (2007). CD133(+) and CD133(-) glioblastoma-derived cancer stem cells show differential growth characteristics and molecular profiles. Cancer Res.

[38] Richardson GD, Robson CN, Lang SH, Neal DE, Maitland NJ, Collins AT (2004). CD133, a novel marker for human prostatic epithelial stem cells. J Cell Sci.

[39] Miki J, Furusato B, Li H, Gu Y, Takahashi H, Egawa S (2007). Identification of putative stem cell markers, CD133 and CXCR4, in hTERT–immortalized primary nonmalignant and malignant tumor-derived human prostate epithelial cell lines and in prostate cancer specimens. Cancer Res.

[40] Vander Griend DJ, Karthaus WL, Dalrymple S, Meeker A, Demarzo AM, Isaacs JT (2008). The role of CD133 in normal human prostate stem cells and malignant cancer-initiating cells. Cancer Res.

[41] Missol-Kolka E, Karbanová J, Janich P, Haase M, Fargeas CA, Huttner WB (2011). Prominin-1 (CD133) is not restricted to stem cells located in the basal compartment of murine and human prostate. Prostate.

[42] Collins AT, Berry PA, Hyde C, Stower MJ, Maitland NJ (2005). Prospective identification of tumorigenic prostate cancer stem cells. Cancer Res.

[43] Bussolati B, Bruno S, Grange C, Buttiglieri S, Deregibus MC, Cantino D (2005). Isolation of renal progenitor cells from adult human kidney. Am J Pathol.

[44] Angelotti ML, Ronconi E, Ballerini L, Peired A, Mazzinghi B, Sagrinati C (2012). Characterization of renal progenitors committed toward tubular lineage and their regenerative potential in renal tubular injury. Stem Cells.

[45] Sagrinati C, Netti GS, Mazzinghi B, Lazzeri E, Liotta F, Frosali F (2006). Isolation and characterization of multipotent progenitor cells from the Bowman's capsule of adult human kidneys. J Am Soc Nephrol.

[46] Lindgren D, Boström AK, Nilsson K, Hansson J, Sjölund J, Möller C (2011). Isolation and characterization of progenitor-like cells from human renal proximal tubules. Am J Pathol.

[47] Suetsugu A, Nagaki M, Aoki H, Motohashi T, Kunisada T, Moriwaki H (2006). Characterization of CD133+ hepatocellular carcinoma cells as cancer stem/progenitor cells. Biochem Biophys Res Commun.

[48] Kordes C, Sawitza I, Müller-Marbach A, Ale-Agha N, Keitel V, Klonowski-Stumpe H (2007). CD133+ hepatic stellate cells are progenitor cells. Biochem Biophys Res Commun.

[49] Dorrell C, Erker L, Schug J, Kopp JL, Canaday PS, Fox AJ (2011). Prospective isolation of a bipotential clonogenic liver progenitor cell in adult mice. Genes Dev.

[50] Oshima Y, Suzuki A, Kawashimo K, Ishikawa M, Ohkohchi N, Taniguchi H (2007). Isolation of mouse pancreatic ductal progenitor cells expressing CD133 and c-Met by flow cytometric cell sorting. Gastroenterology.

[51] Hermann PC, Huber SL, Herrler T, Aicher A, Ellwart JW, Guba M (2007). Distinct populations of cancer stem cells determine tumor growth and metastatic activity in human pancreatic cancer. Cell Stem Cell.

[52] O'Brien CA, Pollett A, Gallinger S, Dick JE (2007). A human colon cancer cell capable of initiating tumour growth in immunodeficient mice. Nature.

[53] Ricci-Vitiani L, Lombardi DG, Pilozzi E, Biffoni M, Todaro M, Peschle C (2007). Identification and expansion of human colon-cancer-initiating cells. Nature.

[54] Zhu L, Gibson P, Currle DS, Tong Y, Richardson RJ, Bayazitov IT (2009). Prominin 1 marks intestinal stem cells that are susceptible to neoplastic transformation. Nature.

[55] Snippert HJ, van Es JH, van den Born M, Begthel H, Stange DE, Barker N (2009). Prominin-1/CD133 marks stem cells and early progenitors in mouse small intestine. Gastroenterology.

[56] Eramo A, Lotti F, Sette G, Pilozzi E, Biffoni M, Di Virgilio A (2008). Identification and expansion of the tumorigenic lung cancer stem cell population. Cell Death Differ.

[57] Monzani E, Facchetti F, Galmozzi E, Corsini E, Benetti A, Cavazzin C (2007). Melanoma contains CD133 and ABCG2 positive cells with enhanced tumourigenic potential. Eur J Cancer.

[58] Ito Y, Hamazaki TS, Ohnuma K, Tamaki K, Asashima M, Okochi H (2007). Isolation of murine hair-inducing cells using the cell surface marker prominin-1/CD133. J Invest Dermatol.

[59] Kratz-Albers K, Zühlsdorp M, Leo R, Berdel WL, Büchner T, Serve H (1998). Expression of a AC133, a novel stem cell marker, on human leukemic blasts lacking CD34-antigen and on a human CD34+ leukemic line:MUTZ-2. Blood.

[60] Horn PA, Tesch H, Staib P, Kube D, Diehl V, Voliotis D (1999). Expression of AC133, a novel hematopoietic precursor antigen, on acute myeloid leukemia cells. Blood.

[61] Wuchter C, Ratei R, Spahn G, Schoch, C, Harbott J, Schnittger, S et al. Impact of CD133 (AC133) and CD90 expression analysis for acute leukemia immunophenotyping. Haematologica. 2001;86:154-61. 11224484

[62] Cox CV, Diamanti P, Evely RS, Kearns PR, Blair A (2009). Expression of CD133 on leukemia-initiating cells in childhood ALL. Blood.

[63] Peichev M, Naiyer AJ, Pereira D, Zhu Z, Lane WJ, Williams M (2000). Expression of VEGFR-2 and AC133 by circulating human CD34(+) cells identifies a population of functional endothelial precursors. Blood.

[64] Gehling UM, Ergün S, Schumacher U, Wagener C, Pantel K, Otte M (2000). In vitro differentiation of endothelial cells from AC133-positive progenitor cells. Blood.

[65] Hilbe W, Dirnhofer S, Oberwasserlechner F, Schmid T, Gunsilius E, Hilbe G (2004). CD133 positive endothelial progenitor cells contribute to the tumour vasculature in non-small cell lung cancer. J Clin Pathol.

[66] Quirici N, Soligo D, Caneva L, Servida F, Bossolasco P, Deliliers GL (2001). Differentiation and expansion of endothelial cells from human bone marrow CD133(+) cells. Br J Haematol.

[67] Sekine A, Nishiwaki T, Nishimura R, Kawasaki T, Urushibara T, Suda R (2016). Prominin-1/CD133 expression as potential tissue-resident vascular endothelial progenitor cells in the pulmonary circulation. Am J Physiol Lung Cell Mol Physiol.

[68] Rossi E, Poirault-Chassac S, Bieche I, Chocron R, Schnitzler A, Lokajczyk A (2019). Human endothelial colony forming cells express intracellular CD133 that modulates their vasculogenic properties. Stem Cell Rev Rep.

[69] Sun S, Meng Y, Li M, Tang X, Hu W, Wu W (2023). CD133(+) endothelial-like stem cells restore neovascularization and promote longevity in progeroid and naturally aged mice. Nat Aging.

[70] Corbeil D, Fargeas CA, Jászai J (2014). CD133 might be a pan marker of epithelial cells with dedifferentiation capacity. Proc Natl Acad Sci U S A.

[71] Jászai J, Graupner S, Tanaka EM, Funk RHW, Huttner WB, Brand M (2013). Spatial distribution of prominin-1 (CD133)—positive cells within germinative zones of the vertebrate brain. PLoS ONE.

[72] Kusaba T, Lalli M, Kramann R, Kobayashi A, Humphreys BD (2014). Differentiated kidney epithelial cells repair injured proximal tubule. Proc Natl Acad Sci U S A.

[73] Kramann R, Kusaba T, Humphreys BD (2015). Who regenerates the kidney tubule?. Nephrol Dial Transplant.

[74] Cheng JX, Liu BL, Zhang X (2009). How powerful is CD133 as a cancer stem cell marker in brain tumors?. Cancer Treat Rev.

[75] Miraglia S, Godfrey W, Buck D (1998). A response to AC133 hematopoietic stem cell antigen: human homologue of mouse kidney prominin or distinct member of a novel protein family?. Blood.

[76] Shmelkov SV, Butler JM, Hooper AT, Hormigo A, Kushner J, Milde T (2008). CD133 expression is not restricted to stem cells, and both CD133(+) and CD133(-) metastatic colon cancer cells initiate tumors. J Clin Invest.

[77] Fargeas CA, Corbeil D, Huttner WB (2003). AC133 antigen, CD133, prominin-1, prominin-2, etc.: prominin family gene products in need of a rational nomenclature. Stem Cells.

[78] Bidlingmaier S, Zhu X, Liu B (2008). The utility and limitations of glycosylated human CD133 epitopes in defining cancer stem cells. J Mol Med.

[79] Jaksch M, Múnera J, Bajpai R, Terskikh A, Oshima RG (2008). Cell cycle-dependent variation of a CD133 epitope in human embryonic stem cell, colon cancer, and melanoma cell lines. Cancer Res.

[80] Kemper K, Sprick MR, de Bree M, Scopelliti A, Vermeulen L, Hoek M (2010). The AC133 epitope, but not the CD133 protein, is lost upon cancer stem cell differentiation. Cancer Res.

[81] Bauer N, Wilsch-Bräuninger M, Karbanová J, Fonseca AV, Strauss D, Freund D (2011). Haematopoietic stem cell differentiation promotes the release of prominin-1/CD133-containing membrane vesicles—a role of the endocytic–exocytic pathway. EMBO Mol Med.

[82] Fargeas CA, Karbanová J, Jászai J, Corbeil D (2011). CD133 and membrane microdomains: old facets for future hypotheses. World J Gastroenterol.

[83] Barrantes-Freer A, Renovanz M, Eich M, Braukmann A, Sprang B, Spirin P (2015). CD133 expression is not synonymous to immunoreactivity for AC133 and fluctuates throughout the cell cycle in glioma stem-like cells. PLoS ONE.

[84] Fargeas CA, Büttner E, Corbeil D (2015). Commentary: “Prom1 function in development, intestinal inflammation, and intestinal tumorigenesis”. Front Oncol.

[85] Cehajic-Kapetanovic J, Birtel J, McClements ME, Shanks ME, Clouston P, Downes SM (2019). Clinical and molecular characterization of PROM1-related retinal degeneration. JAMA Netw Open.

[86] Zhang Q, Zulfiqar F, Xiao X, Riazuddin S, Ahmad Z, Caruso R (2007). Severe retinitis pigmentosa mapped to 4p15 and associated with a novel mutation in the PROM1 gene. Hum Genet.

[87] Yang Z, Chen Y, Lillo C, Chien J, Yu Z, Michaelides M (2008). Mutant prominin 1 found in patients with macular degeneration disrupts photoreceptor disk morphogenesis in mice. J Clin Invest.

[88] Pras E, Abu A, Rotenstreich Y, Avni I, Reish O, Morad Y (2009). Cone-rod dystrophy and a frameshift mutation in the PROM1 gene. Mol Vis.

[89] Sleeman KE, Kendrick H, Robertson D, Isacke CM, Ashworth A, Smalley MJ (2007). Dissociation of estrogen receptor expression and in vivo stem cell activity in the mammary gland. J Cell Biol.

[90] Ford MJ, Harwalkar K, Kazemdarvish H, Yamanaka N, Yamanaka Y (2023). CD133/Prom1 marks proximal mouse oviduct epithelial progenitors and adult epithelial cells with a low generative capacity. Biol Open.

[91] Arndt K, Grinenko T, Mende N, Reichert D, Portz M, Ripich T (2013). CD133 is a modifier of hematopoietic progenitor frequencies but is dispensable for the maintenance of mouse hematopoietic stem cells. Proc Natl Acad Sci U S A.

[92] Walker TL, Wierick A, Sykes AM, Waldau B, Corbeil D, Carmeliet P (2013). Prominin-1 allows prospective isolation of neural stem cells from the adult murine hippocampus. J Neurosci.

[93] Fargeas CA, Florek M, Huttner WB, Corbeil D (2003). Characterization of Prominin-2, a new member of the prominin family of pentaspan membrane glycoproteins. J Biol Chem.

[94] Liu Y, Ren S, Xie L, Cui C, Xing Y, Liu C (2015). Mutation of N-linked glycosylation at Asn548 in CD133 decreases its ability to promote hepatoma cell growth. Oncotarget.

[95] Corbeil D, Karbanová J, Fargeas CA, Jászai J (2013). Prominin-1 (CD133): molecular and cellular features across species. Adv Exp Med Biol.

[96] Han Z, Papermaster DS (2011). Identification of three prominin homologs and characterization of their messenger RNA expression in Xenopus laevis tissues. Mol Vis.

[97] Fargeas CA (2013). Prominin-2 and other relatives of CD133. Adv Exp Med Biol.

[98] Zelhof AC, Hardy RW, Becker A, Zuker CS (2006). Transforming the architecture of compound eyes. Nature.

[99] Demontis F, Dahmann C (2007). Apical and lateral cell protrusions interconnect epithelial cells in live Drosophila wing imaginal discs. Dev Dyn.

[100] Nie J, Mahato S, Mustill W, Tipping C, Bhattacharya SS, Zelhof AC (2012). Cross species analysis of Prominin reveals a conserved cellular role in invertebrate and vertebrate photoreceptor cells. Dev Biol.

[101] Mahato S, Nie J, Plachetzki DC, Zelhof AC (2018). A mosaic of independent innovations involving eyes shut are critical for the evolutionary transition from fused to open rhabdoms. Dev Biol.

[102] Wang X, Zheng H, Jia Z, Lei Z, Li M, Zhuang Q (2019). Drosophila Prominin-like, a homolog of CD133, interacts with ND20 to maintain mitochondrial function. Cell Biosci.

[103] McGrail M, Batz L, Noack K, Pandey S, Huang Y, Gu X (2010). Expression of the zebrafish CD133/prominin1 genes in cellular proliferation zones in the embryonic central nervous system and sensory organs. Dev Dyn.

[104] Fargeas CA, Huttner WB, Corbeil D (2007). Nomenclature of prominin-1 (CD133) splice variants—an update. Tissue Antigens.

[105] Thamm K, Graupner S, Werner C, Huttner WB, Corbeil D (2016). Monoclonal antibodies 13A4 and AC133 do not recognize the canine ortholog of mouse and human stem cell antigen prominin-1 (CD133). PLoS ONE.

[106] Shmelkov SV, Jun L, St Clair R, McGarrigle D, Derderian CA, Usenko JK (2004). Alternative promoters regulate transcription of the gene that encodes stem cell surface protein AC133. Blood.

[107] Kemper K, Tol MJPM, Medema JP (2010). Mouse tissues express multiple splice variants of prominin-1. PLoS ONE.

[108] Choi M-H, Na JE, Yoon YR, Rhyu IJ, Ko Y-G, Baik J-H (2018). Hypomyelination and cognitive impairment in mice lacking CD133 (Prominin-1). Biochem Biophys Res Commun.

[109] Osmond TL, Broadley KWR, McConnell MJ (2010). Glioblastoma cells negative for the anti-CD133 antibody AC133 express a truncated variant of the CD133 protein. Int J Mol Med.

[110] Gopisetty G, Xu J, Sampath D, Colman H, Puduvalli VK (2013). Epigenetic regulation of CD133/PROM1 expression in glioma stem cells by Sp1/myc and promoter methylation. Oncogene.

[111] Sompallae R, Hofmann O, Maher C, Gedye C, Behren A, Vitezic M (2013). A comprehensive promoter landscape identifies a novel promoter for CD133 in restricted tissues, cancers, and stem cells. Front Genet.

[112] Tabu K, Bizen N, Taga T, Tanaka S (2013). Gene regulation of prominin-1 (CD133) in normal and cancerous tissues. Adv Exp Med Biol.

[113] Tabu K, Kimura T, Sasai K, Wang L, Bizen N, Nishihara H (2010). Analysis of an alternative human CD133 promoter reveals the implication of Ras/ERK pathway in tumor stem-like hallmarks. Mol Cancer.

[114] Tabu K, Sasai K, Kimura T, Wang L, Aoyanagi E, Kohsaka S (2008). Promoter hypomethylation regulates CD133 expression in human gliomas. Cell Res.

[115] Pleshkan VV, Vinogradova TV, Sverdlov ED (2008). Methylation of the prominin 1 TATA-less main promoters and tissue specificity of their transcript content. Biochim Biophys Acta.

[116] Yi JM, Tsai HC, Glöckner SC, Lin S, Ohm JE, Easwaran H (2008). Abnormal DNA methylation of CD133 in colorectal and glioblastoma tumors. Cancer Res.

[117] Baba T, Convery PA, Matsumura N, Whitaker RS, Kondoh E, Perry T (2009). Epigenetic regulation of CD133 and tumorigenicity of CD133+ ovarian cancer cells. Oncogene.

[118] Godfrey L, Crump NT, O'Byrne S, Lau IJ, Rice S, Harman JR (2021). H3K79me2/3 controls enhancer-promoter interactions and activation of the pan-cancer stem cell marker PROM1/CD133 in MLL-AF4 leukemia cells. Leukemia.

[119] Guenther MG, Lawton LN, Rozovskaia T, Frampton GM, Levine SS, Volkert TL (2008). Aberrant chromatin at genes encoding stem cell regulators in human mixed-lineage leukemia. Genes Dev.

[120] Mak AB, Nixon AML, Moffat J (2012). The mixed lineage leukemia (MLL) fusion-associated gene AF4 promotes CD133 transcription. Cancer Res.

[121] You H, Ding W, Rountree CB (2010). Epigenetic regulation of cancer stem cell marker CD133 by transforming growth factor-β. Hepatology.

[122] Chen Z, Chen Y, Li Y, Lian W, Zheng K, Zhang Y (2021). Prrx1 promotes stemness and angiogenesis via activating TGF-β/smad pathway and upregulating proangiogenic factors in glioma. Cell Death Dis.

[123] Cave DD, Di Guida M, Costa V, Sevillano M, Ferrante L, Heeschen C (2020). TGF-β1 secreted by pancreatic stellate cells promotes stemness and tumourigenicity in pancreatic cancer cells through L1CAM downregulation. Oncogene.

[124] Pellacani D, Packer RJ, Frame FM, Oldridge EE, Berry PA, Labarthe M-C (2011). Regulation of the stem cell marker CD133 is independent of promoter hypermethylation in human epithelial differentiation and cancer. Mol Cancer.

[125] D'Anello L, Sansone P, Storci G, Mitrugno V, D'Uva G, Chieco P (2010). Epigenetic control of the basal-like gene expression profile via Interleukin-6 in breast cancer cells. Mol Cancer.

[126] Hafner A, Bulyk ML, Jambhekar A, Lahav G (2019). The multiple mechanisms that regulate p53 activity and cell fate. Nat Rev Mol Cell Biol.

[127] Park EK, Lee JC, Park JW, Bang SY, Yi SA, Kim BK (2015). Transcriptional repression of cancer stem cell marker CD133 by tumor suppressor p53. Cell Death Dis.

[128] Blazek ER, Foutch JL, Maki G (2007). Daoy medulloblastoma cells that express CD133 are radioresistant relative to CD133− cells, and the CD133+ sector is enlarged by hypoxia. Int J Radiat Oncol Biol Phys.

[129] Platet N, Liu SY, Atifi ME, Oliver L, Vallette FM, Berger F (2007). Influence of oxygen tension on CD133 phenotype in human glioma cell cultures. Cancer Lett.

[130] Bar EE, Lin A, Mahairaki V, Matsui W, Eberhart CG (2010). Hypoxia increases the expression of stem-cell markers and promotes clonogenicity in glioblastoma neurospheres. Am J Pathol.

[131] Filatova A, Seidel S, Böğürcü N, Gräf S, Garvalov BK, Acker T (2016). Acidosis acts through HSP90 in a PHD/VHL-independent manner to promote HIF function and stem cell maintenance in glioma. Cancer Res.

[132] Soeda A, Park M, Lee D, Mintz A, Androutsellis-Theotokis A, McKay RD (2009). Hypoxia promotes expansion of the CD133-positive glioma stem cells through activation of HIF-1alpha. Oncogene.

[133] Maeda K, Ding Q, Yoshimitsu M, Kuwahata T, Miyazaki Y, Tsukasa K (2016). CD133 modulate HIF-1α expression under hypoxia in EMT phenotype pancreatic cancer stem-like cells. Int J Mol Sci.

[134] Qin J, Liu Y, Lu Y, Liu M, Li M, Li J (2017). Hypoxia-inducible factor 1 alpha promotes cancer stem cells-like properties in human ovarian cancer cells by upregulating SIRT1 expression. Sci Rep.

[135] Choudhry H, Harris AL (2018). Advances in hypoxia-inducible factor biology. Cell Metab.

[136] Ohnishi S, Maehara O, Nakagawa K, Kameya A, Otaki K, Fujita H (2013). Hypoxia-inducible factors activate CD133 promoter through ETS family transcription factors. PLoS ONE.

[137] Iida H, Suzuki M, Goitsuka R, Ueno H (2012). Hypoxia induces CD133 expression in human lung cancer cells by up-regulation of OCT3/4 and SOX2. Int J Oncol.

[138] Won C, Kim BH, Yi EH, Choi KJ, Kim EK, Jeong JM (2015). Signal transducer and activator of transcription 3-mediated CD133 up-regulation contributes to promotion of hepatocellular carcinoma. Hepatology.

[139] Matsumoto K, Arao T, Tanaka K, Kaneda H, Kudo K, Fujita Y (2009). mTOR signal and hypoxia-inducible factor-1 regulate CD133 expression in cancer cells. Cancer Res.

[140] Liu YP, Yang CJ, Huang MS, Yeh CT, Wu AT, Lee YC (2013). Cisplatin selects for multidrug-resistant CD133+ cells in lung adenocarcinoma by activating Notch signaling. Cancer Res.

[141] Fan X, Khaki L, Zhu TS, Soules ME, Talsma CE, Gul N (2010). NOTCH pathway blockade depletes CD133-positive glioblastoma cells and inhibits growth of tumor neurospheres and xenografts. Stem Cells.

[142] Konishi H, Asano N, Imatani A, Kimura O, Kondo Y, Jin X (2016). Notch1 directly induced CD133 expression in human diffuse type gastric cancers. Oncotarget.

[143] Kumar D, Kumar S, Gorain M, Tomar D, Patil HS, Radharani NNV (2016). Notch1-MAPK signaling axis regulates CD133+ cancer stem cell-mediated melanoma growth and angiogenesis. J Invest Dermatol.

[144] Katoh Y, Katoh M (2007). Comparative genomics on PROM1 gene encoding stem cell marker CD133. Int J Mol Med.

[145] Emami KH, Nguyen C, Ma H, Kim DH, Jeong KW, Eguchi M (2004). A small molecule inhibitor of beta-catenin/CREB-binding protein transcription. Proc Natl Acad Sci U S A.

[146] Tang Y, Berlind J, Mavila N (2018). Inhibition of CREB binding protein-beta-catenin signaling down regulates CD133 expression and activates PP2A-PTEN signaling in tumor initiating liver cancer cells. Cell Commun Signal.

[147] Inui M, Martello G, Piccolo S (2010). MicroRNA control of signal transduction. Nat Rev Mol Cell Biol.

[148] Yoshida K, Yamamoto Y, Ochiya T (2021). miRNA signaling networks in cancer stem cells. Regen Ther.

[149] Bai H-Y, Liao Y-J, Cai M-Y, Ma N-F, Zhang Q, Chen J-W (2018). Eukaryotic initiation factor 5A2 contributes to the maintenance of CD133(+) hepatocellular carcinoma cells via the c-Myc/microRNA-29b Axis. Stem Cells.

[150] Li B, Xu WW, Han L, Chan KT, Tsao SW, Lee NPY (2017). MicroRNA-377 suppresses initiation and progression of esophageal cancer by inhibiting CD133 and VEGF. Oncogene.

[151] Huang S-X, Zhao Z-Y, Weng G-H, He X-Y, Wu C-J, Fu C-Y (2017). Upregulation of miR-181a suppresses the formation of glioblastoma stem cells by targeting the Notch2 oncogene and correlates with good prognosis in patients with glioblastoma multiforme. Biochem Biophys Res Commun.

[152] Bourseau-Guilmain E, Griveau A, Benoit J-P, Garcion E (2011). The importance of the stem cell marker prominin-1/CD133 in the uptake of transferrin and in iron metabolism in human colon cancer Caco-2 cells. PLoS ONE.

[153] Zhou F, Cui C, Ge Y, Chen H, Li Q, Yang Z (2010). α2,3-Sialylation regulates the stability of stem cell marker CD133. J Biochem.

[154] Hamanoue M, Matsuzaki Y, Sato K-I, Okano HJ, Shibata S, Sato I (2009). Cell surface N-glycans mediated isolation of mouse neural stem cells. J Neurochem.

[155] Dowland SN, Madawala RJ, Poon CE, Lindsay LA, Murphy CR (2017). Prominin-1 glycosylation changes throughout early pregnancy in uterine epithelial cells under the influence of maternal ovarian hormones. Reprod Fertil Dev.

[156] Lehnus KS, Donovan LK, Huang X, Zhao N, Warr TJ, Pilkington GJ (2013). CD133 glycosylation is enhanced by hypoxia in cultured glioma stem cells. Int J Oncol.

[157] Liu K, Jiang L, Shi Y, Liu B, He Y, Shen Q (2022). Hypoxia-induced GLT8D1 promotes glioma stem cell maintenance by inhibiting CD133 degradation through N-linked glycosylation. Cell Death Differ.

[158] Wei Y, Chen Q, Huang S, Liu Y, Li Y, Xing Y (2022). The interaction between DNMT1 and high-mannose CD133 maintains the slow-cycling state and tumorigenic potential of glioma stem cell. Adv Sci.

[159] Mak AB, Blakely KM, Williams RA, Penttilä P-A, Shukalyuk AI, Osman KT (2011). CD133 protein N-glycosylation processing contributes to cell surface recognition of the primitive cell marker AC133 epitope. J Biol Chem.

[160] Joo KM, Kim SY, Jin X, Song SY, Kong DS, Lee JI (2008). Clinical and biological implications of CD133-positive and CD133-negative cells in glioblastomas. Lab Invest.

[161] Boivin D, Labbé D, Fontaine N, Lamy S, Beaulieu ED, Gingras D (2009). The stem cell marker CD133 (Prominin-1) is phosphorylated on cytoplasmic tyrosine-828 and tyrosine-852 by Src and Fyn tyrosine kinases. Biochemistry.

[162] Mak AB, Pehar M, Nixon AML, Williams RA, Uetrecht AC, Puglielli L (2014). Post-translational regulation of CD133 by ATase1/ATase2-mediated lysine acetylation. J Mol Biol.

[163] Wei Y, Jiang Y, Zou F, Liu Y, Wang S, Xu N (2013). Activation of PI3K/Akt pathway by CD133-p85 interaction promotes tumorigenic capacity of glioma stem cells. Proc Natl Acad Sci U S A.

[164] Chen Y-S, Wu M-J, Huang C-Y, Lin S-C, Chuang T-H, Yu C-C (2011). CD133/Src axis mediates tumor initiating property and epithelial-mesenchymal transition of head and neck cancer. PLoS ONE.

[165] Yang F, Xing Y, Li Y, Chen X, Jiang J, Ai Z (2018). Monoubiquitination of cancer stem cell marker CD133 at lysine 848 regulates its secretion and promotes cell migration. Mol Cell Biol.

[166] Babst M, Odorizzi G, Estepa EJ, Emr SD (2000). Mammalian tumor susceptibility gene 101 (TSG101) and the yeast homologue, Vps23p, both function in late endosomal trafficking. Traffic.

[167] Baietti MF, Zhang Z, Mortier E, Melchior A, Degeest G, Geeraerts A (2012). Syndecan-syntenin-ALIX regulates the biogenesis of exosomes. Nat Cell Biol.

[168] Ding Y, Dellisanti CD, Ko MH, Czajkowski C, Puglielli L (2014). The endoplasmic reticulum-based acetyltransferases, ATase1 and ATase2, associate with the oligosaccharyltransferase to acetylate correctly folded polypeptides. J Biol Chem.

[169] Jászai J, Fargeas CA, Florek M, Huttner WB, Corbeil D (2007). Focus on molecules: prominin-1 (CD133). Exp Eye Res.

[170] Gurudev N, Florek M, Corbeil D, Knust E (2013). Prominent role of prominin in the retina. Adv Exp Med Biol.

[171] Goldberg AFX, Moritz OL, Williams DS (2016). Molecular basis for photoreceptor outer segment architecture. Prog Retin Eye Res.

[172] Dellett M, Sasai N, Nishide K, Becker S, Papadaki V, Limb GA (2015). Genetic background and light-dependent progression of photoreceptor cell degeneration in prominin-1 knockout Mice. Invest Ophthalmol Vis Sci.

[173] Han Z, Anderson DW, Papermaster DS (2012). Prominin-1 localizes to the open rims of outer segment lamellae in xenopus laevis rod and cone photoreceptors. Invest Ophthalmol Vis Sci.

[174] Carr BJ, Stanar P, Moritz OL (2021). Distinct roles for prominin-1 and photoreceptor cadherin in outer segment disc morphogenesis in CRISPR-altered *X. laevis*. J Cell Sci.

[175] Lu Z, Hu X, Reilly J, Jia D, Liu F, Yu S (2019). Deletion of the transmembrane protein Prom1b in zebrafish disrupts outer-segment morphogenesis and causes photoreceptor degeneration. J Biol Chem.

[176] Corbeil D, Fargeas CA, Jászai J (2019). Deciphering the roles of prominins in the visual system. J Biol Chem.

[177] Thamm K, Šimaitė D, Karbanová J, Bermúdez V, Reichert D, Morgenstern A (2019). Prominin-1 (CD133) modulates the architecture and dynamics of microvilli. Traffic.

[178] Hori A, Nishide K, Yasukuni Y, Haga K, Kakuta W, Ishikawa Y (2019). Prominin-1 modulates Rho/ROCK-mediated membrane morphology and calcium-dependent intracellular chloride flux. Sci Rep.

[179] Freund D, Bauer N, Boxberger S, Feldmann S, Streller U, Ehninger G (2006). Polarization of human hematopoietic progenitors during contact with multipotent mesenchymal stromal cells: effects on proliferation and clonogenicity. Stem Cells Dev.

[180] Spencer WJ, Schneider NF, Lewis TR, Castillo CM, Skiba NP, Arshavsky VY (2023). The WAVE complex drives the morphogenesis of the photoreceptor outer segment cilium. Proc Natl Acad Sci U S A.

[181] Goetz SC, Anderson KV (2010). The primary cilium: a signalling centre during vertebrate development. Nat Rev Genet.

[182] Singer D, Thamm K, Zhuang H, Karbanová J, Gao Y, Walker JV (2019). Prominin-1 controls stem cell activation by orchestrating ciliary dynamics. EMBO J.

[183] Jászai J, Thamm K, Karbanová J, Janich P, Fargeas CA, Huttner WB (2020). Prominins control ciliary length throughout the animal kingdom: new lessons from human prominin-1 and zebrafish prominin-3. J Biol Chem.

[184] Florek M, Bauer N, Janich P, Wilsch-Braeuninger M, Fargeas CA, Marzesco AM (2007). Prominin-2 is a cholesterol-binding protein associated with apical and basolateral plasmalemmal protrusions in polarized epithelial cells and released into urine. Cell Tissue Res.

[185] Janich P, Corbeil D (2007). GM1 and GM3 gangliosides highlight distinct lipid microdomains within the apical domain of epithelial cells. FEBS Lett.

[186] Larkins CE, Aviles GDG, East MP, Kahn RA, Caspary T (2011). Arl13b regulates ciliogenesis and the dynamic localization of Shh signaling proteins. Mol Biol Cell.

[187] Lu H, Toh MT, Narasimhan V, Thamilselvam SK, Choksi SP, Roy S (2015). A function for the Joubert syndrome protein Arl13b in ciliary membrane extension and ciliary length regulation. Dev Biol.

[188] Pugacheva EN, Jablonski SA, Hartman TR, Henske EP, Golemis EA (2007). HEF1-dependent Aurora A activation induces disassembly of the primary cilium. Cell.

[189] Ran J, Yang Y, Li D, Liu M, Zhou J (2015). Deacetylation of α-tubulin and cortactin is required for HDAC6 to trigger ciliary disassembly. Sci Rep.

[190] Izumi H, Li Y, Shibaki M, Mori D, Yasunami M, Sato S (2019). Recycling endosomal CD133 functions as an inhibitor of autophagy at the pericentrosomal region. Sci Rep.

[191] Marzesco AM, Janich P, Wilshch-Bräuninger M, Dubreuil V, Langenfeld K, Corbeil D (2005). Release of extracellular membrane particles carrying the stem cell marker prominin-1 (CD133) from neural progenitors and other epithelial cells. J Cell Sci.

[192] Dubreuil V, Marzesco AM, Corbeil D, Huttner WB, Wilsch-Brauninger M (2007). Midbody and primary cilium of neural progenitors release extracellular membrane particles enriched in the stem cell marker prominin-1. J Cell Biol.

[193] Mirzadeh Z, Merkle FT, Soriano-Navarro M, Garcia-Verdugo JM, Alvarez-Buylla A (2008). Neural stem cells confer unique pinwheel architecture to the ventricular surface in neurogenic regions of the adult brain. Cell Stem Cell.

[194] Codega P, Silva-Vargas V, Paul A, Maldonado-Soto AR, Deleo AM, Pastrana E (2014). Prospective identification and purification of quiescent adult neural stem cells from their in vivo niche. Neuron.

[195] Coskun V, Wu H, Blanchi B, Tsao S, Kim K, Zhao J (2008). CD133+ neural stem cells in the ependyma of mammalian postnatal forebrain. Proc Natl Acad Sci U S A.

[196] Zhong A, Short C, Xu J, Fernandez GE, Malkoff N, Noriega N (2023). Prominin-1 promotes restitution of the murine extrahepatic biliary luminal epithelium following cholestatic liver injury. Hepatol Commun.

[197] Jászai J, Corbeil D, Fargeas CA. Comprehensive overview of CD133 biology in neural tissues across species. In: Pruszak J, editor. Neural surface antigens–from basic biology towards biomedical applications. Academic Press; 2015. p. 113–129.

[198] Bachor TP, Karbanová J, Büttner E, Bermúdez V, Marquioni-Ramella M, Carmeliet P (2017). Early ciliary and prominin-1 dysfunctions precede neurogenesis impairment in a mouse model of type 2 diabetes. Neurobiol Dis.

[199] Serra CFH, Liu H, Qian J, Mori M, Lu J, Cardoso WV (2022). Prominin 1 and Notch regulate ciliary length and dynamics in multiciliated cells of the airway epithelium. iScience.

[200] Badano JL, Mitsuma N, Beales PL, Katsanis N (2006). The ciliopathies: an emerging class of human genetic disorders. Annu Rev Genomics Hum Genet.

[201] Xiao YS, Liang J, Gao M, Sun JR, Liu Y, Chen JQ (2021). Deletion of prominin-1 in mice results in disrupted photoreceptor outer segment protein homeostasis. Int J Ophthalmol.

[202] Fargeas CA, Jászai J, Corbeil D (2023). Prominin-1 expression in the testis/epididymis and fertility. Reprod Med Biol.

[203] Karim BO, Rhee KJ, Liu G, Yun K, Brant SR (2014). Prom1 function in development, intestinal inflammation, and intestinal tumorigenesis. Front Oncol.

[204] Nishide K, Nakatani Y, Kiyonari H, Kondo T (2009). Glioblastoma formation from cell population depleted of Prominin1-expressing cells. PLoS ONE.

[205] Jászai J, Fargeas CA, Haase M, Farkas LM, Huttner WB, Corbeil D (2008). Robust expression of Prominin-2 all along the adult male reproductive system and urinary bladder. Histochem Cell Biol.

[206] Asano A, Nelson JL, Zhang S, Travis AJ (2010). Characterization of the proteomes associating with three distinct membrane raft sub-types in murine sperm. Proteomics.

[207] Yukselten Y, Aydos OSE, Sunguroglu A, Aydos K (2019). Investigation of CD133 and CD24 as candidate azoospermia markers and their relationship with spermatogenesis defects. Gene.

[208] Matsukuma H, Kobayashi Y, Oka S, Higashijima F, Kimura K, Yoshihara E (2023). Prominin-1 deletion results in spermatogenic impairment, sperm morphological defects, and infertility in mice. Reprod Med Biol.

[209] Röper K, Corbeil D, Huttner WB (2000). Retention of prominin in microvilli reveals distinct cholesterol-based lipid micro-domains in the apical plasma membrane. Nat Cell Biol.

[210] Karbanová J, Lorico A, Bornhäuser M, Corbeil D, Fargeas CA (2018). Prominin-1/CD133: lipid raft association, detergent resistance, and immunodetection. Stem Cells Transl Med.

[211] Sezgin E, Levental I, Mayor S, Eggeling C (2017). The mystery of membrane organization: composition, regulation and roles of lipid rafts. Nat Rev Mol Cell Biol.

[212] Ikonen E, Simons K (1998). Protein and lipid sorting from the trans-Golgi network to the plasma membrane in polarized cells. Semin Cell Dev Biol.

[213] Huttner WB, Zimmerberg J (2001). Implications of lipid microdomains for membrane curvature, budding and fission. Curr Opin Cell Biol.

[214] Simons K, Toomre D (2000). Lipid rafts and signal transduction. Nat Rev Mol Cell Biol.

[215] Zhang S, Zhu N, Li HF, Gu J, Zhang CJ, Liao DF (2022). The lipid rafts in cancer stem cell: a target to eradicate cancer. Stem Cell Res Ther.

[216] Brown DA, Rose JK (1992). Sorting of GPI-anchored proteins to glycolipid-enriched membrane subdomains during transport to the apical cell surface. Cell.

[217] Lingwood D, Simons K (2007). Detergent resistance as a tool in membrane research. Nat Protoc.

[218] Corbeil D, Marzesco AM, Wilsch-Bräuninger M, Huttner WB (2010). The intriguing links between prominin-1 (CD133), cholesterol-based membrane microdomains, remodeling of apical plasma membrane protrusions, extracellular membrane particles, and (neuro)epithelial cell differentiation. FEBS Lett.

[219] Taïeb N, Maresca M, Guo X-J, Garmy N, Fantini J, Yahi N (2009). The first extracellular domain of the tumour stem cell marker CD133 contains an antigenic ganglioside-binding motif. Cancer Lett.

[220] Gillette JM, Larochelle A, Dunbar CE, Lippincott-Schwartz J (2009). Intercellular transfer to signalling endosomes regulates an ex vivo bone marrow niche. Nat Cell Biol.

[221] Freund D, Fonseca A-V, Janich P, Bornhäuser M, Corbeil D (2010). Differential expression of biofunctional GM1 and GM3 gangliosides within the plastic-adherent multipotent mesenchymal stromal cell population. Cytotherapy.

[222] Mazerik JN, Tyska MJ (2012). Myosin-1A targets to microvilli using multiple membrane binding motifs in the tail homology 1 (TH1) domain. J Biol Chem.

[223] Hokanson DE, Ostap EM (2006). Myo1c binds tightly and specifically to phosphatidylinositol 4,5-bisphosphate and inositol 1,4,5-trisphosphate. Proc Natl Acad Sci U S A.

[224] Insall RH, Weiner OD (2001). PIP3, PIP2, and cell movement—similar messages, different meanings?. Dev Cell.

[225] Spillane M, Ketschek A, Jones SL, Korobova F, Marsick B, Lanier L (2011). The actin nucleating Arp2/3 complex contributes to the formation of axonal filopodia and branches through the regulation of actin patch precursors to filopodia. Dev Neurobiol.

[226] Iglič A, Hägerstrand H, Veranič P, Plemenitaš A, Kralj-Iglič V (2006). Curvature-induced accumulation of anisotropic membrane components and raft formation in cylindrical membrane protrusions. J Theor Biol.

[227] Schara K, Jansa V, Sustar V, Dolinar D, Pavlic JI, Lokar M (2009). Mechanisms for the formation of membranous nanostructures in cell-to-cell communication. Cell Mol Biol Lett.

[228] Kabaso D, Bobrovska N, Góźdź W, Gov N, Kralj-Iglič V, Veranič P (2012). On the role of membrane anisotropy and BAR proteins in the stability of tubular membrane structures. J Biomech.

[229] Kinnebrew M, Iverson EJ, Patel BB, Pusapati GV, Kong JH, Johnson KA (2019). Cholesterol accessibility at the ciliary membrane controls hedgehog signaling. Elife.

[230] Radhakrishnan A, Rohatgi R, Siebold C (2020). Cholesterol access in cellular membranes controls Hedgehog signaling. Nat Chem Biol.

[231] Adhyapak P, Kapoor S (2019). Membrane dynamics in health and disease: impact on cellular signalling. J Membr Biol.

[232] Sasaki A, Kamiyama T, Yokoo H, Nakanishi K, Kubota K, Haga H (2010). Cytoplasmic expression of CD133 is an important risk factor for overall survival in hepatocellular carcinoma. Oncol Rep.

[233] Zhang J, Guo X, Chang DY, Rosen DG, Mercado-Uribe I, Liu J (2012). CD133 expression associated with poor prognosis in ovarian cancer. Mod Pathol.

[234] Brescia P, Ortensi B, Fornasari L, Levi D, Broggi G, Pelicci G (2013). CD133 is essential for glioblastoma stem cell maintenance. Stem Cells.

[235] Chen Y-L, Lin P-Y, Ming Y-Z, Huang W-C, Chen R-F, Chen P-M (2017). The effects of the location of cancer stem cell marker CD133 on the prognosis of hepatocellular carcinoma patients. BMC Cancer.

[236] Adini A, Adini I, Ghosh K, Benny O, Pravda E, Hu R (2013). The stem cell marker prominin-1/CD133 interacts with vascular endothelial growth factor and potentiates its action. Angiogenesis.

[237] Chen H, Luo Z, Dong L, Tan Y, Yang J, Feng G (2013). CD133/Prominin-1-mediated autophagy and glucose uptake beneficial for hepatoma cell survival. PLoS ONE.

[238] Sun H, Zhang M, Cheng K, Li P, Han S, Li R (2016). Resistance of glioma cells to nutrient-deprived microenvironment can be enhanced by CD133-mediated autophagy. Oncotarget.

[239] Bhattacharya S, Yin J, Winborn CS, Zhang Q, Yue J, Chaum E (2017). Prominin-1 is a novel regulator of autophagy in the human retinal pigment epithelium. Invest Ophthalmol Vis Sci.

[240] Cha-Molstad H, Yu JE, Feng Z, Lee SH, Kim JG, Yang P (2017). p62/SQSTM1/Sequestosome-1 is an N-recognin of the N-end rule pathway which modulates autophagosome biogenesis. Nat Commun.

[241] Weidberg H, Shvets E, Shpilka T, Shimron F, Shinder V, Elazar Z (2010). LC3 and GATE-16/GABARAP subfamilies are both essential yet act differently in autophagosome biogenesis. EMBO J.

[242] Joachim J, Jefferies HB, Razi M, Frith D, Snijders AP, Chakravarty P (2015). Activation of ULK kinase and autophagy by GABARAP trafficking from the centrosome is regulated by WAC and GM130. Mol Cell.

[243] Rogov V, Dötsch V, Johansen T, Kirkin V (2014). Interactions between autophagy receptors and ubiquitin-like proteins form the molecular basis for selective autophagy. Mol Cell.

[244] Fonseca AV, Bauer N, Corbeil D (2008). The stem cell marker CD133 meets the endosomal compartment–new insights into the cell division of hematopoietic stem cells. Blood Cells Mol Dis.

[245] Pine SR, Ryan BM, Varticovski L, Robles AI, Harris CC (2010). Microenvironmental modulation of asymmetric cell division in human lung cancer cells. Proc Natl Acad Sci U S A.

[246] Lathia JD, Hitomi M, Gallagher J, Gadani SP, Adkins J, Vasanji A (2011). Distribution of CD133 reveals glioma stem cells self-renew through symmetric and asymmetric cell divisions. Cell Death Dis.

[247] Izumi H, Li Y, Yasunami M, Sato S, Mae T, Kaneko Y (2022). Asymmetric pericentrosomal CD133 endosomes induce the unequal autophagic activity during cytokinesis in CD133-positive human neuroblastoma cells. Stem Cells.

[248] Hitomi M, Chumakova AP, Silver DJ, Knudsen AM, Pontius WD, Murphy S (2021). Asymmetric cell division promotes therapeutic resistance in glioblastoma stem cells. JCI Insight.

[249] Kosodo Y, Röper K, Haubensak W, Marzesco AM, Corbeil D, Huttner WB (2004). Asymmetric distribution of the apical plasma membrane during neurogenic divisions of mammalian neuroepithelial cells. EMBO J.

[250] Mizushima N, Levine B (2010). Autophagy in mammalian development and differentiation. Nat Cell Biol.

[251] Pampliega O, Orhon I, Patel B, Sridhar S, Díaz-Carretero A, Beau I (2013). Functional interaction between autophagy and ciliogenesis. Nature.

[252] Orhon I, Dupont N, Pampliega O, Cuervo AM, Codogno P (2015). Autophagy and regulation of cilia function and assembly. Cell Death Differ.

[253] Cao M, Zhong Q (2015). Cilia in autophagy and cancer. Cilia.

[254] Morleo F (2019). The autophagy-cilia axis: an intricate relationship. Cells.

[255] Nunukova A, Neradil J, Skoda J, Jaros J, Hampl A, Sterba J (2015). Atypical nuclear localization of CD133 plasma membrane glycoprotein in rhabdomyosarcoma cell lines. Int J Mol Med.

[256] Skoda J, Nunukova A, Loja T, Zambo I, Neradil J, Mudry P (2016). Cancer stem cell markers in pediatric sarcomas: Sox2 is associated with tumorigenicity in immunodeficient mice. Tumor Biol.

[257] Cantile M, Collina F, D'Aiuto M, Rinaldo M, Pirozzi G, Borsellino C (2013). Nuclear localization of cancer stem cell marker CD133 in triple-negative breast cancer: a case report. Tumori.

[258] Huang MJ, Zhu HJ, Feng J, Ni SS, Huang JF (2015). High CD133 expression in the nucleus and cytoplasm predicts poor prognosis in non-small cell lung cancer. Dis Markers.

[259] Rappa G, Santos MF, Green TM, Karbanová J, Hassler J, Bai Y (2017). Nuclear transport of cancer extracellular vesicle-derived biomaterials through nuclear envelope invagination-associated late endosomes. Oncotarget.

[260] Lee YM, Yeo MK, Seong IO, Kim KH (2018). Nuclear expression of CD133 is associated with good prognosis in patients with colorectal adenocarcinoma. Anticancer Res.

[261] Briscoe J, Thérond PP (2013). The mechanisms of Hedgehog signalling and its roles in development and disease. Nat Rev Mol Cell Biol.

[262] Tierney MT, Aydogdu T, Sala D, Malecova B, Gatto S, Puri PL (2014). STAT3 signaling controls satellite cell expansion and skeletal muscle repair. Nat Med.

[263] Bhattacharya S, Yin J, Yang C, Wang Y, Sims M, Pfeffer LM (2022). STAT3 suppresses the AMPKα/ULK1-dependent induction of autophagy in glioblastoma cells. J Cell Mol Med.

[264] Zheng H, Zhang Y, Chen Y, Guo P, Wang X, Yuan X (2019). Prominin-like, a homolog of mammalian CD133, suppresses di lp6 and TOR signaling to maintain body size and weight in Drosophila. FASEB J.

[265] Ryu TH, Yeom E, Subramanian M, Lee K-S, Yu K (2018). Prominin-like regulates longevity and glucose metabolism via insulin signaling in drosophila. J Gerontol A Biol Sci Med Sci.

[266] Sepp KJ, Hong P, Lizarraga SB, Liu JS, Mejia LA, Walsh CA (2008). Identification of neural outgrowth genes using genome-wide RNAi. PLoS Genet.

[267] Yang C, Yang Y, Gupta N, Liu X, He A, Liu L (2007). Pentaspan membrane glycoprotein, prominin-1, is involved in glucose metabolism and cytoskeleton alteration. Biochemistry (Mosc).

[268] Griguer CE, Oliva CR, Gobin E, Marcorelles P, Benos DJ, Lancaster JR (2008). CD133 is a marker of bioenergetic stress in human glioma. PLoS ONE.

[269] Zobalova R, Prokopova K, Stantic M, Stapelberg M, Dong LF, Ralph SJ (2011). The potential role of CD133 in immune surveillance and apoptosis: a mitochondrial connection?. Antioxid Redox Signal.

[270] Raposo G, Nijman HW, Stoorvogel W, Liejendekker R, Harding CV, Melief CJ (1996). B lymphocytes secrete antigen-presenting vesicles. J Exp Med.

[271] van Niel G, D'Angelo G, Raposo G (2018). Shedding light on the cell biology of extracellular vesicles. Nat Rev Mol Cell Biol.

[272] Arena GO, Forte S, Abdouh M, Vanier C, Corbeil D, Lorico A (2023). Horizontal transfer of malignant traits and the involvement of extracellular vesicles in metastasis. Cells.

[273] Yáñez-Mó M, Siljander PR, Andreu Z, Zavec AB, Borràs FE, Buzas EI (2015). Biological properties of extracellular vesicles and their physiological functions. J Extracell Vesicles.

[274] Raposo G, Stoorvogel W (2013). Extracellular vesicles: exosomes, microvesicles, and friends. J Cell Biol.

[275] Corbeil D, Santos MF, Karbanová J, Kurth T, Rappa G, Lorico A (2020). Uptake and fate of extracellular membrane vesicles: nucleoplasmic reticulum-associated late endosomes as a new gate to intercellular communication. Cells.

[276] Rilla K (2021). Diverse plasma membrane protrusions act as platforms for extracellular vesicle shedding. J Extracell Vesicles.

[277] Gustafson CM, Gammill LS (2022). Extracellular vesicles and membrane protrusions in developmental signaling. J Dev Biol.

[278] Marzesco AM, Wilsch-Bräuninger M, Dubreuil V, Janich P, Langenfeld K, Thiele C (2009). Release of extracellular membrane vesicles from microvilli of epithelial cells is enhanced by depleting membrane cholesterol. FEBS Lett.

[279] Corbeil D, Marzesco AM, Fargeas CA, Huttner WB (2010). Prominin-1: a distinct cholesterol-binding membrane protein and the organisation of the apical plasma membrane of epithelial cells. Subcell Biochem.

[280] de Poret A, Dibsy R, Merida P, Trausch A, Inamdar K, Muriaux D (2022). Extracellular vesicles containing the I-BAR protein IRSp53 are released from the cell plasma membrane in an Arp2/3 dependent manner. Biol Cell.

[281] Nager AR, Goldstein JS, Herranz-Pérez V, Portran D, Ye F, Garcia-Verdugo JM (2017). An actin network dispatches ciliary GPCRs into extracellular vesicles to modulate signaling. Cell.

[282] Ettinger AW, Wilsch-Bräuninger M, Marzesco A-M, Bickle M, Lohmann A, Maliga Z (2011). Proliferating versus differentiating stem and cancer cells exhibit distinct midbody-release behaviour. Nat Commun.

[283] McConnell RE, Higginbotham JN, Shifrin DA, Tabb DL, Coffey RJ, Tyska MJ (2009). The enterocyte microvillus is a vesicle-generating organelle. J Cell Biol.

[284] Muralidharan-Chari V, Clancy J, Plou C, Romao M, Chavrier P, Raposo G (2009). ARF6-regulated shedding of tumor cell-derived plasma membrane microvesicles. Curr Biol.

[285] Hurbain I, Macé A-S, Romao M, Prince E, Sengmanivong L, Ruel L (2022). Microvilli-derived extracellular vesicles carry Hedgehog morphogenic signals for Drosophila wing imaginal disc development. Curr Biol.

[286] Shenoy GN, Loyall J, Berenson CS, Kelleher RJ, Iyer V, Balu-Iyer SV (2018). Sialic acid-dependent inhibition of T cells by exosomal ganglioside GD3 in ovarian tumor microenvironments. J Immunol.

[287] Marzesco AM (2013). Prominin-1-containing membrane vesicles: origins, formation, and utility. Adv Exp Med Biol.

[288] Rappa G, Mercapide J, Anzanello F, Pope RM, Lorico A (2013). Biochemical and biological characterization of exosomes containing prominin-1/CD133. Mol Cancer.

[289] Rappa G, Mercapide J, Anzanello F, Le TT, Johlfs MG, Fiscus RR (2013). Wnt interaction and extracellular release of prominin-1/CD133 in human malignant melanoma cells. Exp Cell Res.

[290] Lucchetti D, Calapa F, Palmieri V, Fanali C, Carbone F, Papa A (2017). Differentiation affects the release of exosomes from colon cancer cells and their ability to modulate the behavior of recipient cells. Am J Pathol.

[291] Kang M, Kim S, Ko J (2019). Roles of CD133 in microvesicle formation and oncoprotein trafficking in colon cancer. FASEB J.

[292] Moon B-S, Jeong W-J, Park J, Kim TI, Min DS, Choi K-Y (2014). Role of oncogenic K-Ras in cancer stem cell activation by aberrant Wnt/β-catenin signaling. J Natl Cancer Inst.

[293] Deng Y, Wang L, Tan S, Kim GP, Dou R, Chen D (2015). KRAS as a predictor of poor prognosis and benefit from postoperative FOLFOX chemotherapy in patients with stage II and III colorectal cancer. Mol Oncol.

[294] Huttner HB, Janich P, Kohrmann M, Jászai J, Siebzehnrubl F, Blumcke I (2008). The stem cell marker prominin-1/CD133 on membrane particles in human cerebrospinal fluid offers novel approaches for studying central nervous system disease. Stem Cells.

[295] Huttner HB, Corbeil D, Thirmeyer C, Coras R, Köhrmann M, Mauer C (2012). Increased membrane shedding—indicated by an elevation of CD133-enriched membrane particles—into the CSF in partial epilepsy. Epilepsy Res.

[296] Bobinger T, May L, Lücking H, Kloska SP, Burkardt P, Spitzer P (2017). CD133-positive membrane particles in cerebrospinal fluid of patients with inflammatory and degenerative neurological diseases. Front Cell Neurosci.

[297] Bobinger T, Roeder SS, Spruegel MI, Froehlich K, Beuscher VD, Hoelter P (2020). Variation of membrane particle–bound CD133 in cerebrospinal fluid of patients with subarachnoid and intracerebral hemorrhage. J Neurosurg.

[298] Dimuccio V, Ranghino A, Praticò Barbato L, Fop F, Biancone L, Camussi G (2014). Urinary CD133+ extracellular vesicles are decreased in kidney transplanted patients with slow graft function and vascular damage. PLoS ONE.

[299] Dimuccio V, Peruzzi L, Brizzi MF, Cocchi E, Fop F, Boido A (2020). Acute and chronic glomerular damage is associated with reduced CD133 expression in urinary extracellular vesicles. Am J Physiol Renal Physiol.

[300] Burrello J, Monticone S, Burrello A, Bolis S, Cristalli CP, Comai G (2023). Identification of a serum and urine extracellular vesicle signature predicting renal outcome after kidney transplant. Nephrol Dial Transplant.

[301] Ranghino A, Dimuccio V, Papadimitriou E, Bussolati B (2015). Extracellular vesicles in the urine: markers and mediators of tissue damage and regeneration. Clin Kidney J.

[302] Rustom A, Saffrich R, Markovic I, Walther P, Gerdes HH (2004). Nanotubular highways for intercellular organelle transport. Science.

[303] Cordero Cervantes D, Zurzolo C (2021). Peering into tunneling nanotubes-The path forward. EMBO J.

[304] Kolba MD, Dudka W, Zaręba-Kozioł M, Kominek A, Ronchi P, Turos L (2019). Tunneling nanotube-mediated intercellular vesicle and protein transfer in the stroma-provided imatinib resistance in chronic myeloid leukemia cells. Cell Death Dis.

[305] Wang X, Gerdes HH (2015). Transfer of mitochondria via tunneling nanotubes rescues apoptotic PC12 cells. Cell Death Differ.

[306] Wang X, Veruki ML, Bukoreshtliev NV, Hartveit E, Gerdes HH (2010). Animal cells connected by nanotubes can be electrically coupled through interposed gap-junction channels. Proc Natl Acad Sci U S A.

[307] Wang X, Bukoreshtliev NV, Gerdes HH (2012). Developing neurons form transient nanotubes facilitating electrical coupling and calcium signaling with distant astrocytes. PLoS ONE.

[308] Reichert D, Scheinpflug J, Karbanová J, Freund D, Bornhäuser M, Corbeil D (2016). Tunneling nanotubes mediate the transfer of stem cell marker CD133 between hematopoietic progenitor cells. Exp Hematol.

[309] Rappa G, Fargeas CA, Le TT, Corbeil D, Lorico A (2015). Letter to the editor: an intriguing relationship between lipid droplets, cholesterol-binding protein CD133 and Wnt/β-catenin signaling pathway in carcinogenesis. Stem Cells.

[310] Jang JW, Song Y, Kim SH, Kim J, Seo HR (2017). Potential mechanisms of CD133 in cancer stem cells. Life Sci.

[311] Etienne-Manneville S, Hall A (2002). Rho GTPases in cell biology. Nature.

[312] Riento K, Ridley AJ (2003). Rocks: multifunctional kinases in cell behaviour. Nat Rev Mol Cell Biol.

[313] Amano M, Nakayama M, Kaibuchi K (2010). Rho-kinase/ROCK: a key regulator of the cytoskeleton and cell polarity. Cytoskeleton (Hoboken).

[314] Guan G, Cannon RD, Coates DE, Mei L (2023). Effect of the Rho-kinase/ROCK signaling pathway on cytoskeleton components. Genes (Basel).

[315] Li H, Papadopoulos V (1998). Peripheral-type benzodiazepine receptor function in cholesterol transport. Identification of a putative cholesterol recognition/interaction amino acid sequence and consensus pattern. Endocrinology.

[316] Fantini J, Barrantes FJ (2013). How cholesterol interacts with membrane proteins: an exploration of cholesterol-binding sites including CRAC, CARC, and tilted domains. Front Physiol.

[317] Fonseca A-V, Freund D, Bornhäuser M, Corbeil D (2010). Polarization and migration of hematopoietic stem and progenitor cells rely on the RhoA/ROCK I pathway and an active reorganization of the microtubule network. J Biol Chem.

[318] Fonseca A-V, Corbeil D (2011). The hematopoietic stem cell polarization and migration. Commun Integr Biol.

[319] Suzuki M (2006). The Drosophila tweety family: molecular candidates for large-conductance Ca2+ activated Cl- channels. Exp Physiol.

[320] Sukalskaia A, Straub MS, Deneka D, Sawicka M, Dutzler R (2021). Cryo-EM structures of the TTYH family reveal a novel architecture for lipid interactions. Nat Commun.

[321] Li B, Hoel CM, Brohawn SG (2021). Structures of tweety homolog proteins TTYH2 and TTYH3 reveal a Ca2+-dependent switch from intra- to intermembrane dimerization. Nat Commun.

[322] Melvin E, Kalaninová Z, Shlush E, Man P, Giladi M, Haitin Y (2022). TTYH family members form tetrameric complexes at the cell membrane. Commun Biol.

[323] Shimozato O, Waraya M, Nakashima K, Souda H, Takiguchi N, Yamamoto H (2015). Receptor-type protein tyrosine phosphatase κ directly dephosphorylates CD133 and regulates downstream AKT activation. Oncogene.

[324] Matsushita M, Mori Y, Uchiumi K, Ogata T, Nakamura M, Yoda H (2019). PTPRK suppresses progression and chemo-resistance of colon cancer cells via direct inhibition of pro-oncogenic CD133. FEBS Open Bio.

[325] Tonks NK (2006). Protein tyrosine phosphatases: from genes, to function, to disease. Nat Rev Mol Cell Biol.

[326] Xu Y, Tan L-J, Grachtchouk V, Voorhees JJ, Fisher GJ (2005). Receptor-type protein-tyrosine phosphatase-κ regulates epidermal growth factor receptor function. J Biol Chem.

[327] Liang Z, Wu B, Ji Z, Liu W, Shi D, Chen X (2021). The binding of LDN193189 to CD133 C-terminus suppresses the tumorigenesis and immune escape of liver tumor-initiating cells. Cancer Lett.

[328] Takenobu H, Shimozato O, Nakamura T, Ochiai H, Yamaguchi Y, Ohira M (2011). CD133 suppresses neuroblastoma cell differentiation via signal pathway modification. Oncogene.

[329] Fruman DA, Chiu H, Hopkins BD, Bagrodia S, Cantley LC, Abraham RT (2017). The PI3K pathway in human disease. Cell.

[330] Engelman JA, Luo J, Cantley LC (2006). The evolution of phosphatidylinositol 3-kinases as regulators of growth and metabolism. Nat Rev Genet.

[331] Vanhaesebroeck B, Guillermet-Guibert J, Graupera M, Bilanges B (2010). The emerging mechanisms of isoform-specific PI3K signalling. Nat Rev Mol Cell Biol.

[332] Hambardzumyan D, Squatrito M, Carbajal E, Holland EC (2008). Glioma formation, cancer stem cells, and akt signaling. Stem Cell Rev.

[333] Manning BD, Toker A (2017). AKT/PKB signaling: navigating the network. Cell.

[334] Vivanco I, Sawyers CL (2002). The phosphatidylinositol 3-Kinase–AKT pathway in human cancer. Nat Rev Cancer.

[335] Getz G, Gabriel SB, Cibulskis K, Lander E, Sivachenko A, Sougnez C (2013). Integrated genomic characterization of endometrial carcinoma. Nature.

[336] Bass AJ, Thorsson V, Shmulevich I, Reynolds SM, Miller M, Bernard B (2014). Comprehensive molecular characterization of gastric adenocarcinoma. Nature.

[337] Gao D, Vela I, Sboner A, Iaquinta PJ, Karthaus WR, Gopalan A (2014). Organoid cultures derived from patients with advanced prostate cancer. Cell.

[338] Lawrence MS, Sougnez C, Lichtenstein L, Cibulskisl K, Lander E, Gabriel SB (2015). Comprehensive genomic characterization of head and neck squamous cell carcinomas. Nature.

[339] Liu Y, Easton J, Shao Y, Maciaszek J, Wang ZM, Wilkinson MR (2017). The genomic landscape of pediatric and young adult T-lineage acute lymphoblastic leukemia. Nat Genet.

[340] Manoranjan B, Chokshi C, Venugopal C, Subapanditha M, Savage N, Tatari N (2020). A CD133-AKT-Wnt signaling axis drives glioblastoma brain tumor-initiating cells. Oncogene.

[341] Cross DA, Alessi DR, Cohen P, Andjelkovich M, Hemmings BA (1995). Inhibition of glycogen synthase kinase-3 by insulin mediated by protein kinase B. Nature.

[342] Shibahara I, Sonoda Y, Saito R, Kanamori M, Yamashita Y, Kumabe T (2013). The expression status of CD133 is associated with the pattern and timing of primary glioblastoma recurrence. Neuro Oncol.

[343] Wang Z, Liu W, Wang C, Li Y, Ai Z (2020). Acetylcholine promotes the self-renewal and immune escape of CD133+ thyroid cancer cells through activation of CD133-Akt pathway. Cancer Lett.

[344] Jamal SME, Alamodi A, Wahl RU, Grada Z, Shareef MA, Hassan S-Y (2020). Melanoma stem cell maintenance and chemo-resistance are mediated by CD133 signal to PI3K-dependent pathways. Oncogene.

[345] Brabletz T, Kalluri R, Nieto MA, Weinberg RA (2018). EMT in cancer. Nat Rev Cancer.

[346] Olea-Flores M, Zuñiga-Eulogio M, Tacuba-Saavedra A, Bueno-Salgado M, Sánchez-Carvajal A, Vargas-Santiago Y (2019). Leptin promotes expression of EMT-related transcription factors and invasion in a Src and FAK-dependent pathway in MCF10A mammary epithelial cells. Cells.

[347] Tian Q, Yuan P, Quan C, Li M, Xiao J, Zhang L (2020). Phosphorylation of BCKDK of BCAA catabolism at Y246 by Src promotes metastasis of colorectal cancer. Oncogene.

[348] Patel A, Sabbineni H, Clarke A, Somanath PR (2016). Novel roles of Src in cancer cell epithelial-to-mesenchymal transition, vascular permeability, microinvasion and metastasis. Life Sci.

[349] Tai Y-L, Chen L-C, Shen T-L (2015). Emerging roles of focal adhesion kinase in cancer. Biomed Res Int.

[350] Lee BY, Timpson P, Horvath LG, Daly RJ (2015). FAK signaling in human cancer as a target for therapeutics. Pharmacol Ther.

[351] Tai Y-L, Chu P-Y, Lai IR, Wang M-Y, Tseng H-Y, Guan J-L (2015). An EGFR/Src-dependent β4 integrin/FAK complex contributes to malignancy of breast cancer. Sci Rep.

[352] Liu C, Li Y, Xing Y, Cao B, Yang F, Yang T (2016). The interaction between cancer stem cell marker CD133 and Src protein promotes focal adhesion kinase (FAK) phosphorylation and cell migration. J Biol Chem.

[353] Nusse R, Brown A, Papkoff J, Scambler P, Shackleford G, McMahon A (1991). A new nomenclature for int-1 and related genes: the Wnt gene family. Cell.

[354] Logan CY, Nusse R (2004). The Wnt signaling pathway in development and disease. Annu Rev Cell Dev Biol.

[355] Nusse R, Clevers H (2017). Wnt/β-catenin signaling, disease, and emerging therapeutic modalities. Cell.

[356] Klaus A, Birchmeier W (2008). Wnt signalling and its impact on development and cancer. Nat Rev Cancer.

[357] Sawa H (2012). Control of cell polarity and asymmetric division in *C. elegans*. Curr Top Dev Biol.

[358] Huelsken J, Vogel R, Erdmann B, Cotsarelis G, Birchmeier W (2001). beta-Catenin controls hair follicle morphogenesis and stem cell differentiation in the skin. Cell.

[359] Gay DL, Yang CC, Plikus MV, Ito M, Rivera C, Treffeisen E (2015). CD133 expression correlates with membrane beta-catenin and E-cadherin loss from human hair follicle placodes during morphogenesis. J Invest Dermatol.

[360] Brossa A, Papadimitriou E, Collino F, Incarnato D, Oliviero S, Camussi G (2018). Role of CD133 molecule in Wnt response and renal repair. Stem Cells Transl Med.

[361] Tremblay JR, Lopez K, Ku HT (2019). A GLIS3–CD133–WNT-signaling axis regulates the self-renewal of adult murine pancreatic progenitor-like cells in colonies and organoids. J Biol Chem.

[362] Mak AB, Nixon AML, Kittanakom S, Stewart JM, Chen GI, Curak J (2012). Regulation of CD133 by HDAC6 promotes β-catenin signaling to suppress cancer cell differentiation. Cell Rep.

[363] Rappa G, Fodstad O, Lorico A (2008). The stem cell-associated antigen CD133 (Prominin-1) is a molecular therapeutic target for metastatic melanoma. Stem Cells.

[364] Hubbert C, Guardiola A, Shao R, Kawaguchi Y, Ito A, Nixon A (2002). HDAC6 is a microtubule-associated deacetylase. Nature.

[365] Li Y, Zhang X, Polakiewicz RD, Yao TP, Comb MJ (2008). HDAC6 is required for epidermal growth factor-induced beta-catenin nuclear localization. J Biol Chem.

[366] Miyake Y, Keusch JJ, Wang LL, Saito M, Hess D, Wang XN (2016). Structural insights into HDAC6 tubulin deacetylation and its selective inhibition. Nat Chem Biol.

[367] Kawaguchi Y, Kovacs JJ, McLaurin A, Vance JM, Ito A, Yao TP (2003). The deacetylase HDAC6 regulates aggresome formation and cell viability in response to misfolded protein stress. Cell.

[368] Haggarty SJ, Koeller KM, Wong JC, Grozinger CM, Schreiber SL (2003). Domain-selective small-molecule inhibitor of histone deacetylase 6 (HDAC6)-mediated tubulin deacetylation. Proc Natl Acad Sci U S A.

[369] Deribe YL, Wild P, Chandrashaker A, Curak J, Schmidt MHH, Kalaidzidis Y (2009). Regulation of epidermal growth factor receptor trafficking by lysine deacetylase HDAC6. Sci Signal..

[370] David CJ, Massagué J (2018). Contextual determinants of TGFβ action in development, immunity and cancer. Nat Rev Mol Cell Biol.

[371] Lee J, Shin JE, Lee B, Kim H, Jeon Y, Ahn SH (2020). The stem cell marker Prom1 promotes axon regeneration by down-regulating cholesterol synthesis via Smad signaling. Proc Natl Acad Sci U S A.

[372] Lee H, Yu D-M, Bahn M-S, Kwon Y-J, Um MJ, Yoon SY (2022). Hepatocyte-specific Prominin-1 protects against liver injury-induced fibrosis by stabilizing SMAD7. Exp Mol Med.

[373] Nakao A, Afrakhte M, Morén A, Nakayama T, Christian JL, Heuchel R (1997). Identification of Smad7, a TGFbeta-inducible antagonist of TGF-beta signalling. Nature.

[374] Hayashi H, Abdollah S, Qiu Y, Cai J, Xu YY, Grinnell BW (1997). The MAD-related protein Smad7 associates with the TGFbeta receptor and functions as an antagonist of TGFbeta signaling. Cell.

[375] Fenlon M, Short C, Xu J, Malkoff N, Mahdi E, Hough M (2020). Prominin-1-expressing hepatic progenitor cells induce fibrogenesis in murine cholestatic liver injury. Physiol Rep.

[376] Schmidt-Arras D, Rose-John S (2016). IL-6 pathway in the liver: from physiopathology to therapy. J Hepatol.

[377] Bahn M-S, Yu D-M, Lee M, Jo S-J, Lee J-W, Kim H-C (2022). Central role of Prominin-1 in lipid rafts during liver regeneration. Nat Commun.

[378] Deming PB, Campbell SL, Stone JB, Rivard RL, Mercier AL, Howe AK (2015). Anchoring of protein kinase A by ERM (Ezrin-Radixin-Moesin) proteins is required for proper netrin signaling through DCC (deleted in colorectal cancer). J Biol Chem.

[379] Dema A, Perets E, Schulz MS, Deák VA, Klussmann E (2015). Pharmacological targeting of AKAP-directed compartmentalized cAMP signalling. Cell Signal.

[380] Lavoie H, Gagnon J, Therrien M (2020). ERK signalling: a master regulator of cell behaviour, life and fate. Nat Rev Mol Cell Biol.

[381] Ding Q, Miyazaki Y, Tsukasa K, Matsubara S, Yoshimitsu M, Takao S (2014). CD133 facilitates epithelial-mesenchymal transition through interaction with the ERK pathway in pancreatic cancer metastasis. Mol Cancer.

[382] Xin B, He X, Wang J, Cai J, Wei W, Zhang T (2016). Nerve growth factor regulates CD133 function to promote tumor cell migration and invasion via activating ERK1/2 signaling in pancreatic cancer. Pancreatology.

[383] Zhang L, Zhang L, Li H, Ge C, Zhao F, Tian H (2016). CXCL3 contributes to CD133+ CSCs maintenance and forms a positive feedback regulation loop with CD133 in HCC via Erk1/2 phosphorylation. Sci Rep.

[384] Vora P, Venugopal C, Salim SK, Tatari N, Bakhshinyan D, Singh M (2020). The rational development of CD133-targeting immunotherapies for glioblastoma. Cell Stem Cell.

